# LoBLH6 interacts with LoMYB65 to regulate anther development through feedback regulation of gibberellin synthesis in lily

**DOI:** 10.1093/hr/uhae339

**Published:** 2024-12-04

**Authors:** Junpeng Yu, Ze Wu, Xinyue Liu, Qianqian Fang, Xue Pan, Sujuan Xu, Man He, Jinxing Lin, Nianjun Teng

**Affiliations:** Key Laboratory of Landscaping, Ministry of Agriculture and Rural Affairs, Key Laboratory of Biology of Ornamental Plants in East China, National Forestry and Grassland Administration, College of Horticulture, Nanjing Agricultural University, No. 1, Weigang, Xuanwu District, Nanjing 210095, China; Lily Science and Technology Backyard Qixia of Jiangsu/Jiangsu Graduate Workstation, Waisha Village, Baguazhou Street, Qixia District, Nanjing 210043, China; Key Laboratory of Landscaping, Ministry of Agriculture and Rural Affairs, Key Laboratory of Biology of Ornamental Plants in East China, National Forestry and Grassland Administration, College of Horticulture, Nanjing Agricultural University, No. 1, Weigang, Xuanwu District, Nanjing 210095, China; Lily Science and Technology Backyard Qixia of Jiangsu/Jiangsu Graduate Workstation, Waisha Village, Baguazhou Street, Qixia District, Nanjing 210043, China; Key Laboratory of Landscaping, Ministry of Agriculture and Rural Affairs, Key Laboratory of Biology of Ornamental Plants in East China, National Forestry and Grassland Administration, College of Horticulture, Nanjing Agricultural University, No. 1, Weigang, Xuanwu District, Nanjing 210095, China; Lily Science and Technology Backyard Qixia of Jiangsu/Jiangsu Graduate Workstation, Waisha Village, Baguazhou Street, Qixia District, Nanjing 210043, China; Key Laboratory of Landscaping, Ministry of Agriculture and Rural Affairs, Key Laboratory of Biology of Ornamental Plants in East China, National Forestry and Grassland Administration, College of Horticulture, Nanjing Agricultural University, No. 1, Weigang, Xuanwu District, Nanjing 210095, China; Lily Science and Technology Backyard Qixia of Jiangsu/Jiangsu Graduate Workstation, Waisha Village, Baguazhou Street, Qixia District, Nanjing 210043, China; Key Laboratory of Landscaping, Ministry of Agriculture and Rural Affairs, Key Laboratory of Biology of Ornamental Plants in East China, National Forestry and Grassland Administration, College of Horticulture, Nanjing Agricultural University, No. 1, Weigang, Xuanwu District, Nanjing 210095, China; Lily Science and Technology Backyard Qixia of Jiangsu/Jiangsu Graduate Workstation, Waisha Village, Baguazhou Street, Qixia District, Nanjing 210043, China; Key Laboratory of Landscaping, Ministry of Agriculture and Rural Affairs, Key Laboratory of Biology of Ornamental Plants in East China, National Forestry and Grassland Administration, College of Horticulture, Nanjing Agricultural University, No. 1, Weigang, Xuanwu District, Nanjing 210095, China; Lily Science and Technology Backyard Qixia of Jiangsu/Jiangsu Graduate Workstation, Waisha Village, Baguazhou Street, Qixia District, Nanjing 210043, China; Key Laboratory of Landscaping, Ministry of Agriculture and Rural Affairs, Key Laboratory of Biology of Ornamental Plants in East China, National Forestry and Grassland Administration, College of Horticulture, Nanjing Agricultural University, No. 1, Weigang, Xuanwu District, Nanjing 210095, China; Lily Science and Technology Backyard Qixia of Jiangsu/Jiangsu Graduate Workstation, Waisha Village, Baguazhou Street, Qixia District, Nanjing 210043, China; Beijing Advanced Innovation Center for Tree Breeding by Molecular Design, Beijing Forestry University, No. 35, East Qinghua Road, Haidian District, Beijing 100083, China; College of Biological Sciences and Biotechnology, Beijing Forestry University, No. 35, East Qinghua Road, Haidian District, Beijing 100083, China; Key Laboratory of Landscaping, Ministry of Agriculture and Rural Affairs, Key Laboratory of Biology of Ornamental Plants in East China, National Forestry and Grassland Administration, College of Horticulture, Nanjing Agricultural University, No. 1, Weigang, Xuanwu District, Nanjing 210095, China; Lily Science and Technology Backyard Qixia of Jiangsu/Jiangsu Graduate Workstation, Waisha Village, Baguazhou Street, Qixia District, Nanjing 210043, China

## Abstract

The homeostasis of gibberellin (GA) is crucial for the normal development of anthers, but its underlying regulatory mechanisms are not clear. The GA-induced v-Myb myeloblastosis viral oncogene homolog (MYB) transcription factor LoMYB65 is involved in anther development. In this study, we screened and identified an interacting protein of LoMYB65, *Lilium* Oriental Hybrids BEL1-Like Homeodomain6 (LoBLH6). LoBLH6 was localized in both the nucleus and cytoplasm, and it interacted with LoMYB65 through its BELL domain, exhibiting transcriptional repression activity. *LoBLH6* was continuously expressed during anther development, with particularly high expression in the mid and late stages. *In situ* hybridization revealed high expression of *LoBLH6* in the tapetum and microspores, with the same tissue specificity as *LoMYB65*. Silencing of *LoBLH6* in lilies resulted in abnormal anther development, reduced pollen, and increased GA content. The application of GA-induced phenotypes in the anthers and pollen of lily that were similar to the silencing of *LoBLH6*. Further research showed that LoBLH6 directly binds to the promoter of *Lilium* Oriental Hybrids *GA 20-oxidase1* (*LoGA20ox1*) to suppress its expression, and coexpression with LoMYB65 enhances this repression. Additionally, GA treatment enhanced the interaction between LoBLH6 and LoMYB65 and their complex's inhibitory effect on downstream target genes. During the transition from microspores to mature pollen grains in lily anthers, GA levels maintain a steady state, which is disrupted by silencing *LoBLH6*, leading to abnormal pollen development. Overall, our results reveal that the interaction between LoBLH6 and LoMYB65 regulates anther development through feedback regulation of GA synthesis.

## Introduction

In flowering plants, the normal development of anthers is a prerequisite for completing sexual reproduction, which is a complex biological process regulated by various factors. In the early stages of anther development, stamen primordial cells differentiate and divide into four layers (epidermis, endothecial cell layer, middle layer, tapetal layer), enclosing the microspore mother cells (MMCs) in the center. The MMCs are generated from sporogenous cells, undergo meiosis to produce haploid microspores, and subsequently undergo mitosis to ultimately develop into mature pollens [1, 2]. Lily, among the most renowned bulbous flowers globally, is valued for its ornamental qualities. Lily possesses large anthers and abundant pollens [3], making them an excellent material for exploring anther development. As the anther dehisces, lily pollen disperses onto the petals, affecting aesthetics and causing pollen-related trouble such as clothing contamination and allergic reactions [4]. Studying anther development in lily not only contributes to a deeper understanding of this essential biological process in the plant kingdom but also offers insights for the creation of pollenless lily varieties.

Although numerous plant hormones have been shown to participate in regulating various aspects of flower development, the role of gibberellins (GAs) is particularly prominent, especially in anther development. GA signals induce extensive transcriptional changes in downstream genes by relieving the transcriptional repression exerted by the DELLA protein family [5]. As the development of the anther is a fine regulated process [6], thus it remains a crucial scientific question how plants maintain GA at an appropriate level within the anther. v-Myb myeloblastosis viral oncogene homolog (MYB) transcription factors (TFs) is a big family of transcription factors in plants and plays an important role in many life activities of plants [7]. GAMYBs, R2R3-MYB TFs, are expressed in response to GA and are post-transcriptionally regulated by microRNA159 (miR159), which are core factors in GA-mediated anther developmental regulation in different plants [8–10]. Loss of *GAMYB*s leads to abnormalities in Ubisch bodies, programmed cell death (PCD) of tapetal layer cells, and meiosis of microspores, ultimately resulting in male sterility [11]. In lilies (*Lilium* Oriental Hybrids), GAMYB is also an important regulator of pollen development. Liu et al. identified two GAMYB members, LoMYB33 and LoMYB65. Silencing either *LoMYB33* or *LoMYB65* alone, or silencing both, results in reduced pollen quantity and the production of abnormal pollen in lilies [3, 12]. Despite GAMYB being a crucial component downstream of the GA signaling pathway, there have been few reports regarding its regulatory role in GA synthesis.

Three-amino Acid Loop Extension (TALE) is a class of transcription factors of plants. The BEL1-Like Homeodomain (BELL or BLH) family is a branch of the TALE superfamily, all members containing a typical Homeobox domain (HD). Ahead of HD, there is a POX domain composed of two conserved motifs, SKY and BELL [13, 14]. The HD serves as a DNA-binding domain. The POX domain can specifically interact with the MEINOX domain of KNOTTED1-like Homeobox (KNOX) transcription factors, another branch of the TALE superfamily, forming BLH-KNOX heterodimers that exert biological functions [15]. BLH transcription factors are widely involved in plant development and responses to the environment signals, including morphogenesis, flower differentiation, ovule and fruit development, cell wall formation, light response, as well as responses to environmental stresses including salt stress and heat stress [16–25]. In spite of this, research involving BLH members in anther development remains scarce.

In this study, we identified a novel BLH transcription factor, LoBLH6, from lily. *LoBLH6* was induced by bioactive GA in anther and LoBLH6 interacted with LoMYB65, engaging in a negative feedback regulation of GA synthesis, which contributed to maintain GA homeostasis and ensure the normal development of microspores and anther walls to produce fertile pollen.

## Results

### LoBLH6 interacts with LoMYB65

In a previous study, we confirmed the GAMYB transcription factor, LoMYB65, plays a positive role in lily pollen development [3]. Here, with a yeast two-hybrid (Y2H) screening, we identified a protein interactor of LoMYB65 that belonged to the BLH family. Phylogenetic analysis indicated that the BLH member was most closely related to the Arabidopsis AtBLH6, and thus, we named this protein LoBLH6. The coding sequence (CDS) of *LoBLH6* contained an open reading frame (ORF) of 1941 bp, encoding a protein of 646 amino acid residues. LoBLH6 possessed an HD and a POX domain. Behind the HD, there was a leucine-rich region 'VSLTLGL' (Fig. S1).

By fusing green fluorescence protein (GFP) tag at the N- and C-termini of the LoBLH6 protein and transiently expressing them in *Nicotiana benthamiana* leaves, we observed GFP signals distributed in both the nucleus and cytoplasm, which indicated LoBLH6 was a nucleus–cytoplasm localized protein (Fig. S2). LoBLH6 shares the same subcellular localization as LoMYB65 [3]. We conducted transcriptional activity experiments in yeast and tobacco and found that LoBLH6 alone can inhibit transcription, and the fusion of LoBLH6 with the transcriptional activator VP16 can suppress VP16's transcriptional activation (Fig. S3a–3e). These results suggest that LoBLH6 may function as a transcriptional repressor to regulate the transcription of downstream genes.

**Figure 1:**
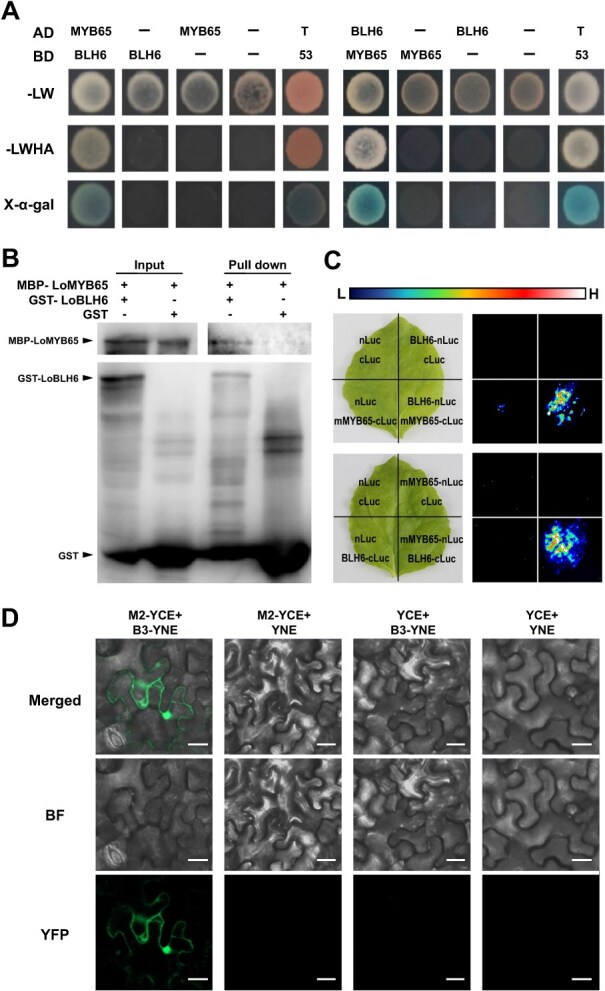
LoBLH6 interacts with LoMYB65 and *LoBLH6* coexpressed with *LoMYB65* in anthers. **(*A*)** Y2H assay. Yeast strains cotransformed with the pGADT7-T and pGBKT7–53 vectors served as the positive control group. The existence of protein–protein interaction was evaluated based on the growth of yeast on the medium lacking Leu, Trp, His, and Ade (-LWHA). The representative image is from three independent experiments. AD, pGADT7 vector; BD, pGBKT7 vector; MYB65, LoMYB65; BLH6, LoBLH6. **(*B*)** Pull-down assay to detect the interaction between LoBLH6 and LoMYB65. GST-LoBLH6 was used to pull down MBP-LoMYB65, with GST used as a negative control. `Input' denotes the protein mixtures utilized prior to the experiment, while `Pull-down' refers to the purified protein mixture. The symbol `+' signifies the presence, whereas `−' signifies the absence. GST, glutathione S-transferase tag; MBP, maltose-binding protein tag. **(*C*)** LCI assay of LoBLH6 with LoMYB65 in *N. benthamiana* leaves, and their LUC signals were assessed using Tanon bioluminescence imaging. The representative picture based on three biological replicates. cLuc, pCAMBIA1300-cLuc vector; LCI, LUC complementation imaging; LUC, luciferase; nLuc, pCAMBIA1300-nLuc vector; mMYB65, mLoMYB65; BLH6, LoBLH6. **(*D*)** bimolecular fluorescence complementation (BiFC) assay. The M2 region of LoMYB65 and the B3 region of LoBLH6 are fused with the C-terminal region (YCE) and N-terminal region (YNE) of yellow fluorescent protein (YFP) respectively. If an interaction occurs, YFP protein reassembly will take place in the interactive area, leading to the generation of fluorescent signals. Three experiments were performed, and one representative picture is shown. Scale bar = 20 μm. YCE, pSPYCE(M); YNE, pSPYNE173; B3, fragment 3 of LoBLH6 (237–392 aa); M2, fragment 2 of LoMYB65 (167–439 aa).

Subsequently, we confirmed the protein interaction of LoMYB65 and LoBLH6 with a point-to-point Y2H assay. The results showed that all transformed yeasts grew well on SD-LW medium, and both the positive control (pGADT7-T + pGBKT7–53) and LoMYB65-LoBLH6 groups could grow on SD-LWHA medium and catalyzed X-α-gal, confirming the interaction between LoBLH6 and LoMYB65 (Fig. 1a). And we found that it is the region between the BELL domain of LoBLH6 (B3) and the box1 and box2 region of LoMYB65 (M2) that interacts (Fig. S4). To further confirm the direct physical proximity between LoBLH6 and LoMYB65, we conducted pull-down assay. The results showed that GST-LoBLH6 could pull down MBP-LoMYB65, while GST could not (Fig. 1b), indicating a direct physical interaction between LoBLH6 and LoMYB65 *in vitro*. As the transcript of *GAMYBs* would be degraded by miR159s in plant cells [26], we created a mutation in the miR159 target site of *LoMYB65* without altering the amino acid sequence, resulting in a mutant version named *mLoMYB65* [3]. This allowed us to perform luciferase complementation imaging (LCI). In regions of *N. benthamiana* leaves coexpressing mLoMYB65 and LoBLH6, fluorescence signals can be observed, indicating an interaction between mLoMYB65 and LoBLH6 (Fig. 1c). Possibly, the fusion of LoBLH6, LoMYB65 with YCE, and YNE had an adverse impact on protein folding, leading to no significant YFP fluorescence observed in the bimolecular fluorescence complementation (BiFC) experiment with full-length proteins of LoBLH6 and LoMYB65. Therefore, we connected the interaction regions of LoBLH6 (B3) and LoMYB65 (M2) with YNE and YCE to form fusion proteins, and conducted a BiFC experiment in *N. benthamiana*. During the experiment, we observed that the B3 region of LoBLH6 interacts with the M2 region of LoMYB65 in the nucleus and cytoplasm (Fig. 1d).

### LoBLH6 and LoMYB65 are coexpressed in the anthers

**Figure 2:**
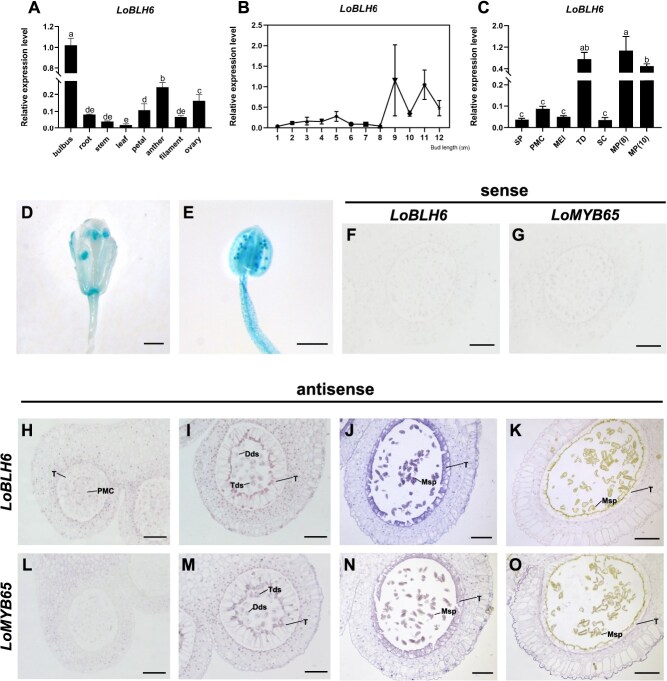
*LoBLH6* was highly expressed in the anthers. **(*A*)**  *LoBLH6* expression levels in various tissues of `Siberia' with a bud length of 3 cm. Data are the mean ± SD of three biological repeats. The different letters indicated statistically significant differences (Student–Newman–Keuls test, *P* < .05). **(*B*)** The expression of *LoBLH6* in anthers at different flower bud length (1–12 cm). Data are means ± SD of three biological repeats. **(*C*)** The relative expression level of *LoBLH6* in male reproductive cells at different development stage. Data are the mean ± SD of three biological repeats, with different letters indicating statistically significant difference (Student–Newman–Keuls test, *P* < .05). SP, sporulation period; PMC, pollen mother cell stage; MEI, meiotic stage; TD, tetrad stage; SC, single-core stage; MP (8), mature pollen in 8 cm buds; MP (10), mature pollen in 10-cm buds. GUS staining assay in *A. thaliana* flower **(*D*)** (scale bar = 1 mm) and anther **(*E*)** (scale bar = 200 μm) of *proLoBLH6*-GUS transgenic plants, GUS, glucuronidase. **(*F*)–(*O*)** RNA *in situ* hybridization of *LoBLH6* and *LoMYB65* in lily anther. **(*H*)** and **(*L*)** represent anther sections from flower buds with a length of 3 cm; **(*I*)** and **(*M*)** represent anther sections from flower buds with a length of 4.5 cm; **(*J*)** and **(*N*)** represent anther sections from flower buds with a length of 5 cm; **(*K*)** and **(*O*)** represent anther sections from flower buds with a length of 7 cm. **(*F*)** and **(*G*)** are anther sections from 4.5 cm flower buds incubated with *LoBLH6* and *LoMYB65* sense probes as negative controls. PMC, pollen mother cell; T, tapetum; Dds, dyads; Tds, tetrads; Msp, microspores. Scale bar = 200 μm.

Previous studies have shown that the anther development stages of the lily cultivar `Siberia' are closely correlated with bud length [27]. By comparing the anther development process of `Siberia' with that of the model plant Arabidopsis [ 28], we can categorize the stages as follows: bud length ≤ 1 cm corresponds to stages (S) 1–2 in Arabidopsis, 1 cm ≤ bud length ≤ 2 cm corresponds to S2–3, 2 cm ≤ bud length ≤ 3 cm corresponds to S4–5, bud length of 4 cm corresponds to S6, 4 cm ≤ bud length ≤ 5 cm corresponds to S7–8, 6 cm ≤ bud length ≤ 7 cm corresponds to S9, 8 cm ≤ bud length ≤ 9 cm corresponds to S10–11, and bud length ≥ 10 cm corresponds to S12–14 in Arabidopsis. To investigate the tissue-specific expression of *LoBLH6*, we performed a quantitative Real-Time Polymerase Chain Reaction (qRT-PCR) analysis of various lily tissues with flower bud length of 4 cm. It was found that, except for the bulb, the expression of *LoBLH6* was highest in the anthers (Fig. 2a). The *LoBLH6* was fluctuatingly expressed during anther development, showing a small expression peak in the anthers of 5-cm buds and continuously high expression in the later stages of anther development (Fig. 2b). The expression of *LoBLH6* was also analyzed in the pollen of `Siberia' at different developmental stages. The results showed that *LoBLH6* was high expression at the tetrad (TD) stage, similar to *LoMYB65* [3]. Additionally, *LoBLH6* was also highly expressed at the mature pollen stages (Fig. 2c). Analysis of the activity of the *LoBLH6* promoter in transgenic Arabidopsis revealed anther specific initiation of GUS reporter expression during the mid to late stages of anther development (Fig. 2d). Concurrently, strong activity was also observed in pollen (Fig. 2e), consistent with the gene expression analysis results. Further RNA *in situ* hybridization assay showed that in the early stages of anther development (3 and 4.5 cm), *LoBLH6* was expressed throughout the entire anther, with higher expression in the dyads, tetrads, and tapetal cells (Fig. 2f, 2h and 2i). By the 5-cm stage, *LoBLH6* showed its highest expression in the tapetum and microspores (Fig. 2j). By the 7-cm stage, *LoBLH6* exhibited lower expression levels in the epidermis, endothecium, and middle layer of the anther, with almost no expression in the tapetum and single-core microspores (Fig. 2k). *LoMYB65* was nearly undetectable in the pollen mother cells and tapetal cells of the 3-cm stage (Fig. 2l), but at later stages, its spatiotemporal expression pattern was consistent with that of *LoBLH6* (Fig. 2m–2o). This implies that LoMYB65 and LoBLH6 may participate in the regulation of the middle and later stages of anther development through protein interaction in the region of coexpression.

### Silencing of *LoBLH6* leads to lily pollen abortion

**Figure 3:**
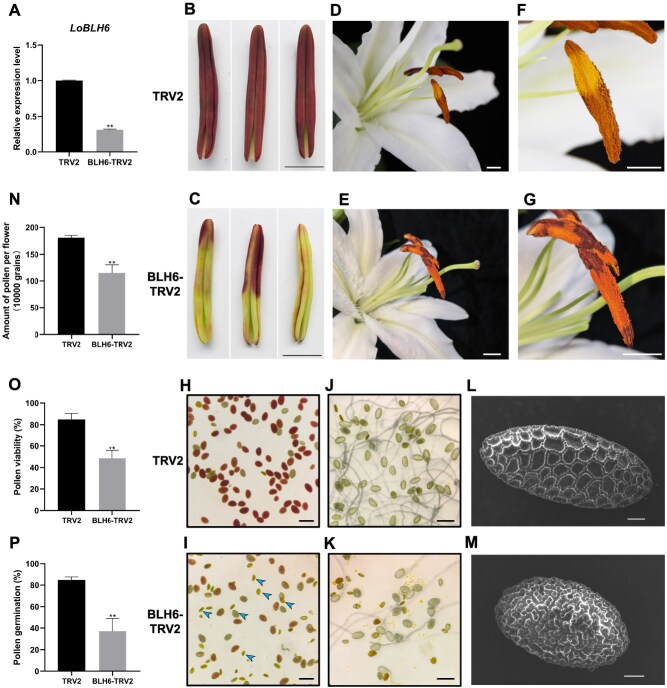
Silencing *LoBLH6* results in pollen sterility in lily. **(*A*)** Expression of *LoBLH6* in TRV2 and *LoBLH6* silenced lines (mean ± SD, *n* = 3). Asterisks indicate significant differences (Student's *t*-test, ^**^  *P* < .01). **(*B*)** and **(*C*)** depict anthers at the early stage of flower bud opening in `Siberia' for TRV2 and BLH6-TRV2, respectively. Scale bar =1 cm. **(*D*)** and **(*E*)** show the appearance of anthers at full bloom in `Siberia' for TRV2 and BLH6-TRV2, respectively. TRV indicates tobacco rattle virus, TRV2 indicates the blank control, and BLH6-TRV2 indicates *LoBLH6* silenced plants. Scale bar = 1 cm. **(*F*)** and **(*G*)** are magnified views of the anthers in (*B*) and (*C*), illustrating the difference in mature pollen states. Scale bar = 1 cm. **(*H*)** and **(*I*)** represent the vitality of mature pollen in `Siberia' for TRV2 and BLH6-TRV2, respectively, using TTC staining. Dark red indicates high pollen vitality, while light red or lack of color indicates low or no vitality; the arrows indicate some pollen with no vitality. Scale bar = 100 μm. **(*J*)** and **(*K*)** show the germination status of mature pollen in `Siberia' for TRV2 and BLH6-TRV2, respectively. Fresh pollen was suspended in liquid pollen germination medium and spread on solid germination medium. Photographs were taken under a microscope after 8 h, with pollen tube lengths greater than pollen lengths recorded as normally germinated pollen. Scale bar = 100 μm. **(*L*)** and **(*M*)** present SEM images of mature pollen for TRV2 and BLH6-TRV2, respectively. Scale bar = 10 μm. **(*N*)** Pollen quantity statistics for each flower in `Siberia' for TRV2 and BLH6-TRV2. Data are the mean ± SD of three biological repeats. Asterisks indicate significant differences (Student's *t*-test, ^**^  *P* < .01). **(*O*)** Vitality statistics of mature pollen in `Siberia' for TRV2 and BLH6-TRV2. Data are the mean ± SD of three biological repeats. Asterisks indicate significant differences (Student's *t*-test, ^**^  *P* < .01). **(*P*)** Germination rate statistics of mature pollen in `Siberia' for TRV2 and BLH6-TRV2. Data are the mean ± SD of three biological repeats. Asterisks indicate significant differences (Student's *t*-test, ^**^  *P* < .01).

Using Virus-Induced Gene Silencing (VIGS) techniques, we successfully silenced the expression of *LoBLH6* in `Siberia' anthers (Fig. 3a). Observation of the anthers' morphology at the bud opening stage of TRV-LoBLH6 plant revealed that, compared to the TRV-control plant, the anthers of the *LoBLH6-*silenced line exhibited uneven yellow-green coloration, with shorter length (Fig. 3b and 3c); additionally, abnormal pollen distribution was observed postdehiscence (Fig. 3d–3g).

To further investigate whether the abnormal anther development leads to pollen abortion, we used 2,3,5- Triphenyltetrazolium chloride (TTC) to stain the pollen. Under the microscope, we observed that the pollen grains of the control line were plump and predominantly dark red after staining, indicating high vitality. In contrast, the *LoBLH6-*silenced strain exhibited a reduced number of pollen grains with a higher proportion of malformed ones. After staining, most of the pollen grains were light red or unstained, suggesting that the silencing of *LoBLH6* led to a decrease in pollen vitality (Fig. 3h and 3i). In pollen germination assay, the *LoBLH6*-silenced lines exhibited a lower pollen germination rate (Fig. 3j and 3k). Scanning electron microscopy (SEM) was employed to investigate the morphological differences in pollen between the *LoBLH6*-silenced lines and the control lines. We observed that pollen from the control lines had a spindle shape with a grid-like deposition pattern on the pollen exine (Fig. 3l), while pollen from the *LoBLH6*-silenced lines was spherical or ellipsoidal, with a densely and irregularly deposited granular exine (Fig. 3m).

Statistical analysis revealed that the pollen quantity in the *LoBLH6*-silenced lines decreased by 36%, pollen viability decreased by 43%, and pollen germination rate decreased by 56%, showing significant differences compared to the control group (Fig. 3n–3p). These results indicated that the silencing of *LoBLH6* could significantly reduce the male fertility in Lilium.

The other silenced lines of *LoBLH6* also exhibited similar phenotypes of pollen abortion (Fig. S5a–S5g), and silencing *LoBLH6* in *Lilium longiflorum* cv. 'White Heaven' also led to a reduction in pollen quantity (Fig. S6a–S6f), providing further evidence for the critical role of *LoBLH6* in regulating pollen development.

### Silencing of *LoBLH6* results in abnormal anther development and GA accumulation

**Figure 4:**
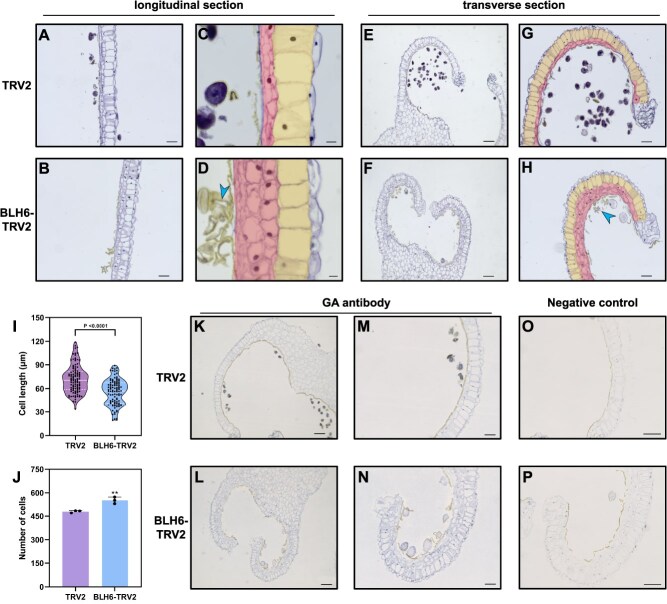
Silencing of *LoBLH6* leads to abnormal development of lily anthers and an elevation in anther GA content. **(*A*)** and **(*B*)** show longitudinal sections of anthers at the early stage of flower bud opening in `Siberia' under a 10× objective for TRV2 and BLH6-TRV2, respectively. TRV indicates tobacco rattle virus, TRV2 indicates the blank control, and BLH6-TRV2 indicates *LoBLH6-*silenced plants. Scale bar = 100 μm. **(*C*)** and **(*D*)** show longitudinal sections of anther wall cells in `Siberia' captured under a 40× objective for TRV2 and BLH6-TRV2, respectively. The middle layer cells are marked in magenta, the endothecial layer cells are marked in yellow, and the blue arrow indicates abnormal pollen. Scale bar = 20 μm. **(*E*)** and **(*F*)** show transverse sections of anthers at the early stage of flower bud opening in `Siberia' under a 5× objective for TRV2 and BLH6-TRV2, respectively. Scale bar = 200 μm. **(*G*)** and **(*H*)** display transverse sections of anther wall cells in `Siberia' captured under a 10× objective for TRV2 and BLH6-TRV2, respectively. Scale bar = 100 μm. **(*I*)** Length of endothecial layer cells in `Siberia' for TRV2 and BLH6-TRV2. The white solid line represents the median, and the white dashed line represents the quartiles (Student's *t*-test, *n* = 100). **(*J*)** Number of longitudinally distributed fiber layer cells in anthers of `Siberia' for TRV2 and BLH6-TRV2 (mean ± SD, *n* = 3, ^**^  *P* < .01, Student's *t*-test). **(*K*)** to **(*P*)** GA immunohistochemistry assay in cross-sectioned anthers for TRV2 and BLH6-TRV2, where the blue color indicates the distribution and concentration of GA. **(*K*)** and **(*L*)** are images captured under a 5× objective, scale bar = 200 μm; **(*M*)** and **(*N*)** are images captured under a 10× objective, scale bar = 100 μm. **(*O*)** and **(*P*)** are images captured under a 10× objective of `Siberia' anther slices in the immunohistochemical experiment without GA antibody as a negative control for GA immunohybridization. Scale bar = 100 μm.

To further elucidate the role of *LoBLH6* on anther development at the cellular level, the organizational structure of mature anthers was observed from the TRV-LoBLH6 silenced plants. From longitudinal sections, it was observed that the cell length of the endothecial layer in *LoBLH6-*silenced line was shorter than the control line, and the middle layer contained more cells (Fig. 4a–4d). In cross-sections, an increased number of middle layer cells was observed in the *LoBLH6*-silenced lines (Fig. 4e–4h, Fig. S5g–S5i). Similar phenotypes were observed in *LoBLH6*-silenced 'White heaven' lines (Fig. S6g and S6h). Moreover, the *LoBLH6*-silenced `Siberia' line contained more deformed pollen (Fig. 4d and 4h). Measurement of fibrous layer cell length and quantity in *LoBLH6*-silenced line and control line revealed a decreased cell length and an increased quantity with *LoBLH6* silencing (Fig. 4i and 4j). There was premature degradation of tapetum and early mitosis of microspores in *LoBLH6-*silenced anthers at a bud length of 7.5 cm (Fig. S5h and S5i). Since cell expansion and cell division are directly or indirectly regulated by GA [29], we speculate that the abnormal development of these anther wall cells and pollen is related to the changes in GA levels [30, 31]. To further verify this hypothesis, we conducted immunohistochemical experiments on the anthers of `Siberia' to analyze the changes in GA content and distribution in the silenced lines and control lines. The results showed that the GA accumulation in the *LoBLH6-*silenced anthers was higher than that in the controls. However, the GA accumulation in the abnormal pollen of the silenced line was lower than that in the normal pollen of the control line (Fig. 3k–3p). We measured the levels of bioactive gibberellins GA_1_ and GA_3_ in mature anthers of the silenced lines using liquid chromatography-mass spectrometry (LC–MS). The results showed that GA_1_ and GA_3_ levels in the *LoBLH6*-silenced anther was significantly higher than in the control line (Fig. S7). Therefore, we guessed that *LoBLH6* might affect anther development by regulating GA accumulation.

### GA homeostasis is crucial for lily anther development

**Figure 5:**
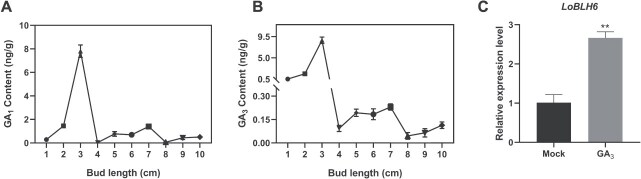
Anther development requires GA homeostasis. **(*A*)** GA_1_ content in `Siberia' anthers at 1–10 cm (mean ± SD, *n* = 3). **(*B*)** GA_3_ content in `Siberia' anthers at 1–10 cm (mean ± SD, *n* = 3). **(*C*)** Expression of *LoBLH6* in `Siberia' anthers after 48 h of GA_3_ treatment (mean ± SD, *n* = 3, ^**^  *P* < .01, Student’s *t*-test). Mock, aqueous ethanol solution without GA_3_ treatment; GA_3_, 50 μg·l^−1^ GA_3_ treatment.

The accumulation of bioactive gibberellins GA_1_ and GA_3_ was examined during anther development in `Siberia'. The bioactive GA content exhibits significant fluctuations during the process of anther development, but it increases and maintains a relatively stable level when the flower bud reaches 5–7 cm in length (Fig. 5a and 5b). During this period, the microspores are undergoing transformation into mature pollen, which is a critical stage of pollen development [27]. Additionally, *LoBLH6* expression is elevated in anthers at the 5-cm flower bud stage, suggesting that *LoBLH6* may be induced by bioactive GA. Silencing of *LoBLH6* results in increased GA levels, disruption of GA homeostasis, and abnormal anther development, suggesting the potential crucial role of GA homeostasis in lily anther development. Further investigations demonstrate that exogenous bioactive GA treatment induces the expression of *LoBLH6* in anthers (Fig. 5c), indicating the involvement of *LoBLH6* in GA-mediated regulation of anther development.

**Figure 6:**
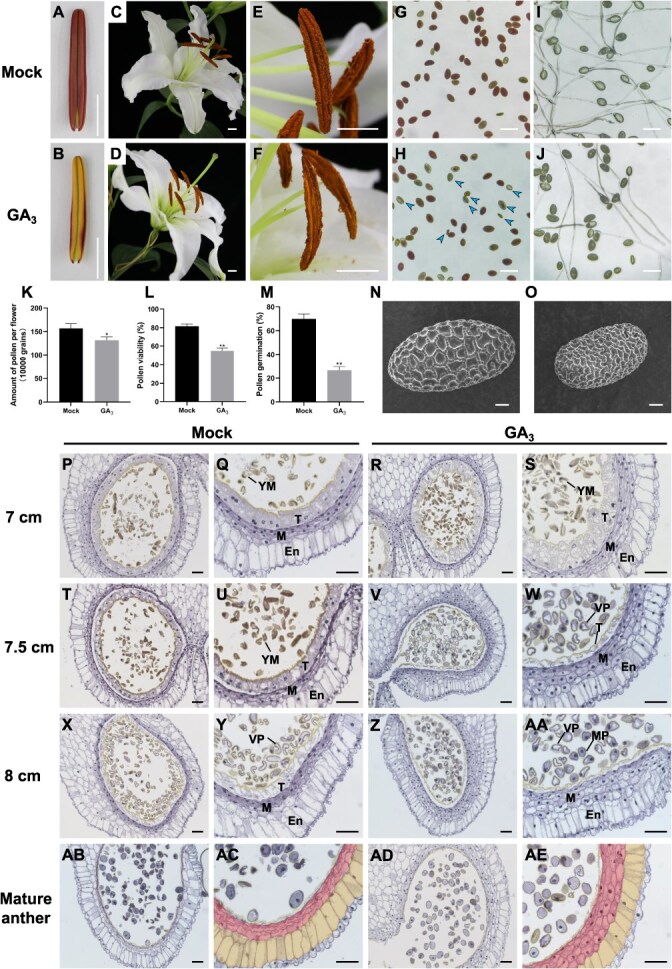
Excessive bioactive GA levels during the midstage of anther development lead to reduced male fertility in lily. **(*A*)** and **(*B*)** show anthers at the early stage of flower bud opening in `Siberia' for TRV2 and BLH6-TRV2, respectively. Scale bar =1 cm. **(*C*)** and **(*D*)** show the appearance of anthers at full bloom in `Siberia' for ethanol treatment (Mock) and 50 μg·l^−1^ GA_3_ treatment (GA_3_), respectively. Scale bar = 1 cm. **(*E*)** and **(*F*)** are magnified views of the anthers in (*A*) and (*B*), Scale bar = 1 cm. **(*G*)** and **(*H*)** show the vitality of mature pollen in `Siberia' for mock group and GA_3_ treatment group, respectively, using TTC staining. Dark red indicates high pollen vitality, while light red or lack of color indicates low or no vitality, the arrows indicate some pollen with no vitality. Scale bar = 100 μm. **(*I*)** and **(*J*)** show the germination status of mature pollen in `Siberia' for mock group and GA_3_ treatment group, respectively. Scale bar = 100 μm. **(*K*)** Pollen quantity statistics for each flower in `Siberia' for GA_3_ treatment group and mock group. Data are the mean ± SD of three biological repeats. Asterisks indicate significant differences (Student's *t*-test, ^*^  *P* < .05). **(*L*)** Vitality statistics of mature pollen in `Siberia' for GA_3_ treatment group and mock group. Data are the mean ± SD of three biological repeats. Asterisks indicate significant differences (Student's *t*-test, ^**^  *P* < .01). **(*M*)** Germination rate statistics of mature pollen in `Siberia' for GA_3_ treatment group and mock group. Data are the mean ± SD of three biological repeats. Asterisks indicate significant differences (Student's *t*-test, ^**^  *P* < .01). **(*N*)** and **(*O*)** show SEM images of mature pollen for mock group and GA_3_ treatment group, respectively. Scale bar = 10 μm. **(*P*)** to **(*AA*)** show transverse sections of anthers at the 7- to 8-cm buds for GA_3_ treatment group and mock group. Scale bar = 100 μm. **(*AB*)** to **(*AE*)** show transverse sections of anthers at the early stage of flower bud opening in `Siberia' for GA_3_ treatment group and mock group. The middle layer cells are marked in magenta, the endothecial layer cells are marked in yellow, Scale bar = 100 μm. Mock, aqueous ethanol solution without GA_3_ treated; GA_3_, 50 μg·l^−1^ GA_3_ treated. En, endothecial cell layer; M, middle layer; T, tapetal layer; YM, young microspore; VP, vacuolated pollen; MP, mature pollen.

To investigate the impact of GA homeostasis disruption on the middle stage of anther development in lilies, we performed exogenous GA_3_ (50 μg·l^−1^) treatment on `Siberia' with a bud length of 4–5 cm. We found that the anther length of the GA-treated anthers was shorter and the color was lighter after maturation compared to the mock group (Fig. 6a and 6b). Postdehiscence, a slight reduction in pollen quantity was observed in GA-treated anthers (Fig. 6c–6f and 6k). TTC staining for pollen viability and pollen germination assays revealed a significant decrease in pollen viability and germination rate following GA treatment (Fig. 6g–6j, Fig. 6l and 6m). Additionally, some mature pollen from the GA-treated group exhibited abnormal pollen exine deposition (Fig. 6n and 6o). To further explore the cellular effects of GA homeostasis disruption on anthers, we conducted paraffin sections on anthers with bud lengths of 7–8 cm and mature anthers after GA treatment. We observed abnormal expansion and premature degradation of the tapetal layer in GA-treated anthers, as well as premature maturation of microspores (Fig. 6p–6aa). The middle layer cells also showed abnormal swelling and delayed degradation, with accumulation persisting even after anther maturation (Fig. 6ab–6ae). These abnormal phenotypes of anther morphology and pollen abortion are highly similar to the phenotype of *LoBLH6*-silenced lines, further validating our hypothesis that LoBLH6 affects anther development by negatively regulating the GA level in anthers.

### LoBLH6 binds to the promoter of *LoGA20ox1* and represses its expression

**Figure 7:**
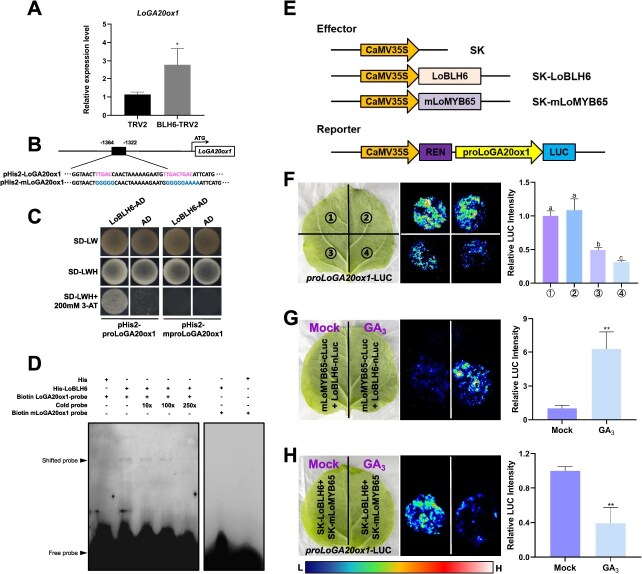
LoBLH6 binds to the promoter of *LoGA20ox1* and represses its expression. **(*A*)** Expression of *LoGA20ox1* in TRV2 and *LoBLH6*-silenced plants. TRV2 indicates the blank control, BLH6-TRV2 indicates the *LoBLH6-*silenced plants. Data are the mean ± SD of three biological repeats. Asterisks indicate significant differences (Student's *t*-test, ^*^  *P* < .05). **(*B*)** Schematic representation of the *LoGA20ox1* promoter. A promoter fragment upstream of the start codon, ranging from 1364 to 1322 bp, was used to construct the pHis2 vector and generate probes for EMSA. The “TAGC” motif in the promoter fragment and the mutated “TAGC” motif were highlighted. **(*C*)** Y1H assay. Y1H analysis was conducted based on yeast growth on selective media lacking Leu, Trp, and His (-LWH), supplemented with 3-amino-1,2,4-triazole (3-AT). The interaction between LoBLH6 and the wild-type *LoGA20ox1* promoter fragment, as well as the mutated promoter fragment with the altered `TGAC' motif, was analyzed. AD, pGADT7 vector. **(*D*)** An EMSA of His-LoBLH6 with the DNA fragment containing the *LoGA20ox1* promoter binding element and the DNA fragment with the mutated binding element. The symbol `+' signifies the presence, whereas `−' signifies the absence. His, 6× histidine tag. **(*E*)** Schematic diagram of effector and reporter structure for the dual-LUC reporter assay. SK, pGreenII62-SK empty vector, used for negative control; LUC, luciferase; REN, Renilla luciferase. **(*F*)** Detection of the *proLoGA20ox1*-LUC signal in *N. benthamiana* leaves and measurement of relative LUC intensity. The representative picture is based on three experiments. **①** SK+ SK+ *proLoGA20ox1*-LUC; **②** SK-mLoMYB65+ SK + *proLoGA20ox1*-LUC; **③** SK-LoBLH6+ SK+ *proLoGA20ox1*-LUC; **④** SK-mLoMYB65+ SK-LoBLH6+ *proLoGA20ox1*-LUC. Data are presented as the mean ± SD of three replicates. The different letters indicated statistically significant differences (Student–Newman–Keuls test, *P* < .05). LUC, luciferase; SK, pGreenII62-SK empty vector, used for negative control. **(*G*)** Detection of LUC signal and measurement of relative LUC intensity in *N. benthamiana* leaves coinfiltrated with mixed bacterial solutions of mLoMYB65-cLuc and LoBLH6-nLuc by different treatments. The representative picture is based on three experiments. Data are presented as the mean ± SD of three replicates, and asterisks indicate significant differences (Student's *t*-test, ^**^  *P* < .01). Mock, aqueous ethanol solution without GA_3_ treated; GA_3_, 50 μg·L^−1^ GA_3_ treated. **(*H*)** Detection of pro*LoGA20ox1*-LUC signal and measurement of relative LUC intensity in *N. benthamiana* leaves coinfiltrated with mixed bacterial solutions of SK-mLoMYB65+ SK-LoBLH6+ pro*LoGA20ox1*-LUC by different treatments. The representative picture is based on three experiments. Data are presented as the mean ± SD of three replicates, and asterisks indicate significant differences (Student's *t*-test, ^**^  *P* < .01). Mock, aqueous ethanol solution without GA_3_ treated; GA_3_, 50 μg·l^−1^ GA_3_ treated.

As a transcriptional repressor, *LoBLH6* expression is induced by GA, suggesting its potential involvement in negative feedback regulation of GA biosynthesis. GA 20-oxidase1 (GA20ox1) is a key enzyme in the GA biosynthetic pathway [32, 33], and the expression of *LoGA20ox1* in anthers of LoBLH6-silenced plants is significantly upregulated (Fig. 7a), indicating that *LoGA20ox1* may be directly regulated by LoBLH6. We isolated the 1869-bp promoter sequence upstream of the start codon of *LoGA20ox1* by the chromosome walking method. After analyzing this promoter sequence, we found that it contains the typical binding sites of the BEL1-like TFs, namely the TGAC motif [34–36]. We isolated the wild-type *LoGA20ox1* promoter fragment, which contains three TGAC motifs, and the mutant version (*mLoGA20ox1*) with altered TGAC motifs, into the pHis2 vector separately (Fig. 7b), and performed yeast one-hybrid (Y1H) assay. The results demonstrated that LoBLH6 bound the TGAC motif of *LoGA20ox1* promoter (Fig. 7c). To further confirm direct binding of LoBLH6 to the *LoGA20ox1* promoter, we generated a 5′ biotin-labeled probe using the aforementioned *LoGA20ox1* promoter fragment and *mLoGA20ox1* promoter fragment (Fig. 7b). An electrophoretic mobility shift assay (EMSA) showed that LoBLH6 specifically bound the *LoGA20ox1* probe *in vitro* but not the mutant probe (Fig. 7d).

**Figure 8:**
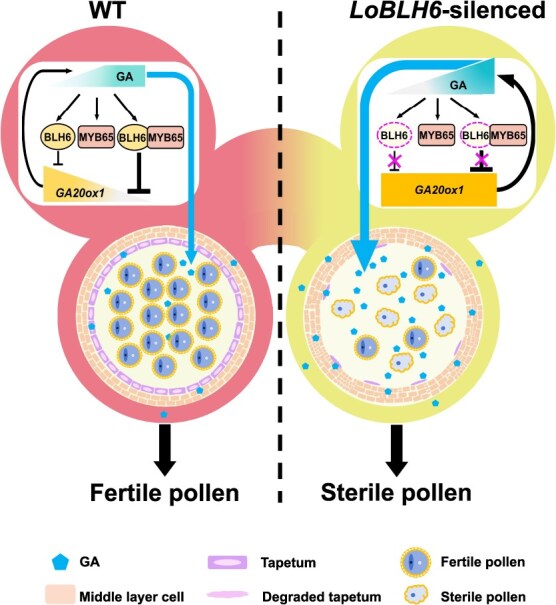
Working model explaining how the LoBLH6-LoMYB65 module feedback regulates GA-mediated pollen development. When the GA level increases, the expression of *LoBLH6* and *LoMYB65* is induced. BLH6 can independently inhibit the expression of *LoGA20ox1*, and its interaction with LoMYB65 enhanced the inhibitory effect on *LoGA20ox1* expression. Moreover, higher concentrations of GA further promote this interaction, leading to the suppression of GA biosynthesis and maintaining GA contents at a stable level, ensuring normal pollen development occurs. When *Lo*BLH6 was silenced, the LoBLH6-LoMYB65 module in anthers is disrupted. The expression of *LoGA20ox1* is no longer repressed by LoBLH6, disrupting the negative feedback regulation of GA synthesis. The elevated GA levels result in premature PCD in the tapetum, abnormal development of anther wall cells, and ultimately the production of aborted pollen. The pointed-end arrows indicate positive effects, the blunt-end arrows indicate negative effects, and the bold line indicate enhanced functions.

Dual-luciferase (LUC) assay demonstrated that the accumulation of LoBLH6 repressed the activity of the *LoGA20ox1* promoter, and this repression was more pronounced when LoMYB65 existed (Fig. 7e and 7f). The LCI assay showed that GA treatment enhanced the intensity of LoBLH6–LoMYB65 interaction (Fig. 7g) and led to a further suppression of *LoGA20ox1* promoter activity (Fig. 7h). In summary, LoBLH6 could bind the promoter of *LoGA20ox1* and repress its expression, and GA enhanced this repression by promoting the interaction between LoBLH6 and LoMYB65.

## Discussion

In various plants, such as Arabidopsis, rice, and barley, extensive research has demonstrated the core role of GAMYBs in anther development [11, 37–39]. LoMYB65 has been confirmed in our previous study to be essential for lily anther development [3]. Here, we found that LoBLH6 exhibited a similar expression pattern and spatial expression characteristics with LoMYB65 (Fig. 2); in addition, LoBLH6 directly interacted with LoMYB65 (Fig. 1a–1d). Silencing *LoBLH6* in both `Siberia' and 'White Heaven' resulted in conspicuous phenotypes of abnormal anther development and pollen sterility. These findings suggest that LoBLH6 play a required role in anther development.

BLHs always interact with the members of KNOX family, forming heterodimers to fully exert their biological functions. Unlike the conserved interactions between BLHs and KNOXs, our study revealed that although LoBLH6 interacted with LoMYB65, it did not interact with the GAMYB member LoMYB33 (Fig. S8). This suggests that LoMYB65 may possess novel functions beyond the conserved roles of GAMYBs. Previous research has reported that MYB65 acts as a downstream component in the GA signaling pathway, where GA promotes *GAMYB* expression by relieving the inhibitory effect of DELLA proteins. However, our results indicated that MYB65 also functioned as a regulator in GA synthesis, which highlighted the multifaceted role of MYB65 in both the GA signaling cascade and its synthesis.

The selective interactions between BLHs and different proteins influence the subcellular localization and target affinities of heterodimers, meaning that distinct heterodimers may exert different transcriptional regulatory functions on the target promoter sequences [40–42]. In Arabidopsis, separate AtBLH3 functions as a transcriptional activator, but when it interacts with AtOFP1, the transactivation ability is inhibited [43]. Conversely, the heterodimerization of BLH1 and KNAT3 further promotes the transcription of *ABI3* [21]. In our study, we found that LoBLH6 exerts a stronger transcriptional inhibition on *LoGA20ox1* after forming a complex with LoMYB65 (Fig. 7f).

The anthers undergo vigorous GA biosynthesis during development [44–46], and simultaneously, bioactive GAs are inactivated to maintain GA homeostasis. GA3oxs and GA20oxs are the major synthetic enzymes for bioactive GAs, while GA2oxs are the primary inactivating enzymes for bioactive GAs [47, 48]. GA20ox is a limiting factor for the accumulation of bioactive GAs in anther development [49, 50]. The GA20ox members in plants typically consists of a series of paralogous genes with different expression characteristics [51]. In developed anthers of Arabidopsis and tomato, *GA20ox1* is highly expressed and plays a major role among *GA20oxs* [52–54], which was also found in lily anthers by transcriptome analysis (Table S5). The hormone homeostasis is essential for normal plant development, as well as anthers. Excessive or insufficient GA accumulation in anthers causes decreased fertility [49, 55]. Previous studies have shown that an increase in GA content inhibits the expression of *GA20oxs* and *GA3oxs* and promotes the expression of *GA2oxs*, limiting the excessive elevation of GA content [48]. When the flower bud grows from 5 to 7 cm, crucial processes occur in the anther, including the transformation of mononuclear microspores into mature pollen and the PCD of the tapetal layer [27]. At this stage, there is a stable accumulation of GA. In this study, we discovered a rapid GA accumulation in anthers when the flower bud grew from 4 to 5 cm (Fig. 5), which might subsequently activated the expression of *LoBLH6* and *LoMYB65* (Fig. 2b, 2c and Fig. 5c) [3]. LoBLH6 repressed the expression of *LoGA20ox1.* In addition, LoBLH6 also interacted with LoMYB65, and GA treatment promoted this interaction, which further elevated the repression effect (Fig. 7f–7h). In the anther, this `multilayered safeguard' inhibitory mechanism centered around LoBLH6 progressively restricts GA synthesis, aiming to maintain the GA homeostasis during anther development. Nevertheless, we found no apparent correlation between GA fluctuation and *LoBLH6* expression in early pollen development and pollen maturation. This suggests that anther development at different stages may be governed by different gene regulatory networks, and the specific molecular mechanisms need further investigation.

The tapetum is crucial for pollen development as it provides multifaceted support for pollen development and ultimately degrades to provide nutrients for the continued development of microspores [11, 56, 57]. Therefore, premature or delayed degradation of the tapetum severely impacts fertility [58–60]. Tapetal layer PCD is induced by GA, with an elevation in GA levels causing premature tapetal layer PCD [52]. In the *LoBLH6*-silenced anther, an increase in GA content was observed (Fig. 4k–4p), coinciding with an early onset of tapetal layer PCD and an acceleration in microspore development (Fig. S5f and S5g), which was highly similar to the phenotype of anther treated with GA_3_. Application of GA_3_ to wheat results in reduced anther size and lighter color [39]. Similarly, we observed a reduction in anther size and a shift in color from deep purple-red in the normal anthers to yellow-green in the *LoBLH6*-silenced line and GA_3_-treated lily anthers (Fig. 3b and 3c, 6a and 6b). We speculate that this phenomenon is related to the inhibitory effect of high concentrations of GA on anthocyanin accumulation [61, 62].

During anther development, the middle layer cells gradually degrade and disappear [63]. In the *LoBLH6*-silenced anthers, a significant number of undegraded middle layer cells were observed (Fig. 4a–4h). In normal lily anthers, the tapetal cells are typically a single layer, while the middle layer cells divide into 4–5 layers and then gradually degrade during anther development [27, 63]. Previous studies have indicated that an increase in GA levels induces the production of cytokinins in anthers [58], and cytokinins are known to induce cell division and expansion [64]. We speculate that during the midstage of anther development, the middle layer cells of *LoBLH6*-silenced lines are induced by GA and cytokinin to expand and divide. Meanwhile, the tapetal cells have no potential for induced division, resulting in abnormal expansion only. At the same time, PCD is induced by GA, leading to premature degradation of the tapetal layer during the midstage of anther development, while there is a large number of undegraded middle layer cells at the late stage of anther development. Thus, in the anther of *LoBLH6*-silenced line at the 7.5-cm stage, we observed abnormally developed anther wall cells and microspores, as well as sterile pollen present in the dehisced anther.

BLHs are known to be involved in regulating structural genes in the phenylpropanoid pathway [65], and phenylpropanoids are essential components of sporopollenin [66, 67]. Normally, lily pollen exine exhibited a grid-like pattern (Fig. 3l and 6n) [66], but we observed that the pollen exine in the *LoBLH6*-silenced plants appeared densely packed with irregular granules (Fig. 3m), and compared to GA_3_-treated lines, there were more pollen grains with abnormal pollen wall development in the silenced lines (Fig. 3i and 6h). Therefore, we speculate that besides inducing imbalances in GA synthesis, silencing *LoBLH6* may also adversely affect pollen development by interfering with sporopollenin synthesis.

In conclusion, we have identified an unreported role for the BLH member LoBLH6 in regulating anther development. LoBLH6 is induced by GA and directly binds to the promoter of *LoGA20ox1* to suppress its expression. LoBLH6 also interacts with LoMYB65 to form a complex with enhanced transcriptional repression activity and GA promotes the interaction and transcriptional repression activity of this complex on *LoGA20ox1*, thereby maintaining GA homeostasis in the anther. This prevents excessively high GA levels from affecting the development of anther wall cells, ensuring the production of fertile pollen (Fig. 8). Our study not only reveals a new regulatory network between the GA synthesis pathway and signaling pathway but also provides a novel gene for reference in the creation of male-sterile varieties in horticultural plants and crops.

## Materials and methods

### Plant materials and growth conditions

The lily 'White heaven' tissue-cultured seedlings were cultured on solid Murashige & Skoog (MS) medium under the environmental conditions of 22°C with 16 h of light and 8 h of darkness per day. When lilies reach the three- to four-leaf stage, they were transplanted into a growth substrate and further cultivated in a growth chamber with conditions set at 22°C and 16 h of light and 8 h of darkness per day. The `Siberia' bulbs were purchased from Hongyue Horticultural Company (China) and, after dormancy release in cold storage, they were cultivated in a greenhouse with a daytime temperature of 22°C and nighttime temperature of 16°C. *Arabidopsis thaliana* (Ecotype Columbia) and *N. benthamiana* (tobacco) were grown in a growth chamber under conditions of 22°C with 16 h of light and 8 h of darkness per day.

The GA_3_ treatment 50 μg·l^−1^ was conducted using cut flowers of `Siberia' with flower buds measuring 5 cm. The cut flower stems were immersed in the GA_3_ solution and cultivated under 12 000 Lux light intensity. GA_3_ is dissolved in ethanol to prepare mother liquor, and the final concentration is 50 μg·l^−1^ by adding water. A water–ethanol solution without GA_3_ was used as a control.

### Isolation of *LoBLH6,* phylogenetic tree construction, and amino acid sequence analysis

Total RNA was extracted from anthers of `Siberia' with a bud length of 5 cm, and after digestion of genomic DNA, it was reverse-transcribed into cDNA using M-MLV reverse transcriptase (Vazyme, Nanjing, China). Primers for *LoBLH6* were designed for PCR cloning based on the lily anther RNA-seq database (Table S1).

The amino acid sequence of LoBLH6 was subjected to homology comparison using DNAMAN 6.0, and the phylogenetic tree was constructed using MEGA-X with the neighbor-joining method [68]. The bioinformatics data were downloaded from the National Center for Biotechnology Information (NCBI) (https://www.ncbi.nlm.nih.gov/) and TAIR database (https://www.arabidopsis.org/index.jsp).

### Subcellular localization analysis

The *GFP* sequences were separately fused to the 5′ and 3′ ends of *LoBLH6*, and then inserted into the pCAMBIA1300 vector for transformation into *Agrobacterium* GV3101 competent cell. The RFP-NLS vector was used for nuclear marker, while the pCAMBIA1300-GFP vector served as a positive control. *Nicotiana benthamiana* leaves were infiltrated with the *Agrobacterium* solution, and images were captured using a Zeiss LSM800 laser scanning confocal microscope (Germany). The primers used are detailed in Table S3.

### Pull-down assay

The *LoBLH6* was cloned into the pGEX4T-1 vector to produce the GST-LoBLH6 fusion protein. The *LoMYB65* was cloned into the pMAL-p5x vector (GE Healthcare, USA) to produce the MBP-LoMYB65 fusion protein. The pGEX4T-1 empty vector was used to produce the GST protein. The above-mentioned vectors were transformed into *Escherichia coli* BL21, and protein expression was induced using Isopropyl-β-D-thiogalactoside (IPTG). GST or GST-LoBLH6 was mixed with MBP-LoMYB65 protein solution, respectively, and incubated with GST beads at 4°C for 3 h. After purification, protein immunoblot analysis was conducted [69]. The primers used are listed in Table S3.

### LCI and dual-LUC reporter assay

For the LCI assay, the *LoBLH6* and *mLoMYB65* were individually inserted into the pCAMBIA1300-nLuc and pCAMBIA1300-cLuc vectors and transformed into *Agrobacterium tumefaciens* GV3101 competent cell [70]. For the Dual-LUC reporter assay, the *LoBLH6* and *mLoMYB65* were separately inserted into the pGreenII62-SK (SK-II) vector, and the promoter *proLoGA20ox1* (1869 bp) was inserted into the pGreenII0800-LUC vector [71]. These constructs were transformed into *A. tumefaciens* GV3101 (pSoup). *A. tumefaciens* were resuspended in infiltration buffer (10 mM MgCl_2_, 200 μM acetosyringone (AS), and 10 mM MES Monohydrate, pH 5.8) and the suspensions of different experimental combinations were mixed and kept in the dark at 22°C for 3 h. *Nicotiana benthamiana* leaves were gently punctured with a needle, and the *Agrobacterium* suspension was slowly infiltrated into the leaves using a syringe without a needle, ensuring an equal infiltration area for different experimental combinations on the same leaf. The infiltrated tobacco plants were kept in the dark for 1 day, followed by a 2-day light incubation. For combinations requiring treatment with GA_3_, we applied 50 μg·l^−1^ GA_3_ to the leaves 2 h prior to observing bioluminescence, using an aqueous ethanol solution without GA_3_ as a negative control. *Nicotiana benthamiana* leaves were sprayed with D-Luciferin, Potassium Salt (YEASEN, Cat.40902ES02, China), and luminescence was detected using Tannon bioluminescence imaging system (China). Luminescence intensity was represented using a pseudocolor, and LUN/REN values were determined. The primer sequences are provided in Table S3.

### BiFC

The interaction regions of LoBLH6 (B3) and LoMYB65 (M2) were separately inserted into the pSPYNE173 and pSPYCE(M) vectors and transformed into *A. tumefaciens* strain GV3101 competent cell [72]. *Nicotiana benthamiana* leaves were infiltrated with the transformed *Agrobacterium.* After infiltration, *N. benthamiana* plants were kept in darkness for 1 day followed by 2 days of normal light conditions. Fluorescence was excited using a 488-nm laser under a laser scanning confocal microscope for observation and imaging [73]. Primer sequences are provided in Table S3.

### Y1H assay

The ORF of *LoBLH6* and the promoter fragment of *LoGA20ox1* were constructed onto the pGADT7 vector (AD, Clontech, Japan) and pHis2 vector (Clontech, Japan), respectively. The corresponding AD and pHis2 vectors were cotransformed into Y187 yeast-competent cells. After culturing for 3 days on SD medium lacking Leu and Trp, successful transformants were selected and spotted onto Leu-Trp-His-deficient SD medium containing 200 mM 3-AT (3-amino-1,2,4-triazole). The binding of the transcription factor to the DNA fragment was determined based on the growth status of the yeast.

### Y2H assay

The ORFs of *LoBLH6* and *LoMYB65* were individually inserted into pGADT7 (AD, Clontech, Japan) and pGBKT7 (BD, Clontech, Japan). Empty AD and BD vectors served as negative controls. The corresponding AD and BD plasmids were cotransformed into yeast strain AH109 competent cell, and the potential interaction was determined by observing the growth of yeast spots on SD medium lacking Leu-Trp-His-Ade. Primer sequences were listed in Table S3.

### Transcriptional activity analysis

The full-length LoBLH6, VP16 activating sequence, and the fused sequence of LoBLH6 with VP16 were inserted into the BD vector (pGBKT7, Clontech, CA, USA). GAL4 and BD empty vectors served as positive and negative controls. The recombinant plasmids were transformed into the yeast strain AH109 competent cell, and yeast growth was selected on SD medium lacking Trp for 3 days at 30°C in the dark. Successfully transformed yeast colonies were further incubated on SD medium lacking Trp and His (Histidine), supplemented with 3-AT at 30°C for 3 days. Transcriptional activation activity was assessed using β-galactosidase activity. The transcriptional activity of LoBLH6 *in vivo* followed the method outlined by Wang et al [74]. Primer sequences were listed in Table S3. We inserted the *LoBLH6* into the pBD and pBD-VP16 vectors to construct pBD-LoBLH6 and pBD-LoBLH6-VP16 vectors as effectors. Effectors and reporter were transformed into *Agrobacterium* GV3101 respectively, and *N. benthamiana* leaves were coinfected. Luminescence images were taken in a bioluminescence imaging system (Tannon, China), and relative enzyme activity measured using Dual Luciferase Reporter Assay Kit (Vazyme, Cat.DL101–01, China).

### Gene expression assay

Total RNA from the plant tissues was extracted using the Plant RNA Extraction Kit (Accurate Biotechnology, Cat. AG21019, Hunan, China). The extracted RNA was diluted to a concentration of ~200 ng·μl^−1^ with RNase-free ddH_2_O. Reverse transcription was performed using the HiScript III RT SuperMix for qPCR kit (Vazyme, Cat. R323–01, China). qPCR was conducted using the SYBR Green method (Accurate Biotechnology, Cat. AG11735, China), and the relative gene expression level was calculated using the 2^-ΔΔCt^ method. Primer sequences were provided in Table S2.

### Promoter isolation

We referred to the lily genome information [75] and then isolated the promoter of *LoBLH6* and *LoGA20ox1* using the Hi-tail PCR method [76]. The fragments of 1590 bp upstream of the *LoBLH6* start codon and 1869 bp upstream of the *LoGA20ox1* start codon were identified.

### Quantification of GAs

GA quantitative analysis was completed by RuiYuan Biotechnology (Nanjing, China) using the UPLC-MS/MS method, and isotope dilution method was used for quantitative analysis. Briefly, at each developmental stage, three samples of buds, with more than three buds in each sample, were collected. The anthers from the buds were removed, ground with liquid nitrogen, and extracted using acetonitrile.

Liquid chromatography conditions: poroshell 120 SB-C18 2.1 × 150, 2.7 μm reversed-phase column; 30°C column temperature. Mobile phase A: methanol solution containing 0.1% formic acid, Mobile phase B: water solution containing 0.1% formic acid. The injection volume is 2 μl.

Mass spectrometry parameter settings: ESI negative and positive ion modes were monitored separately; Scan type: MRM; Curtain gas: 15 psi; Spray voltage: +4500 V, −4000 V; Nebulizer gas pressure: 65 psi; Auxiliary gas pressure: 70 psi; Nebulizer temperature: 400°C.

### VIGS

The VIGS method followed the protocol described by Liu et al and Wu et al [3, 77]. A specific 300-bp fragment of *LoBLH6* was inserted into the pTRV2 vector. The pTRV2 plasmid, pTRV2-LoBLH6 plasmid, and pTRV1 vector were transformed into *A. tumefaciens* GV3101 competent cell, separately. Fresh *Agrobacterium* cultures were resuspended in infiltration buffer and adjusted to OD_600_ = 1.0. A mixture of *Agrobacterium* suspension with a ratio of TRV1:TRV2 = 1:1 or TRV1:TRV2-LoBLH6 = 1:1 was prepared and incubated in the dark for 3 h. The infection was carried out by injecting the leaf backside at the top of the plant with the bacterial solution. After 1 day dark and subsequent recovery in light, plants were reinfected every 7 days until flowering, and RNA was extracted from anthers to assess the silencing effect.

### Plant phenotypic observation

To assess pollen viability, fresh pollen was scattered in TTC buffer (Coolaber, Cat. SL7144, China), gently mixed by pipetting, and incubated at 35°C in the dark for 15 min. The pollen was immediately observed and counted under a microscope; viable pollen was stained red.

For pollen germination rate determination, fresh pollen was resuspended in liquid pollen germination medium (100 g·l^−1^ sucrose, 20 mg·l^−1^ H_3_BO_3_, in H_2_O), gently mixed, and dropped onto solid pollen germination medium (100 g·l^−1^ sucrose, 20 mg·l^−1^ H_3_BO_3_, 10 g·l^−1^ agar, in H_2_O). After incubation in a 26°C environment with light for 6 h, germination rates were recorded by capturing images under a microscope.

Pollen quantity estimation followed the method outlined by Liu et al [3], after the anthers were dried in EP tubes at 60°C, 8 ml of 200 g·l^−1^ (NaPO_3_)_6_ solution was added and shaken on a vortex mixer to completely release the pollen and mix evenly. Pipette six drops of 2.5 μl of suspension, count the amount of pollen in each drop, and calculate the average. Pollen amount = number of anthers × number of pollen grains in droplets × 3200.

In order to maintain the natural form of pollen to the greatest extent, we collected fresh pollen from lily anthers within 3 days of dehiscence, sprinkled a small amount of pollen onto conductive tape, and observed it under SEM (Hitachi, FlexSEM1000, Japan).

### Tissue sections, RNA *in situ* hybridization, and GA immunohistochemistry assay

Plant tissues were placed in prechilled 50% FAA fixative (formaldehyde-acetic acid-ethanol buffer; 50% EtOH, 5% HAc, and 3.7% HCHO) and vacuum-infiltrated twice on ice for 15 min each. Afterward, the fixative was replaced, and the samples were fixed for 16 h. Dehydration using ethanol solution with increasing concentration (50%, 70%, 85%, 90%, 95%, 100%). Subsequently, a series of xylene–ethanol solutions (25%, 50%, 75%, 100%) were used to gradually replace ethanol with pure xylene. Samples were further substituted with paraffin after which they were embedded in pure solid paraffin. The samples were sectioned into 8-μm-thick ribbons using a microtome, and these ribbons were flattened in a 37°C water bath, adhered to RNase-free glass slides treated with poly-L-lysine, and used for hematoxylin staining, RNA *in situ* hybridization, and GA immunohistochemistry.

The RNA *in situ* hybridization method referred to Samach et al [78]. Specific segments of *LoBLH6* and *LoMYB65* were PCR-amplified, and digoxigenin-labeled sense and antisense probes were produced using the Digoxin RNA Labeling Kit (SP6/T7) (Roche, Cat. No. 11175025910, Germany). The labeled probes were hybridized using Anti-Digoxigenin-AP, Fab fragments (Roche, Cat. No. 11093274910, Germany), and the color reaction was performed using NBT/BCIP Stock Solution (Roche, Cat. No. 11681451001, Germany). All experimental materials were treated to eliminate RNases. Primer sequences can be found in Table S4.

The GA immunohistochemistry assay method followed Marquez-Lopez et al [79], using Rabbit Anti-Gibberellins antibody (Bioss, Cat. bs-4606R, China) as the primary antibody and AP-conjugated Goat Anti-Rabbit IgG (Sangon Biotech, Cat. No. D110072, China) as the secondary antibody. The color reaction was performed using NBT/ BCIP Stock Solution (Roche, Cat. No. 11681451001, Germany).

### EMSA

The *LoBLH6* was fused into the pCOLD-I vector, and His-LoBLH6 fusion protein and His-tagged protein expressed by the pCOLD-I empty vector were produced in *E. coli* BL21. Induction of expression was achieved with 0.5 mM IPTG (Sigma) at 15°C for 16–20 h. Bacterial cells were collected by centrifugation and lysed with an ultrasonic disruptor. His-tagged proteins were purified using the His-tag Protein Purification Kit (Beyotime, Cat: P2226, China) for EMSA experiments. The 5′-biotin-labeled probes were synthesized by Zixi Biotech (Suzhou, China), and EMSA experiments were conducted using the LightShift Chemiluminescent EMSA Kit (Thermo Scientific, Cat: 20148, USA).

### Statistical analyses

Graphs and statistical analyses were generated using GraphPad Prism version 8.0 and IBM SPSS Statistics 19. The Student's *t*-test was employed for mean comparisons between two independent samples, while the Student–Newman–Keuls test was used for comparisons among means of multiple samples. Significance was considered at a level of *P* < .05, indicating the presence of significant differences.

## Supplementary Material

Web_Material_uhae339

## Data Availability

The data and figures in this study can be found in the article and its supporting materials.
