# Supplementary material for: LoBLH6 interacts with LoMYB65 to regulate anther development through feedback regulation of gibberellin synthesis in lily
Source: Hortic Res. 2024 Dec 4;12(3):uhae339. doi: 10.1093/hr/uhae339 (PMC11886847; doi:10.1093/hr/uhae339)
Supplement: Web_Material_uhae339 [file web_material_uhae339.zip › Supplementary File 2024-11-02.docx]

**Supplementary data**

LoBLH6 interacts with LoMYB65 to regulate anther development through feedback regulation of gibberellin synthesis in lily

Junpeng Yu^a, b, 1^, Ze Wu^a, b, 1^, Xinyue Liu^a, b, 1^, Qianqian Fang^a, b^, Xue Pan^a, b^, Sujuan Xu^a, b^, Man He^a, b^, Jinxing Lin^c, d^, Nianjun Teng^a, b, *^

a Key Laboratory of Landscaping, Ministry of Agriculture and Rural Affairs, Key Laboratory of Biology of Ornamental Plants in East China, National Forestry and Grassland Administration, College of Horticulture, Nanjing Agricultural University, Nanjing 210095, China

b Lily Science and Technology Backyard Qixia of Jiangsu/Jiangsu Graduate Workstation, Nanjing 210043, China

c Beijing Advanced Innovation Center for Tree Breeding by Molecular Design, Beijing Forestry University, Beijing 100083, China

d College of Biological Sciences and Biotechnology, Beijing Forestry University, Beijing 100083, China

^*^ Corresponding author: Nianjun Teng.

^1^ J. Y., Z. W. and X. L. contributed equally to this work.

**Email:**  [njteng@njau.edu.cn](mailto:njteng@njau.edu.cn)


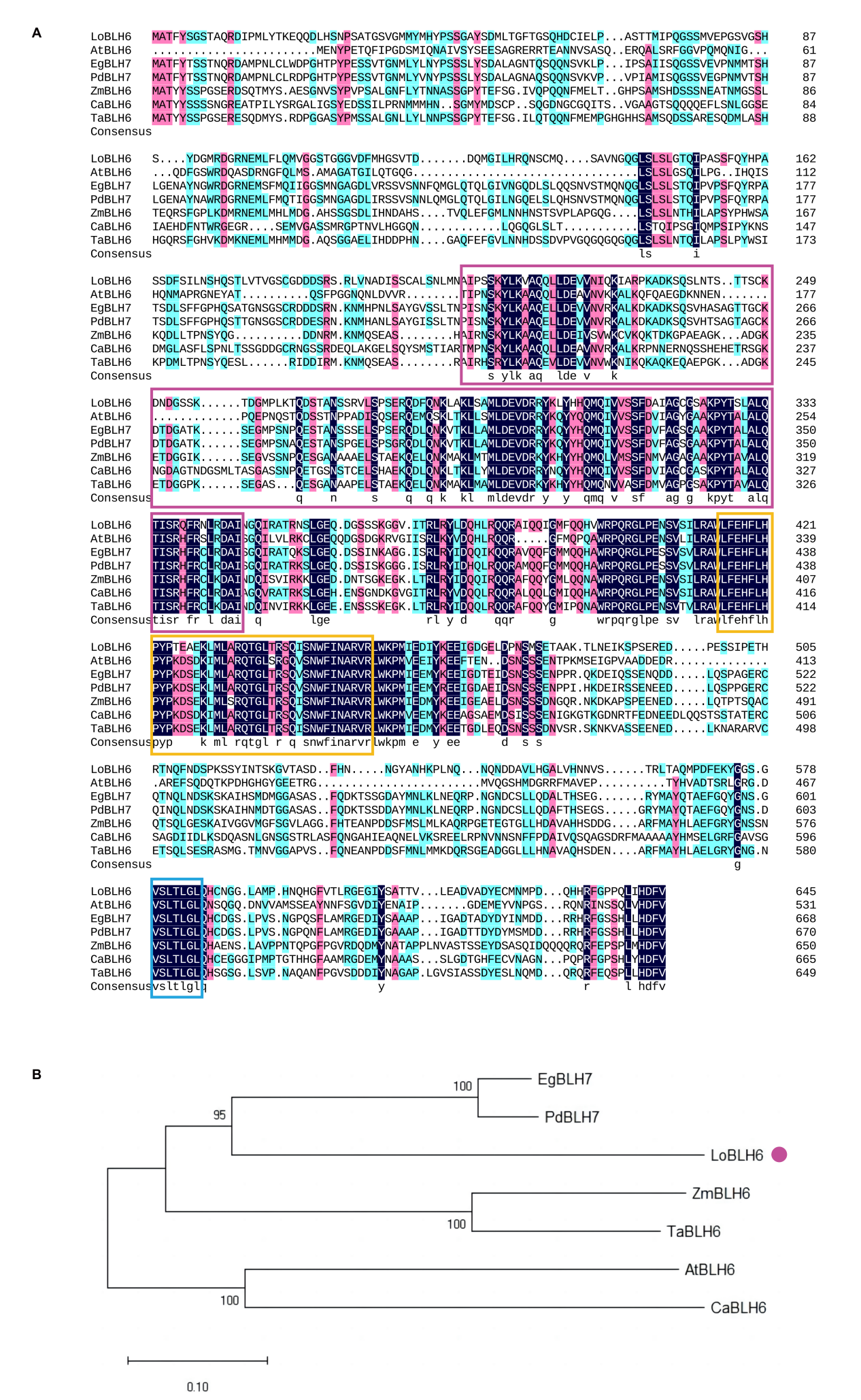


**Fig. S1**. **Amino sequence and phylogenetic analysis of LoBLH6.**

**(*A*)** Alignment of the amino acid sequences of LoBLH6 with homologous proteins from other plant species. Amino sequences were taken from *Lilium* spp. (LoBLH6), *Arabidopsis thaliana*(AtBLH6, NP_001119116.1), *Elaeis guineensis* (EgGAMYB, XP_010932911.1), *Phoenix dactylifera* (PdBLH7, XP_038987957.1), *Zea mays* (ZmBLH6, ACG29257.1), *Cucurbita argyrosperma* (CaBLH6, KAG7035068.1), *Triticum aestivum* (TaBLH7, XP_044379140.1). The POX domain sequence is in the magenta box, the Homeobox domain (HD) is in the orange box, and the ‘VSLTLGL’ region is in blue box. **(*B*)** The amino acid sequence of LoBLH6 and the phylogenetic tree constructed with homologous proteins from other plant species. The evolutionary tree was constructed using the neighbor-joining method in MEGA-X, with n = 500 bootstrap replicates. The numbers on the branches indicate bootstrap percentages, and the scale bar indicates the nucleotide substitutions per site.


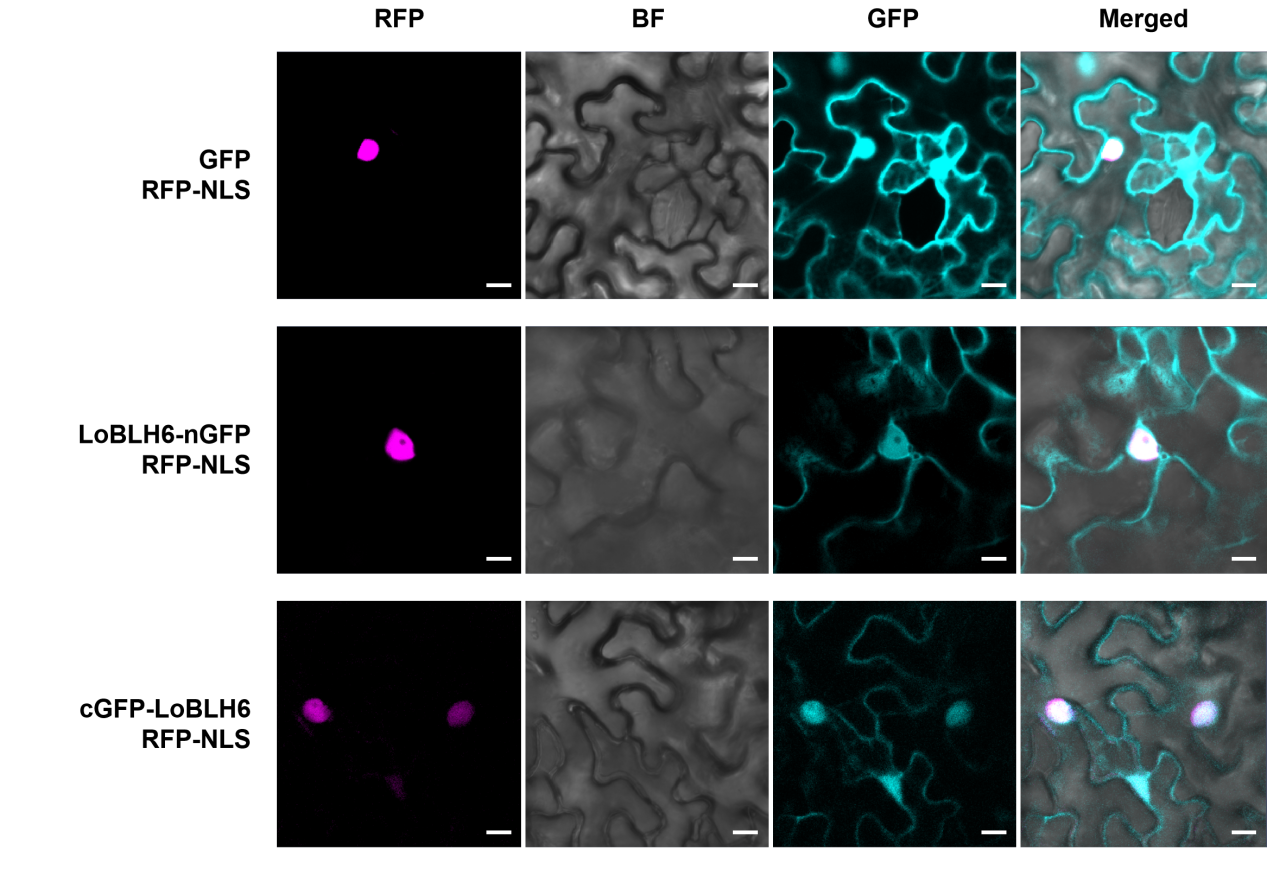
**Fig. S2. Subcellular localization analysis of LoBLH6.**

Detection of fluorescence signals in *N. benthamiana* leaf cells cotransfected with LoBLH6–GFP and the nuclear marker RFP-NLS. The empty GFP vector was the negative control. GFP, green fluorescent protein; RFP, red fluorescent protein; NLS, nuclear localization signal. LoBLH6-nGFP refers to the fusion protein of LoBLH6 with a GFP tag at the N-terminus; cGFP-LoBLH6 refers to the fusion protein of LoBLH6 with a GFP tag at the C-terminus. Scale bar = 10 μm.


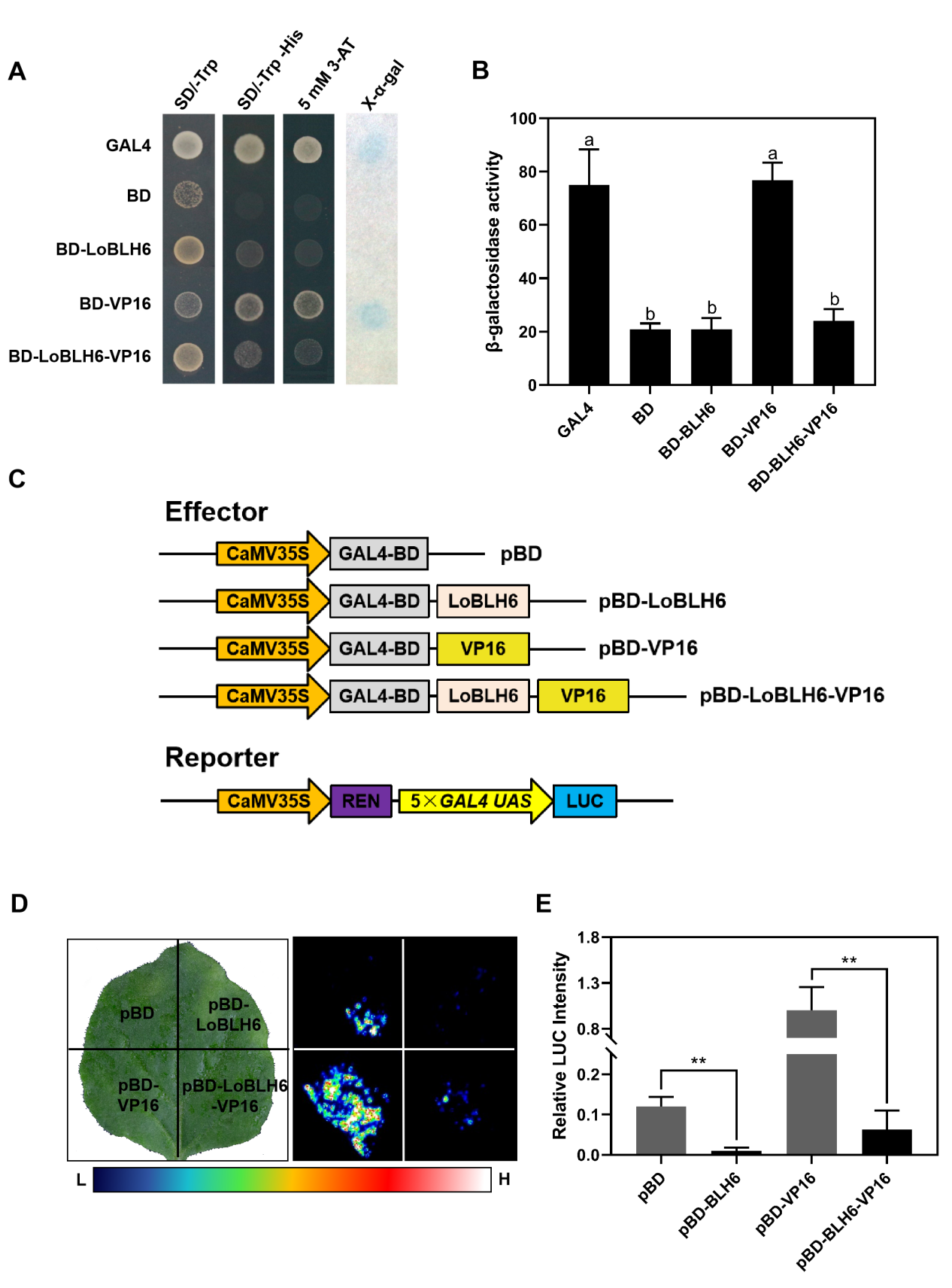


**Fig. S3.** **Analysis of the transcriptional activity of LoBLH6.**

**(*A*)** Transcriptional activity analysis of LoBLH6 in yeast cells. Transcriptional activity was assessed by the growth of yeast on -Trp -His dropout medium and -Trp -His dropout medium supplemented with 3-Amino-1,2,4-triazole (3-AT). GAL4 served as the positive control, BD as the negative control, and VP16 as a transcriptional activator. **(*B*)** Measurement of β-galactosidase activity in yeast cells. Enzyme activity was determined colorimetrically using 2-nitrophenyl β-D-galactopyranoside (ONPG) as the substrate. Data are presented as the mean ± SD of three replicates, with different letters indicating statistically significant difference (Student–Newman–Keuls test, *P* < 0.05). **(*C*)** Schematic representation of the structures of the effector and reporter used in the dual-luciferase assay. **(*D*)** Transactivation assay. Luminescence intensity in *N. benthamiana* leaves reflects the strength of the transactivation effect. The pseudocolor bar represents the range of luminescence intensity in the image. **(*E*)** Test of relative LUC intensity. Bars represent the mean ± SD of five replicates. Asterisks indicate significant differences (Student’ s *t*-test, ** *P* < 0.01).


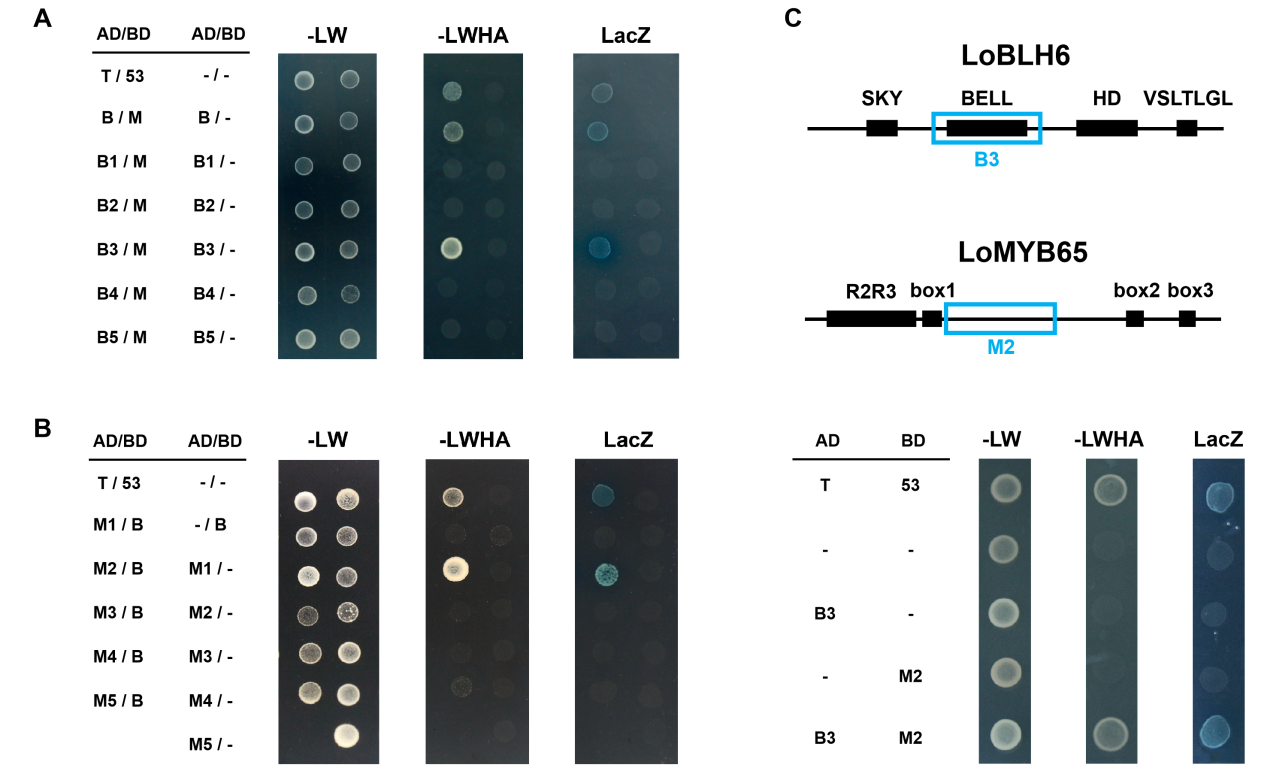


**Fig. S4. LoBLH6 interacts with the region between box1 and box2 of LoMYB65 through its BELL domain.**

Yeast strains co-transformed with the pGADT7-T and pGBKT7-53 vectors served as the positive control group. Protein-protein interactions were assessed based on yeast growth on selective media lacking Leu, Trp, His, and Ade (-LWHA). **(*A*)** Interaction between the BELL domain of LoBLH6 and LoMYB65. B, LoBLH6; M, LoMYB65; B1, fragment 1 of LoBLH6 (1-152 aa); B2, fragment 2 of LoBLH6 (153-236 aa); B3, fragment 3 of LoBLH6 (237-392 aa); B4, fragment 4 of LoBLH6 (393-476 aa); B5, fragment 5 of LoBLH6 (477-646 aa); AD, pGADT7; BD, pGBKT7. **(*B*)** Interaction between the region between box1 and box2 of LoMYB65 and LoBLH6. B, LoBLH6; M, LoMYB65; M1, fragment 1 of LoMYB65 (1-166 aa); M2, fragment 2 of LoMYB65 (167-439 aa); M3, fragment 3 of LoMYB65 (440-488 aa); M4, fragment 4 of LoMYB65 (489-555 aa); M5, fragment 5 of LoMYB65 (556-713 aa); AD, pGADT7; BD, pGBKT7. **(*C*)** Interaction between the BELL domain of LoBLH6 and the region between box1 and box2 of LoMYB65. The blue boxes indicate the positions of the B3 and M2 domains in LoBLH6 and LoMYB65, respectively. B3, fragment 3 of LoBLH6 (237-392 aa); M2, fragment 2 of LoMYB65 (167-439 aa); AD, pGADT7; BD, pGBKT7.


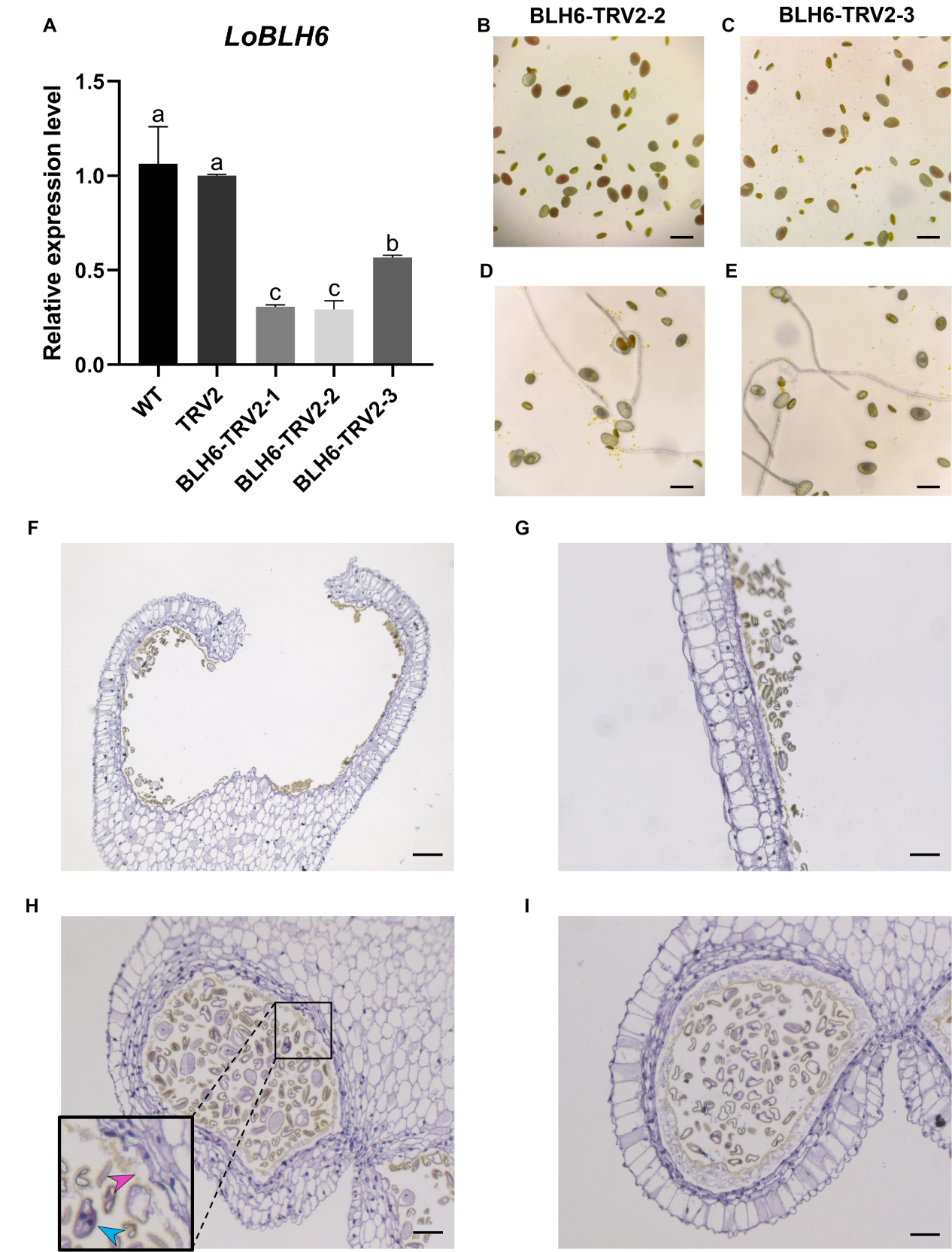


**Fig. S5. *LoBLH6* silenced lines exhibit phenotypes of pollen abortion and abnormal anther development.**

**(*A*)** Expression of *LoBLH6* in TRV2 and LoBLH6 silenced plants. Lily 18S rRNA was used as an internal control. Data are presented as the mean ± SD of 3 replicates, with different letters indicating statistically significant difference (Student–Newman–Keuls test, *P* < 0.05). **(*B*)** and **(*C*)** represent the vitality of mature pollen in 'Siberia' for BLH6-TRV2-2 and BLH6-TRV2-3, respectively, using 2,3,5-Triphenyltetrazolium chloride (TTC) staining. Dark red indicates high pollen vitality, while light red or lack of color indicates low or no vitality. Scale bar = 100 μm. **(*D*)** and **(*E*)** show the germination status of mature pollen in 'Siberia' for BLH6-TRV2-2 and BLH6-TRV2-3, respectively. Fresh pollen was suspended in liquid pollen germination medium and spread on solid germination medium. Photographs were taken under a microscope after 8 hours, with pollen tube length greater than pollen length recorded as normally germinated pollen. Scale bar = 100 μm. **(*F*)** and **(*G*)** represent crosssections of anthers at a bud length of 7.5 cm for TRV2 and BLH6-TRV2-2, respectively. The blue arrows indicate abnormal microspores, and the red arrows indicate abnormal tapetum. Scale bar = 100 μm. **(*H*)** Transverse section of anther at the early stage of flower bud opening in BLH6-TRV2-2 plant. Scale bar = 200 μm. **(*I*)** Longitudinal section of anther at the early stage of flower bud opening in BLH6-TRV2-2 plant. Scale bar = 100 μm.


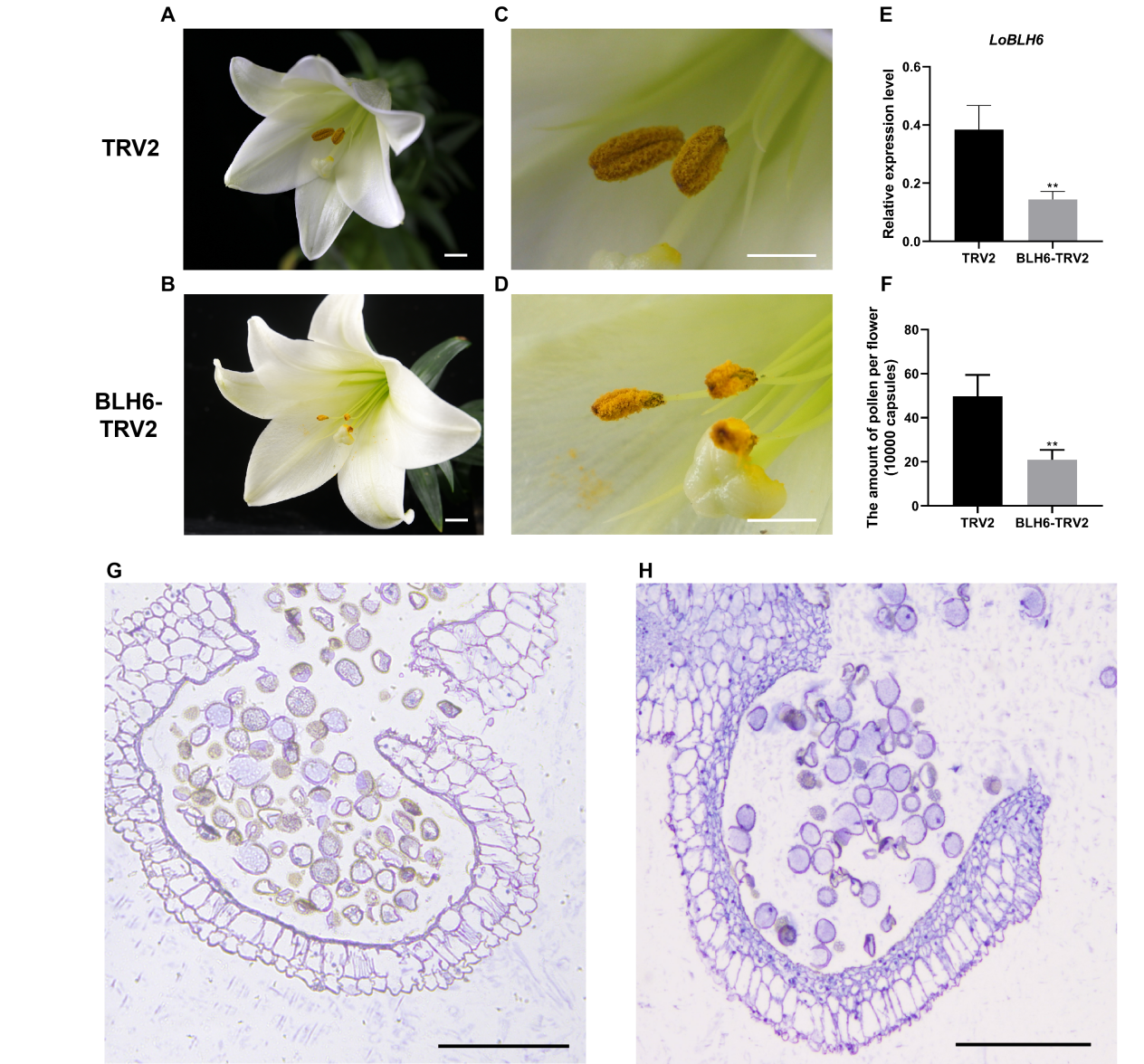


**Fig. S6.** **Silencing of *LoBLH6* in 'White heaven' results in reduced pollen quantity and abnormal anther development.**

1. and **(*B*)** represent the fully opened buds of TRV2 and BLH6-TRV2 in 'White heaven'. TRV indicates tobacco rattle virus, TRV2 indicates the blank control, BLH6-TRV2 indicates the *LoBLH6* silenced plants. Scale bar= 1 cm. **(*C*)** and **(*D*)** show magnified views of the anthers in (*A*) and (*B*). Scale bar= 1 cm. **(*E*)** Expression of *LoBLH6* in TRV2 and BLH6-TRV2 plants. Three replicates are shown (mean ± SD, n = 3). Asterisks indicate significant differences. Student’s *t*-test, ** *P* < 0.01. **(*F*)** Pollen quantity statistics for TRV2 and BLH6-TRV2 plants. Three replicates are shown (mean ± SD, n = 3). Asterisks indicate significant differences. Student’s *t*-test, ** *P* < 0.01. **(*G*)** and **(*H*)** depict cross sections of anthers during the early stage of flower bud opening in 'White Heaven' for TRV2 and BLH6-TRV2, respectively.Scale bar= 400 μm.

**B**

**A**


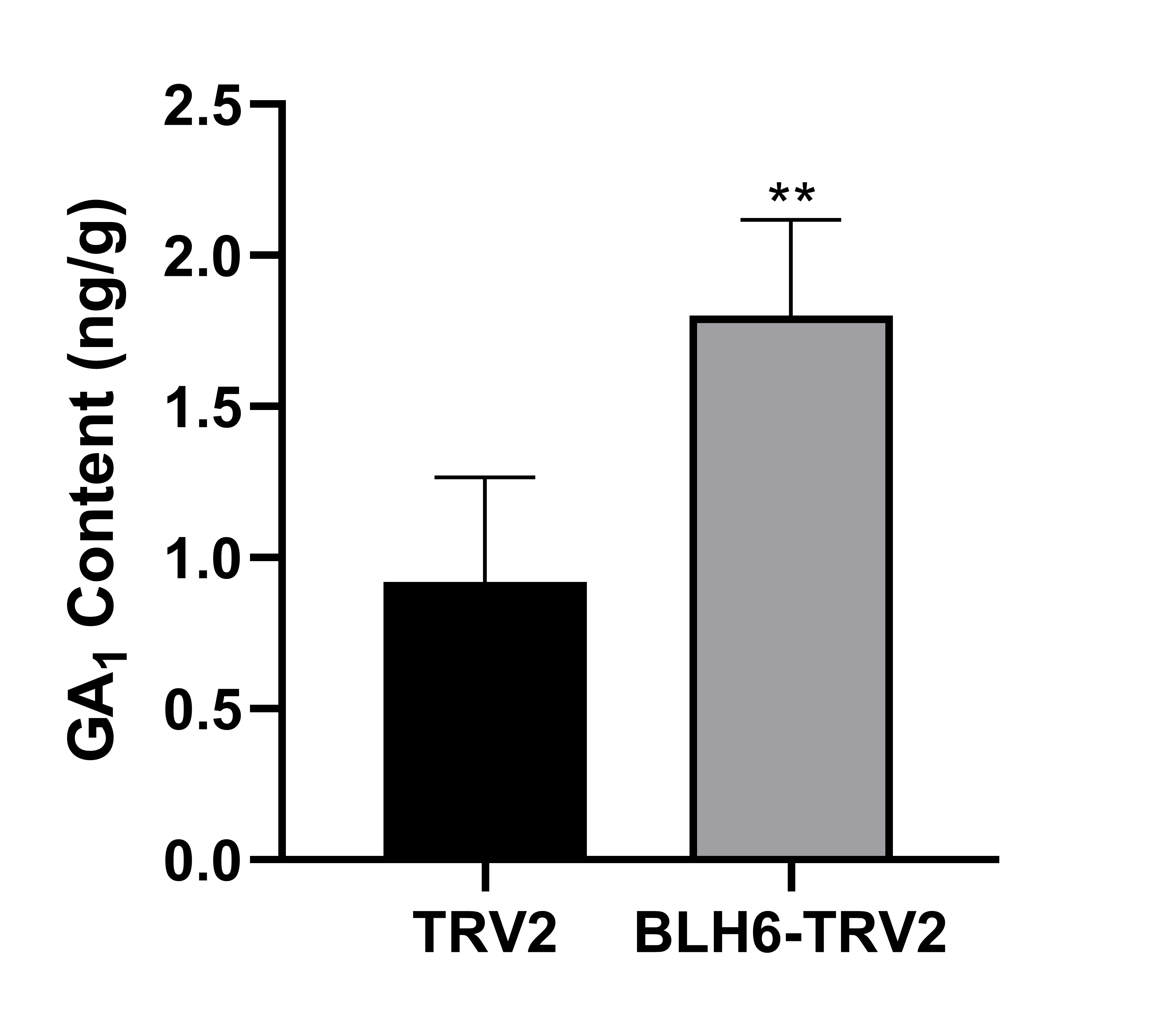

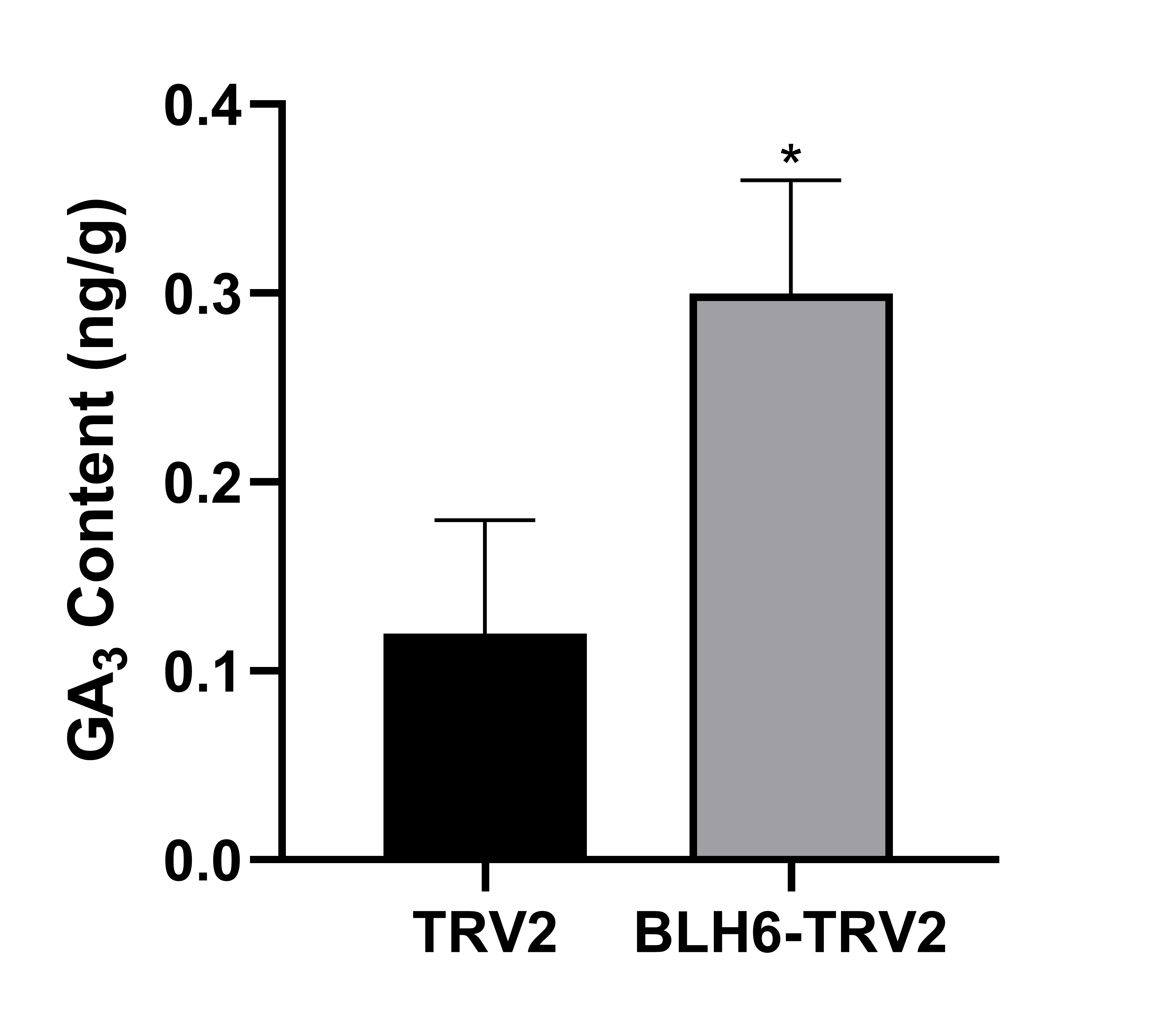


**Fig. S7. Silencing of LoBLH6 leads to an increase in GA content in lily anther.**

**(*A*)** GA_1_ content in mature anther of TRV2 and BLH6-TRV2 lines. **(*B*)** GA_3_ content in TRV2 and BLH6-TRV2 lines. TRV indicates tobacco rattle virus, TRV2 indicates the blank control, and BLH6-TRV2 indicates LoBLH6 silenced plants. Data are the mean ± SD of three biological repeats. Asterisks indicate significant differences (Student's *t*-test, * *P* < 0.01, ** *P* < 0.01).


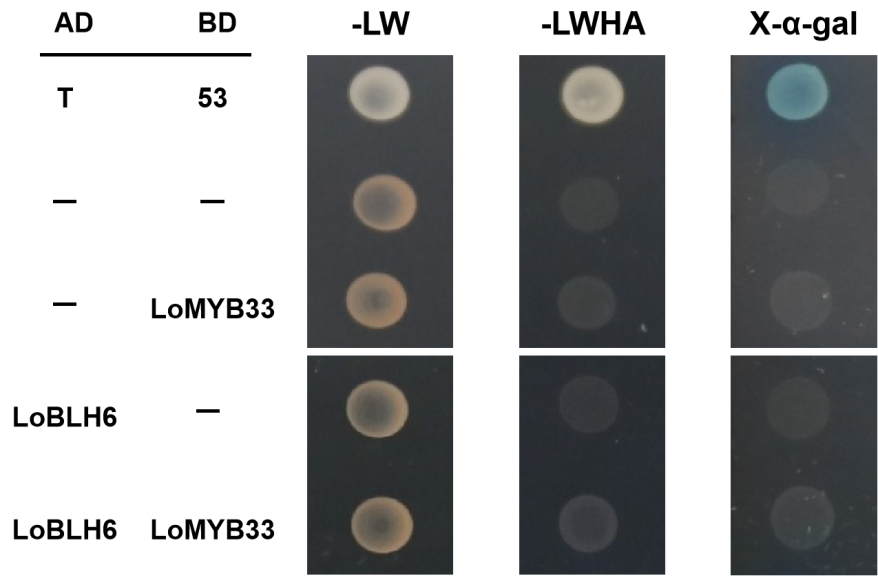


**Fig. S8.** **LoBLH6 does not interact with LoMYB33.**

Yeast strains co-transformed with the pGADT7-T and pGBKT7-53 vectors served as the positive control group. Protein-protein interactions were assessed based on yeast growth on selective media lacking Leu, Trp, His, and Ade (-LWHA). The representative image is from three independent experiments. AD, pGADT7; BD, pGBKT7.

**Table S1. Primers of *LoBLH6* isolation**

| **Gene name** | **Primer sequences (5’-3’)** |
| --- | --- |
| LoBLH6-ORF | F: ATGGCTACTTTCTACTCCGGTTCTACT  R: TCAAGACACAAAGTCATGAATGAGCT |

**Table S2. qRT-PCR primers**

| **Gene name** | **Primer sequences (5’-3’)** |
| --- | --- |
| *LoBLH6* | F: TTCCACAACAACGGCTATGC  R: ACGAGAGCGCCATGAAGAAC |
| *LoGA20ox1* | F: GAGACGAAACATCCACCCTTGA  R: GCAAACGCCTCTCGGACTAA |
| *18S rRNA* | F: AGTTGGTGGAGCGATTTGTCT  R: CCTGTTATTGCCTCAAACTTCC |

**Table S3. Primers used for vector reconstruction**

| **Plasmid name** | **Vectors** | **Primer sequences (5’-3’)** |
| --- | --- | --- |
| pGADT7 | AD-LoBLH6 | F: GCTCATATGGCCATGGAGGCCAGTGAATTCATGGCTACTTTCTACTCCGGTTCTACTG  R: ATTCATCTGCAGCTCGAGCTCGATGGATCCTCAAGACACAAAGTCATGAATGAGCTGA |
| pGADT7 | AD-LoMYB65 | F: GCTCATATGGCCATGGAGGCCAGTGAATTCATGGTGCCGGCGGAGCCAAT  R: ATTCATCTGCAGCTCGAGCTCGATGGATCCTCAAAGCCTTTTGGACTCGGGACAG |
| pGBKT7 | BD-LoBLH6 | F: ACCTGCATATGGCCATGGAGGCCGAATTCATGGCTACTTTCTACTCCGGTTCTACT  R: TTATGCGGCCGCTGCAGGTCGACGGATCCCTCAAGACACAAAGTCATGAATGAGCT |
| pGBKT7 | BD-LoMYB65 | F: ACCTGCATATGGCCATGGAGGCCGAATTCATGGTGCCGGCGGAGCCAAT  R: TTATGCGGCCGCTGCAGGTCGACGGATCCCTCAAAGCCTTTTGGACTCGGGACAGC |
| pCAMBIA1300-nLuc | LoBLH6-nLuc | F: GGAGAGAACACGGGGGACGAGCTCGGTACCATGGCTACTTTCTACTCCGGTTCTACT  R: GCCCCGGGACGCGTACGAGATCTGGTCGACAGACACAAAGTCATGAATGAGCT |
| pCAMBIA1300-nLuc | mLoMYB65-nLuc | F: GGAGAGAACACGGGGGACGAGCTCGGTACCATGGTGCCGGCGGAGCCAAT  R: GCCCCGGGACGCGTACGAGATCTGGTCGACAAGCCTTTTGGACTCGGGACAG |
| pCAMBIA1300-cLuc | LoBLH6-cLuc | F: CAGATCTCGTACGCGTCCCGGGGCGGTACCATGGCTACTTTCTACTCCGGTTCTACT  R: ATGATACGAACGAAAGCTCTGCAGGTCGACTCAAGACACAAAGTCATGAATGAGCT |
| pCAMBIA1300-cLuc | mLoMYB65-cLuc | F: CAGATCTCGTACGCGTCCCGGGGCGGTACCATGGTGCCGGCGGAGCCAAT  R: ATGATACGAACGAAAGCTCTGCAGGTCGACTCAAAGCCTTTTGGACTCGGGACAG |
| pSPYNE173 | LoBLH6-YNE | F: GGGCCCAGGCCTACTAGTGGATCCGTCGACATGGCTACTTTCTACTCCGGTTCTACT  R: GAGCTCCTACCCGGGAGCGGTACCCTCGAGTCAAGACACAAAGTCATGAATGAGCT |
| pSPYNE173 | mLoMYB65-YNE | F: GGGCCCAGGCCTACTAGTGGATCCGTCGACATGGTGCCGGCGGAGCCAAT  R: GAGCTCCTACCCGGGAGCGGTACCCTCGAGTCAAAGCCTTTTGGACTCGGGACAG |
| pSPYCE(M) | LoBLH6-YCE | F: GGGCCCAGGCCTACTAGTGGATCCGTCGACATGGCTACTTTCTACTCCGGTTCTACT  R: GAGCTCCTACCCGGGAGCGGTACCCTCGAGTCAAGACACAAAGTCATGAATGAGCT |
| pSPYCE(M) | mLoMYB65-YCE | F: GGGCCCAGGCCTACTAGTGGATCCGTCGACATGGTGCCGGCGGAGCCAAT  R: TGGGTACATCCCGGGAGCGGTACCCTCGAGAAGCCTTTTGGACTCGGGACAG |
| pMAL-p5x | MBP-LoMYB65 | F: CTCGGGATCGAGGGAAGGATTTCACATATGATGGTGCCGGCGGAGCCAAT  R: TATTTAATTACCTGCAGGGAATTCGGATCCTCAAAGCCTTTTGGACTCGGGACAGC |
| pGEX-4T-1 | GST-LoBLH6 | F: CATCCTCCAAAATCGGATCTGGTTCCGCGTGGATCCATGGCTACTTTCTACTCCGGTTCTACT  R: CAGTCAGTCACGATGCGGCCGCTCGAGTCGACTCAAGACACAAAGTCATGAATGAGCT |
| pBD | pBD-LoBLH6 | F: GACTGTATCGCCGACCGGTAGGCCTATGGCTACTTTCTACTCCGGTTCTACT  R: ATGAAACCAGAGTTAAAGGCCTTCAAGACACAAAGTCATGAATGAGCT |
| pBD-VP16 | pBD-LoBLH6-VP16 | F: GACCTGCATATGGCCATGGAGGCCGAATTCATGGCTACTTTCTACTCCGGTTCTA  R: GCTGACATCGGTCGGGGGGGCACGGATCCCAGACACAAAGTCATGAATGAGCT |
| pTRV2 | pTRV2-LoBLH6 | F: CTAGAAGGCCTCCATGGGGAATGGCTACTTTCTACTCCGGTTCTACT  R: TCGAGACGCGTGAGCTCGGTCAACATCTCGTTTCTTCCGTCTCT |
| pHis2 | pHis2-LoGA20ox1 | F: TTGTAATACGACTCACTATAGGGCGAATTCGGTAACTTTGACCAACTAAA  R: CCGCGGATCGATTCGCGAACGCGTGAGCTCCATGAATGTCAGTCAACAT |
| pHis2 | pHis2-mLoGA20ox1 | F: TTGTAATACGACTCACTATAGGGCGAATTCGGTAACTGGGGGCAACTAA  R: CCGCGGATCGATTCGCGAACGCGTGAGCTCCATGAATTTTTCCCCC |
| pGreenII 0800-LUC | *proLoGA20ox1*-Luc | F: TGTAATACGACTCACTATAGGGCGAATTGGGTACCGGAGTGATAAAACTCTTCTTGTAA  R: TTCGATATCAAGCTTATCGATACCGTCGACCTCGAGGGGAACTATGTGTGTTTGA |
| pGreenII 62-SK | LoBLH6-SK | F: CTAGTGGATCCCCCGGGCTGCAGGAATTCATGGCTACTTTCTACTCCGGTTCTACT  R: CAGCGAATTGGTACCGGGCCCCCCCTCGAGTCAAGACACAAAGTCATGAATGAGCT |
| pGreenII 62-SK | mLoMYB65-SK | F: GAGCTCCACCGCGGTGGCGGCCGCTCTAGAATGGTGCCGGCGGAGCCAAT  R: TGATATCGAATTCCTGCAGCCCGGGGGATCCTCAAAGCCTTTTGGACTCGGGACAG |
| pCOLD I | pCOLD-LoBLH6 | F: CATATGGAGCTCGGTACCATGGCTACTTTCTACTCCGGTTCTACT  R: GGTCGACAAGCTTGAATTCTCAAGACACAAAGTCATGAATGAGCT |
| pCAMBIA1300-GFP | p1300-nGFP-LoBLH6 | F: CTCGGCATGGACGAGCTGTACAAGGTCGACATGGCTACTTTCTACTCCGGTTCTACT  R: ATGTTTGAACGATCGGGGAAATTCGAGCTC TCAAGACACAAAGTCATGAATGAGCT |
| pCAMBIA1300-GFP | p1300-LoBLH6-cGFP | F: AGAAAGCTTCTGCAGGGGCCCGGGGTCGACATGGCTACTTTCTACTCCGGTTCTACT  R: CAGCTCCTCGCCCTTGCTCACCATGGTACCAGACACAAAGTCATGAATGAGCT |

**Table S4. Primers used for RNA *in situ* hybridization**

| **Primer name** | **Primer sequences (5’-3’)** |
| --- | --- |
| *LoBLH6*-antisense | F: CTACCGGTAGTGTCGGTATGAT  R: GATTTAGGTGACACTATAGAATGCTCAGACTGCATACATGAGTTCTG |
| *LoBLH6*-sense | F: TGTAATACGACTCACTATAGGGCTACCGGTAGTGTCGGTATGAT  R: CAGACTGCATACATGAGTTCTG |
| *LoMYB65*-antisense | F: GAGAGTCAACAGAACTCGGGTGTC  R: GATTTAGGTGACACTATAGAATGCTGATGTGTGGCTGAAAGAAAAGGTG |
| *LoMYB65*-sense | F: TGTAATACGACTCACTATAGGGGAGAGTCAACAGAACTCGGGTGTC  R: GATGTGTGGCTGAAAGAAAAGGTG |

**Table S5. Hypothetical *GA20oxs* transcripts compared in the transcriptome of 'Siberia' anther development**

| **Sequences producing significant alignments** | **Score (bits)** | **E Value** |
| --- | --- | --- |
| transcript_HQ_LoMix_transcript23735/f3p0/1477 (***LoGA20ox1***) | 2248 | 0 |
| transcript_HQ_LoMix_transcript17030/f2p0/1880 | 36 | 0.49 |
| transcript_HQ_LoMix_transcript16095/f3p0/1997 | 36 | 0.49 |
| transcript_HQ_LoMix_transcript11867/f2p0/2347 | 36 | 0.49 |
| transcript_HQ_LoMix_transcript785/f2p0/4495 | 34 | 1.9 |
| transcript_HQ_LoMix_transcript739/f2p0/4562 | 34 | 1.9 |
| transcript_HQ_LoMix_transcript28879/f3p0/1162 | 34 | 1.9 |
| transcript_HQ_LoMix_transcript26921/f6p0/1240 | 34 | 1.9 |
| transcript_HQ_LoMix_transcript27679/f2p0/1222 | 34 | 1.9 |
| transcript_HQ_LoMix_transcript261/f4p0/5363 | 34 | 1.9 |
| transcript_HQ_LoMix_transcript25965/f2p0/1326 | 34 | 1.9 |
| transcript_HQ_LoMix_transcript185/f2p0/5685 | 34 | 1.9 |
| transcript_HQ_LoMix_transcript17290/f3p0/1859 | 34 | 1.9 |
| transcript_HQ_LoMix_transcript17274/f6p0/1891 | 34 | 1.9 |
