# Supplementary material for: LoBLH6 interacts with LoMYB65 to regulate anther development through feedback regulation of gibberellin synthesis in lily
Source: Hortic Res. 2024 Dec 4;12(3):uhae339. doi: 10.1093/hr/uhae339 (PMC11886847; doi:10.1093/hr/uhae339)
Supplement: Web_Material_uhae339 [file web_material_uhae339.zip › Supplementary Table.docx]

**Table S1. Primers of *LoBLH6* isolation**

| **Gene name** | **Primer sequences (5’-3’)** |
| --- | --- |
| LoBLH6-ORF | F: ATGGCTACTTTCTACTCCGGTTCTACT  R: TCAAGACACAAAGTCATGAATGAGCT |

**Table S2. qRT-PCR primers**

| **Gene name** | **Primer sequences (5’-3’)** |
| --- | --- |
| *LoBLH6* | F: TTCCACAACAACGGCTATGC  R: ACGAGAGCGCCATGAAGAAC |
| *LoGA20ox1* | F: GAGACGAAACATCCACCCTTGA  R: GCAAACGCCTCTCGGACTAA |
| *18S rRNA* | F: AGTTGGTGGAGCGATTTGTCT  R: CCTGTTATTGCCTCAAACTTCC |

**Table S3. Primers used for vector reconstruction**

| **Plasmid name** | **Vectors** | **Primer sequences (5’-3’)** |
| --- | --- | --- |
| pGADT7 | AD-LoBLH6 | F: GCTCATATGGCCATGGAGGCCAGTGAATTCATGGCTACTTTCTACTCCGGTTCTACTG  R: ATTCATCTGCAGCTCGAGCTCGATGGATCCTCAAGACACAAAGTCATGAATGAGCTGA |
| pGADT7 | AD-LoMYB65 | F: GCTCATATGGCCATGGAGGCCAGTGAATTCATGGTGCCGGCGGAGCCAAT  R: ATTCATCTGCAGCTCGAGCTCGATGGATCCTCAAAGCCTTTTGGACTCGGGACAG |
| pGBKT7 | BD-LoBLH6 | F: ACCTGCATATGGCCATGGAGGCCGAATTCATGGCTACTTTCTACTCCGGTTCTACT  R: TTATGCGGCCGCTGCAGGTCGACGGATCCCTCAAGACACAAAGTCATGAATGAGCT |
| pGBKT7 | BD-LoMYB65 | F: ACCTGCATATGGCCATGGAGGCCGAATTCATGGTGCCGGCGGAGCCAAT  R: TTATGCGGCCGCTGCAGGTCGACGGATCCCTCAAAGCCTTTTGGACTCGGGACAGC |
| pCAMBIA1300-nLuc | LoBLH6-nLuc | F: GGAGAGAACACGGGGGACGAGCTCGGTACCATGGCTACTTTCTACTCCGGTTCTACT  R: GCCCCGGGACGCGTACGAGATCTGGTCGACAGACACAAAGTCATGAATGAGCT |
| pCAMBIA1300-nLuc | mLoMYB65-nLuc | F: GGAGAGAACACGGGGGACGAGCTCGGTACCATGGTGCCGGCGGAGCCAAT  R: GCCCCGGGACGCGTACGAGATCTGGTCGACAAGCCTTTTGGACTCGGGACAG |
| pCAMBIA1300-cLuc | LoBLH6-cLuc | F: CAGATCTCGTACGCGTCCCGGGGCGGTACCATGGCTACTTTCTACTCCGGTTCTACT  R: ATGATACGAACGAAAGCTCTGCAGGTCGACTCAAGACACAAAGTCATGAATGAGCT |
| pCAMBIA1300-cLuc | mLoMYB65-cLuc | F: CAGATCTCGTACGCGTCCCGGGGCGGTACCATGGTGCCGGCGGAGCCAAT  R: ATGATACGAACGAAAGCTCTGCAGGTCGACTCAAAGCCTTTTGGACTCGGGACAG |
| pSPYNE173 | LoBLH6-YNE | F: GGGCCCAGGCCTACTAGTGGATCCGTCGACATGGCTACTTTCTACTCCGGTTCTACT  R: GAGCTCCTACCCGGGAGCGGTACCCTCGAGTCAAGACACAAAGTCATGAATGAGCT |
| pSPYNE173 | mLoMYB65-YNE | F: GGGCCCAGGCCTACTAGTGGATCCGTCGACATGGTGCCGGCGGAGCCAAT  R: GAGCTCCTACCCGGGAGCGGTACCCTCGAGTCAAAGCCTTTTGGACTCGGGACAG |
| pSPYCE(M) | LoBLH6-YCE | F: GGGCCCAGGCCTACTAGTGGATCCGTCGACATGGCTACTTTCTACTCCGGTTCTACT  R: GAGCTCCTACCCGGGAGCGGTACCCTCGAGTCAAGACACAAAGTCATGAATGAGCT |
| pSPYCE(M) | mLoMYB65-YCE | F: GGGCCCAGGCCTACTAGTGGATCCGTCGACATGGTGCCGGCGGAGCCAAT  R: TGGGTACATCCCGGGAGCGGTACCCTCGAGAAGCCTTTTGGACTCGGGACAG |
| pMAL-p5x | MBP-LoMYB65 | F: CTCGGGATCGAGGGAAGGATTTCACATATGATGGTGCCGGCGGAGCCAAT  R: TATTTAATTACCTGCAGGGAATTCGGATCCTCAAAGCCTTTTGGACTCGGGACAGC |
| pGEX-4T-1 | GST-LoBLH6 | F: CATCCTCCAAAATCGGATCTGGTTCCGCGTGGATCCATGGCTACTTTCTACTCCGGTTCTACT  R: CAGTCAGTCACGATGCGGCCGCTCGAGTCGACTCAAGACACAAAGTCATGAATGAGCT |
| pBD | pBD-LoBLH6 | F: GACTGTATCGCCGACCGGTAGGCCTATGGCTACTTTCTACTCCGGTTCTACT  R: ATGAAACCAGAGTTAAAGGCCTTCAAGACACAAAGTCATGAATGAGCT |
| pBD-VP16 | pBD-LoBLH6-VP16 | F: GACCTGCATATGGCCATGGAGGCCGAATTCATGGCTACTTTCTACTCCGGTTCTA  R: GCTGACATCGGTCGGGGGGGCACGGATCCCAGACACAAAGTCATGAATGAGCT |
| pTRV2 | pTRV2-LoBLH6 | F: CTAGAAGGCCTCCATGGGGAATGGCTACTTTCTACTCCGGTTCTACT  R: TCGAGACGCGTGAGCTCGGTCAACATCTCGTTTCTTCCGTCTCT |
| pHis2 | pHis2-LoGA20ox1 | F: TTGTAATACGACTCACTATAGGGCGAATTCGGTAACTTTGACCAACTAAA  R: CCGCGGATCGATTCGCGAACGCGTGAGCTCCATGAATGTCAGTCAACAT |
| pHis2 | pHis2-mLoGA20ox1 | F: TTGTAATACGACTCACTATAGGGCGAATTCGGTAACTGGGGGCAACTAA  R: CCGCGGATCGATTCGCGAACGCGTGAGCTCCATGAATTTTTCCCCC |
| pGreenII 0800-LUC | *proLoGA20ox1*-Luc | F: TGTAATACGACTCACTATAGGGCGAATTGGGTACCGGAGTGATAAAACTCTTCTTGTAA  R: TTCGATATCAAGCTTATCGATACCGTCGACCTCGAGGGGAACTATGTGTGTTTGA |
| pGreenII 62-SK | LoBLH6-SK | F: CTAGTGGATCCCCCGGGCTGCAGGAATTCATGGCTACTTTCTACTCCGGTTCTACT  R: CAGCGAATTGGTACCGGGCCCCCCCTCGAGTCAAGACACAAAGTCATGAATGAGCT |
| pGreenII 62-SK | mLoMYB65-SK | F: GAGCTCCACCGCGGTGGCGGCCGCTCTAGAATGGTGCCGGCGGAGCCAAT  R: TGATATCGAATTCCTGCAGCCCGGGGGATCCTCAAAGCCTTTTGGACTCGGGACAG |
| pCOLD I | pCOLD-LoBLH6 | F: CATATGGAGCTCGGTACCATGGCTACTTTCTACTCCGGTTCTACT  R: GGTCGACAAGCTTGAATTCTCAAGACACAAAGTCATGAATGAGCT |
| pCAMBIA1300-GFP | p1300-nGFP-LoBLH6 | F: CTCGGCATGGACGAGCTGTACAAGGTCGACATGGCTACTTTCTACTCCGGTTCTACT  R: ATGTTTGAACGATCGGGGAAATTCGAGCTC TCAAGACACAAAGTCATGAATGAGCT |
| pCAMBIA1300-GFP | p1300-LoBLH6-cGFP | F: AGAAAGCTTCTGCAGGGGCCCGGGGTCGACATGGCTACTTTCTACTCCGGTTCTACT  R: CAGCTCCTCGCCCTTGCTCACCATGGTACCAGACACAAAGTCATGAATGAGCT |

**Table S4. Primers used for RNA *in situ* hybridization**

| **Primer name** | **Primer sequences (5’-3’)** |
| --- | --- |
| *LoBLH6*-antisense | F: CTACCGGTAGTGTCGGTATGAT  R: GATTTAGGTGACACTATAGAATGCTCAGACTGCATACATGAGTTCTG |
| *LoBLH6*-sense | F: TGTAATACGACTCACTATAGGGCTACCGGTAGTGTCGGTATGAT  R: CAGACTGCATACATGAGTTCTG |
| *LoMYB65*-antisense | F: GAGAGTCAACAGAACTCGGGTGTC  R: GATTTAGGTGACACTATAGAATGCTGATGTGTGGCTGAAAGAAAAGGTG |
| *LoMYB65*-sense | F: TGTAATACGACTCACTATAGGGGAGAGTCAACAGAACTCGGGTGTC  R: GATGTGTGGCTGAAAGAAAAGGTG |

**Table S5. Hypothetical *GA20oxs* transcripts compared in the transcriptome of 'Siberia' anther development**

| **Sequences producing significant alignments** | **Score (bits)** | **E Value** |
| --- | --- | --- |
| transcript_HQ_LoMix_transcript23735/f3p0/1477 (***LoGA20ox1***) | 2248 | 0 |
| transcript_HQ_LoMix_transcript17030/f2p0/1880 | 36 | 0.49 |
| transcript_HQ_LoMix_transcript16095/f3p0/1997 | 36 | 0.49 |
| transcript_HQ_LoMix_transcript11867/f2p0/2347 | 36 | 0.49 |
| transcript_HQ_LoMix_transcript785/f2p0/4495 | 34 | 1.9 |
| transcript_HQ_LoMix_transcript739/f2p0/4562 | 34 | 1.9 |
| transcript_HQ_LoMix_transcript28879/f3p0/1162 | 34 | 1.9 |
| transcript_HQ_LoMix_transcript26921/f6p0/1240 | 34 | 1.9 |
| transcript_HQ_LoMix_transcript27679/f2p0/1222 | 34 | 1.9 |
| transcript_HQ_LoMix_transcript261/f4p0/5363 | 34 | 1.9 |
| transcript_HQ_LoMix_transcript25965/f2p0/1326 | 34 | 1.9 |
| transcript_HQ_LoMix_transcript185/f2p0/5685 | 34 | 1.9 |
| transcript_HQ_LoMix_transcript17290/f3p0/1859 | 34 | 1.9 |
| transcript_HQ_LoMix_transcript17274/f6p0/1891 | 34 | 1.9 |
