# Supplementary figures and images for: LoBLH6 interacts with LoMYB65 to regulate anther development through feedback regulation of gibberellin synthesis in lily
Source: Hortic Res. 2024 Dec 4;12(3):uhae339. doi: 10.1093/hr/uhae339 (PMC11886847; doi:10.1093/hr/uhae339)

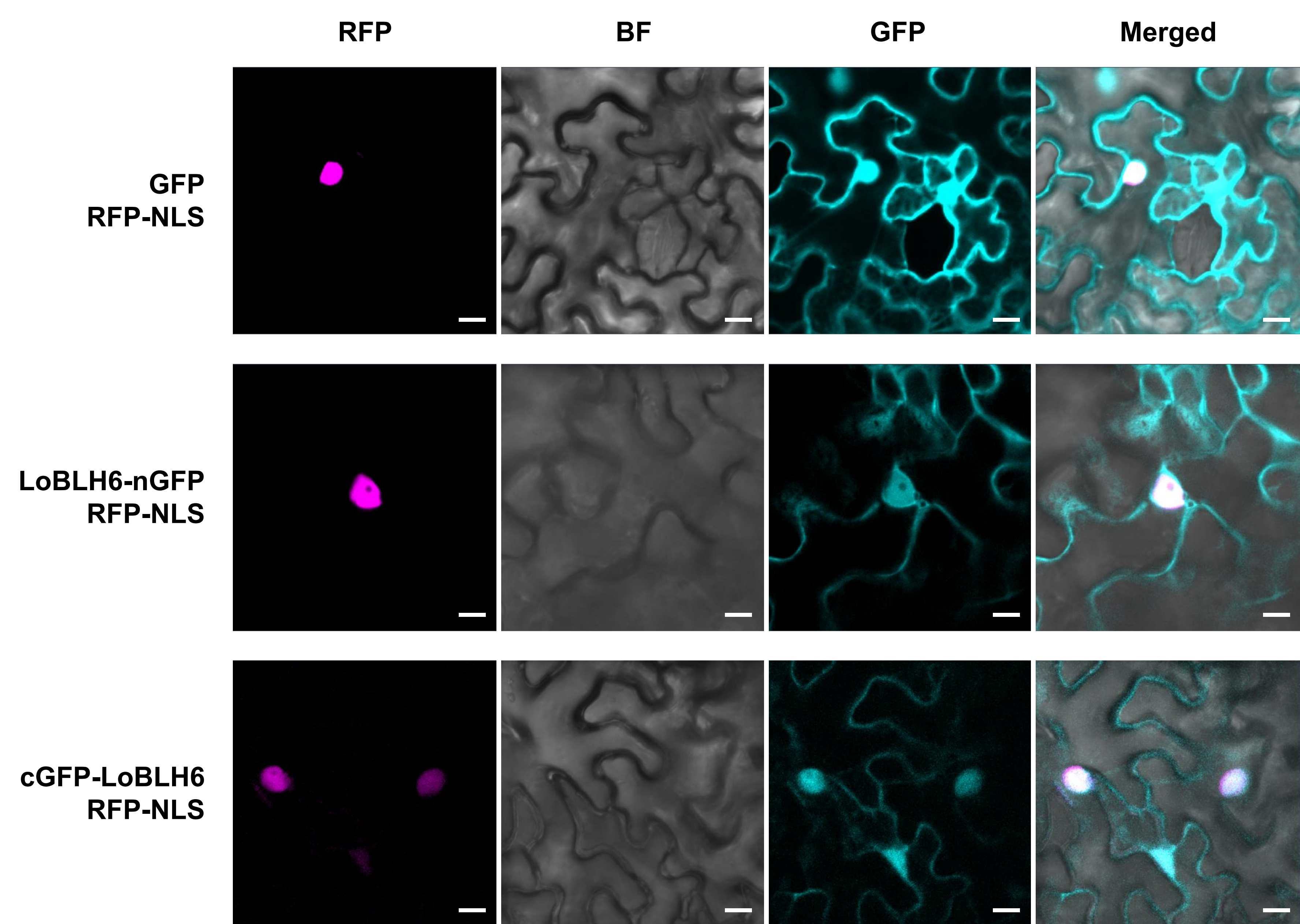

Supplement: Web_Material_uhae339 [file web_material_uhae339.zip › Fig. S2.pdf]

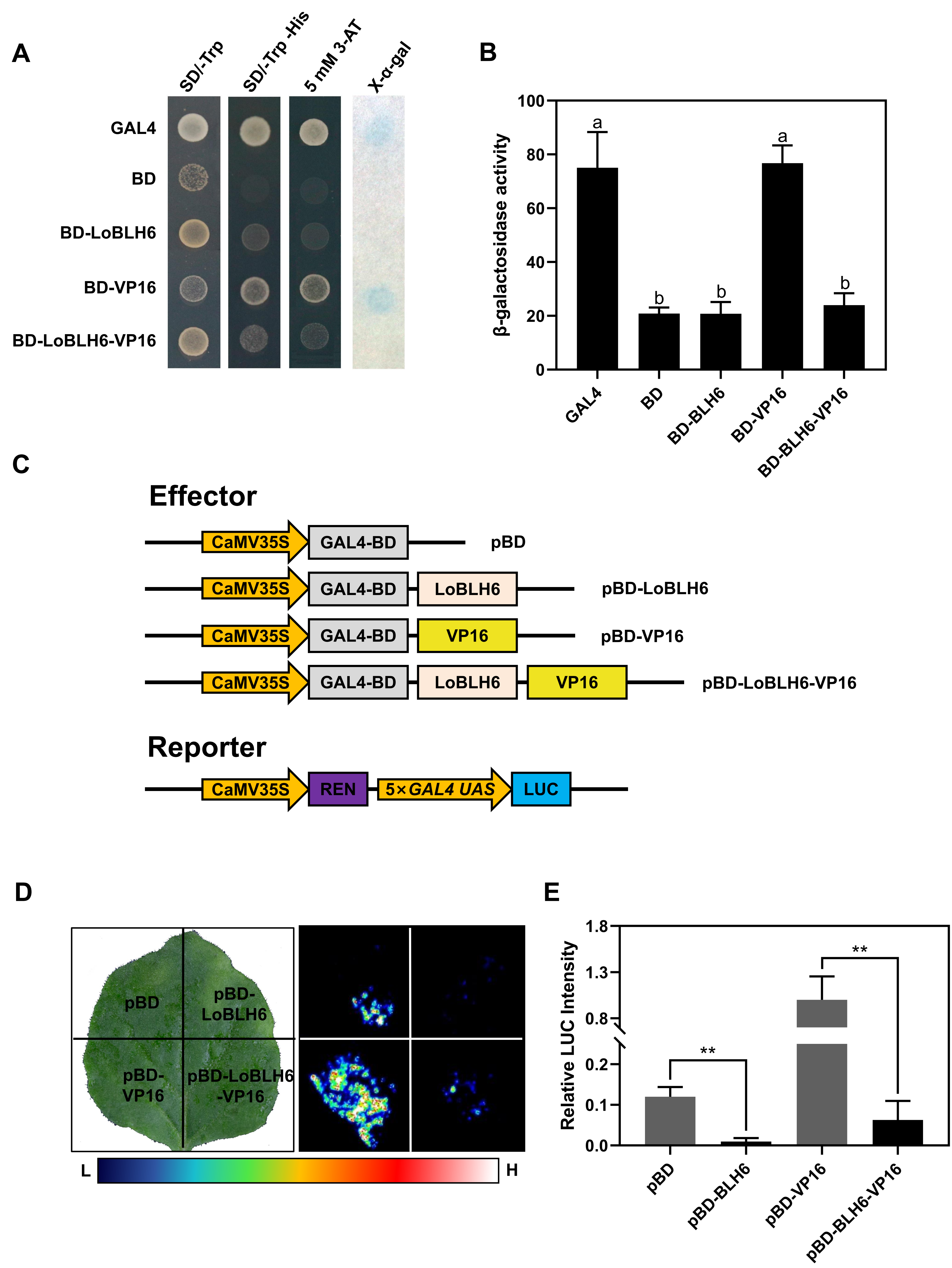

Supplement: Web_Material_uhae339 [file web_material_uhae339.zip › Fig. S3.pdf]

A

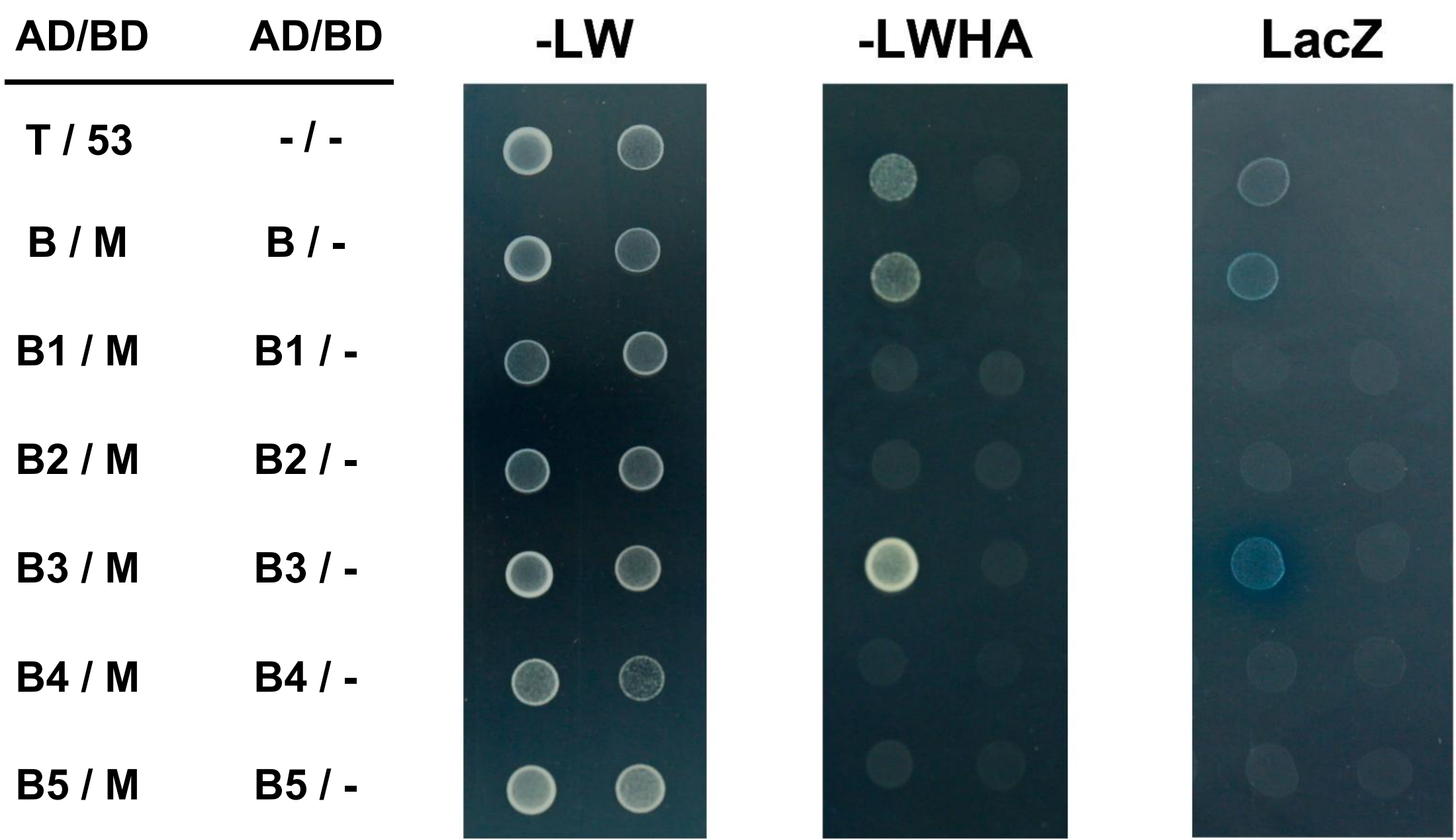

C

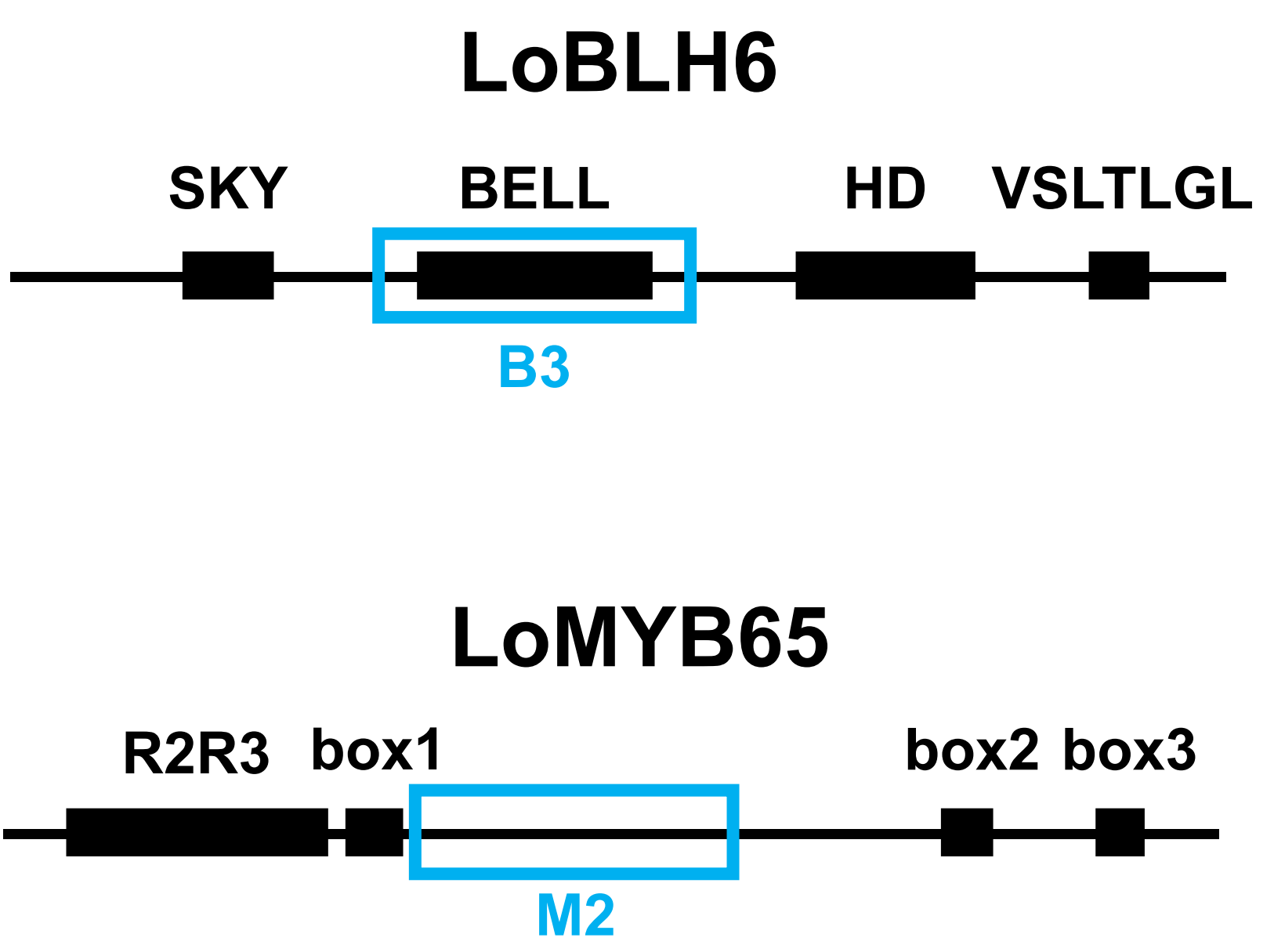

B

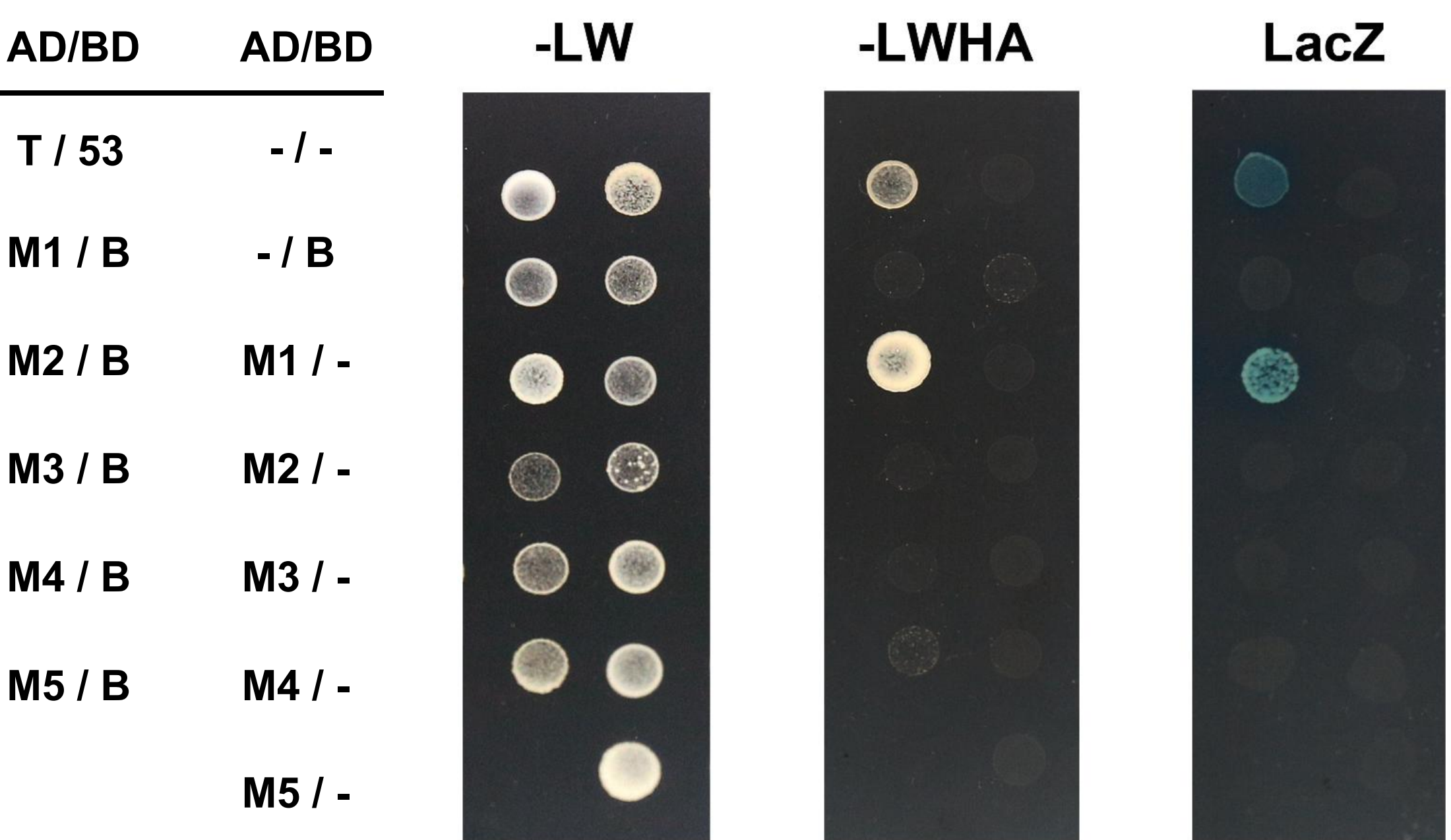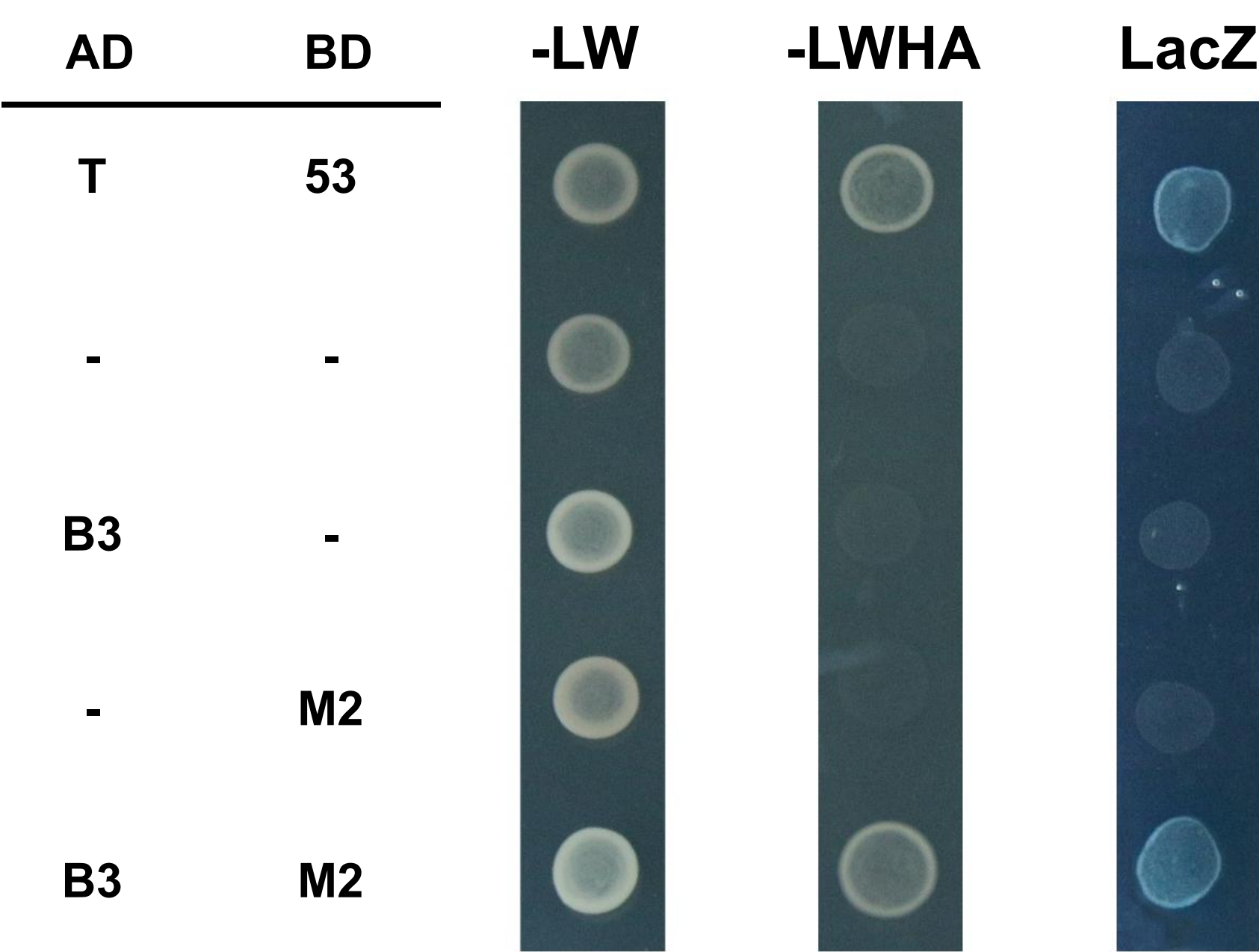

Supplement: Web_Material_uhae339 [file web_material_uhae339.zip › Fig. S4.pdf]

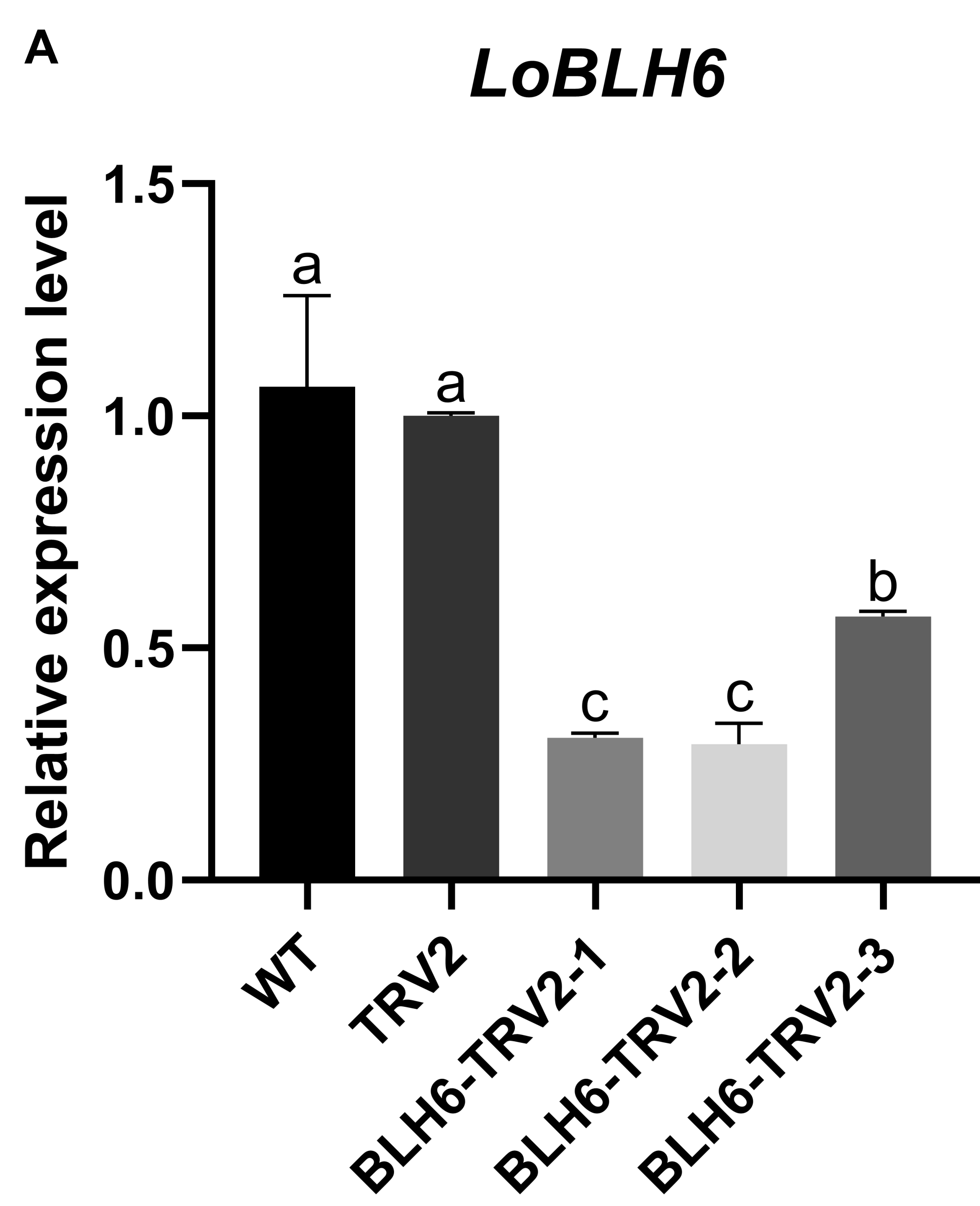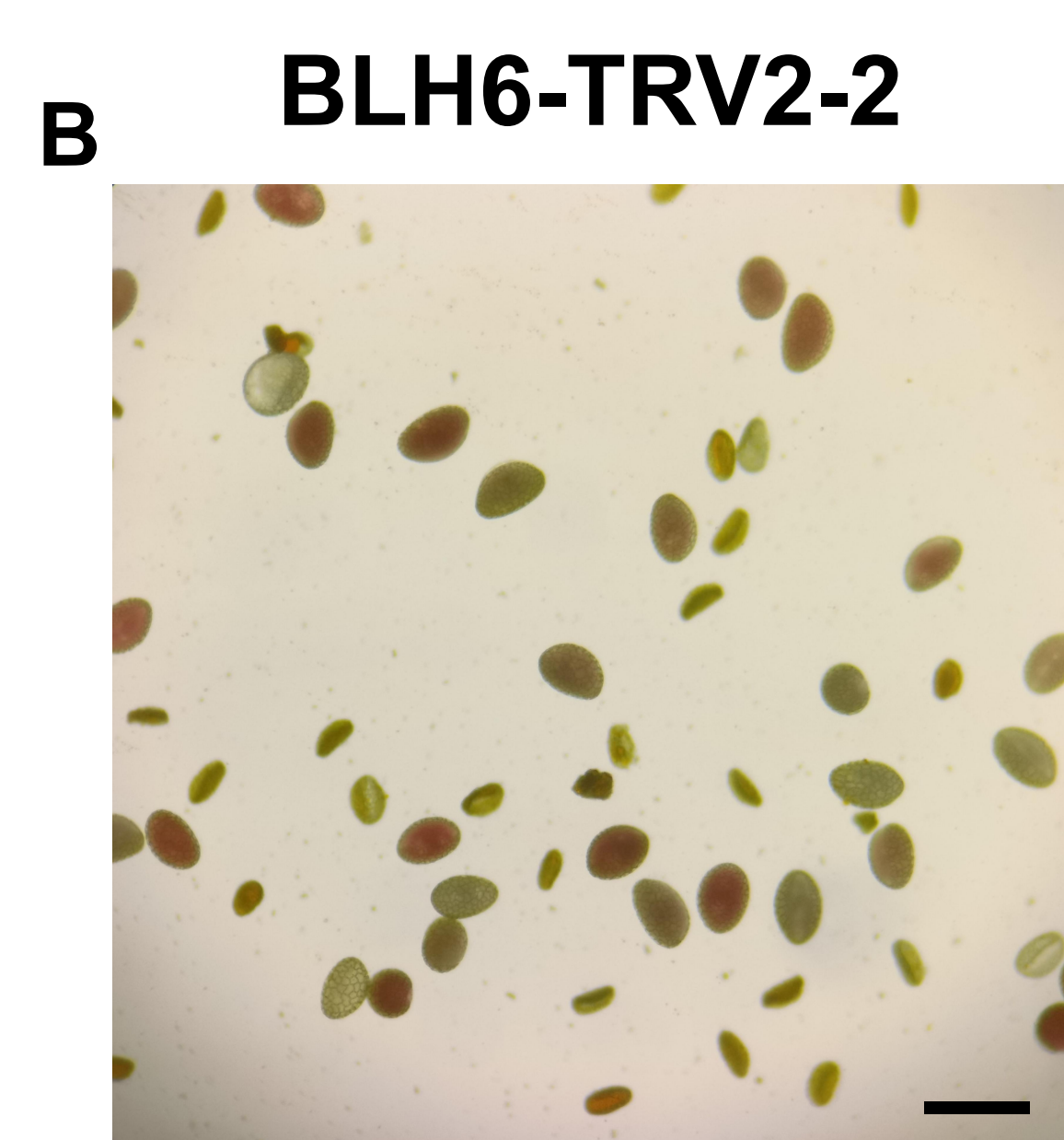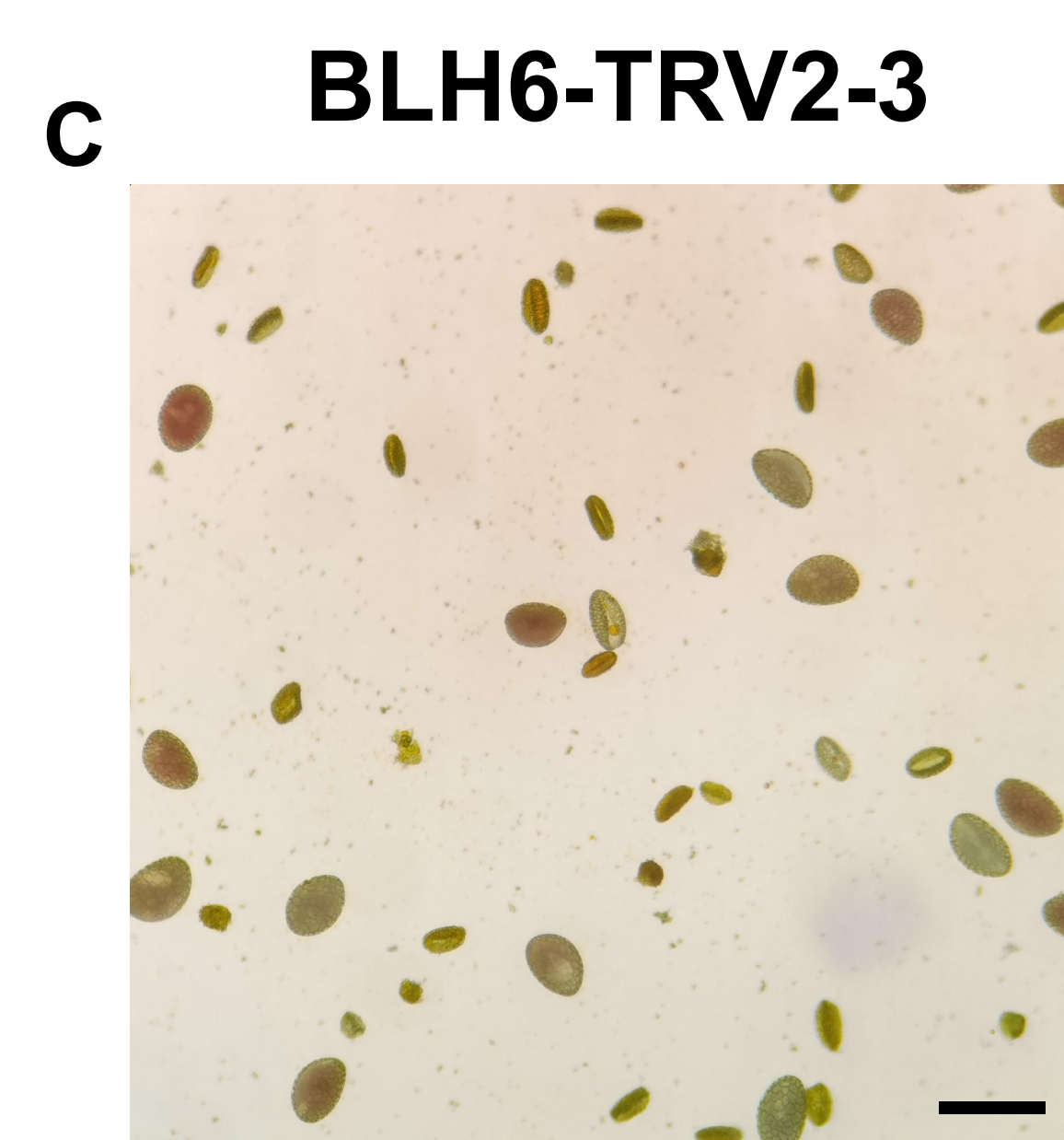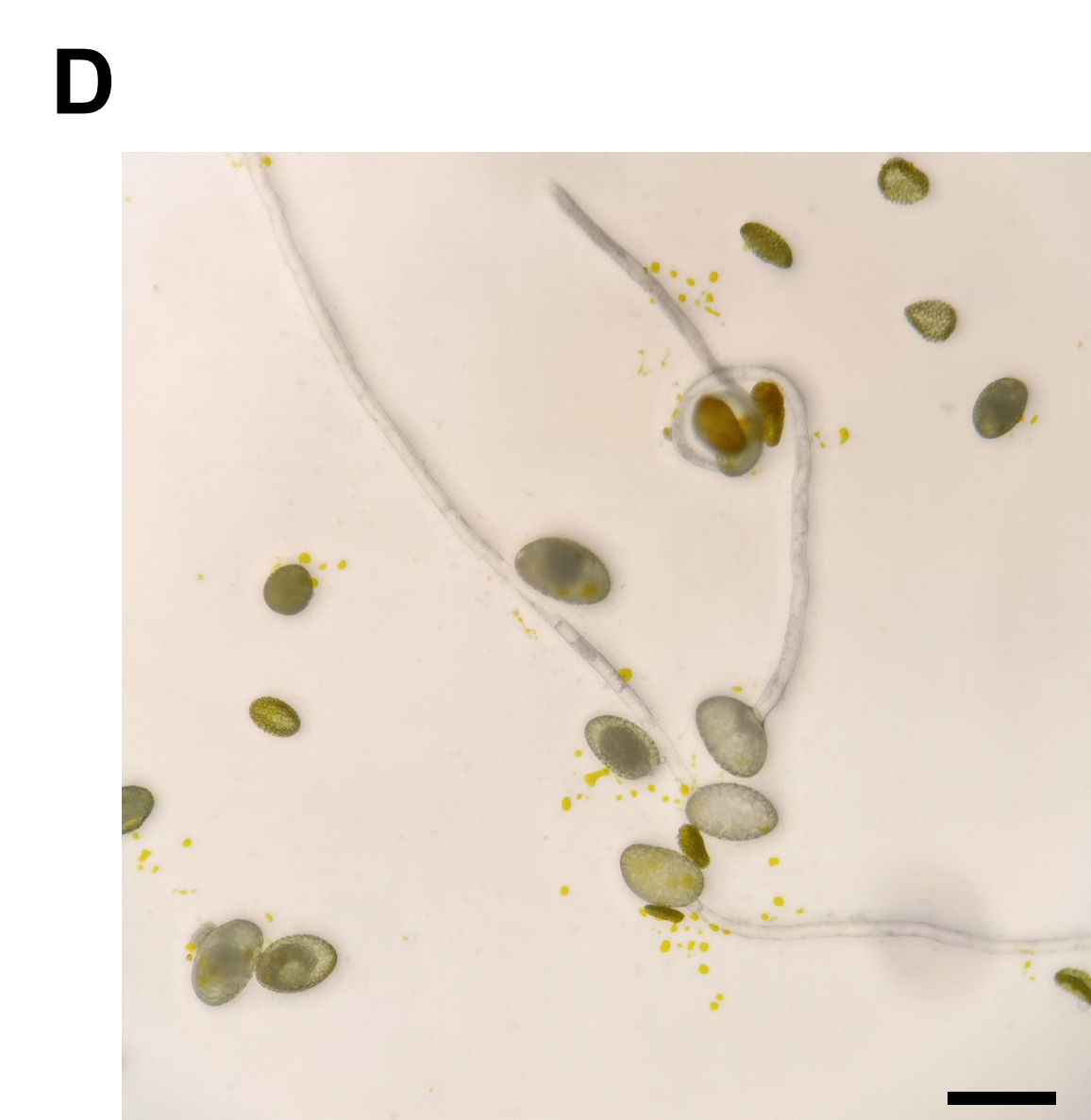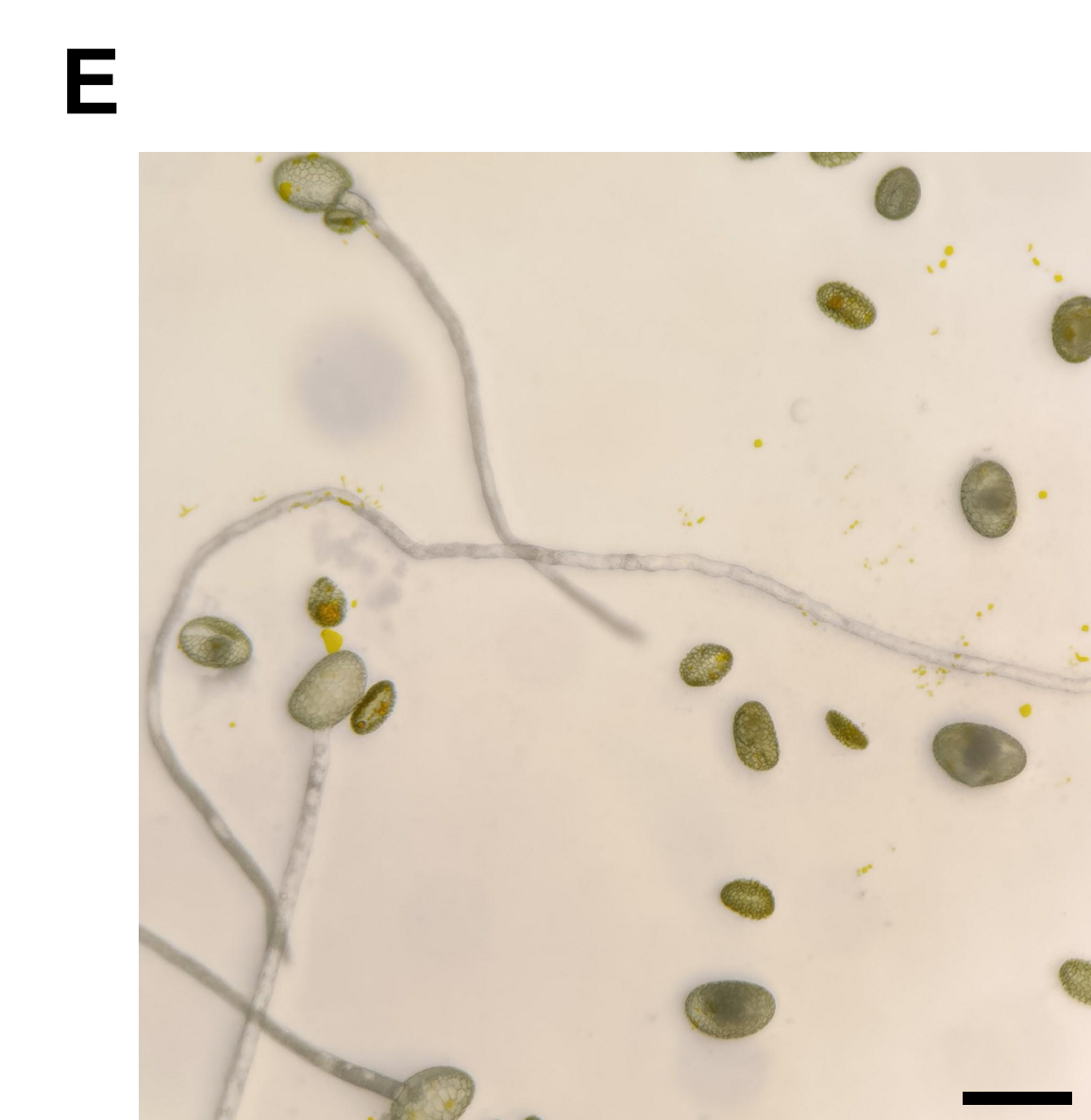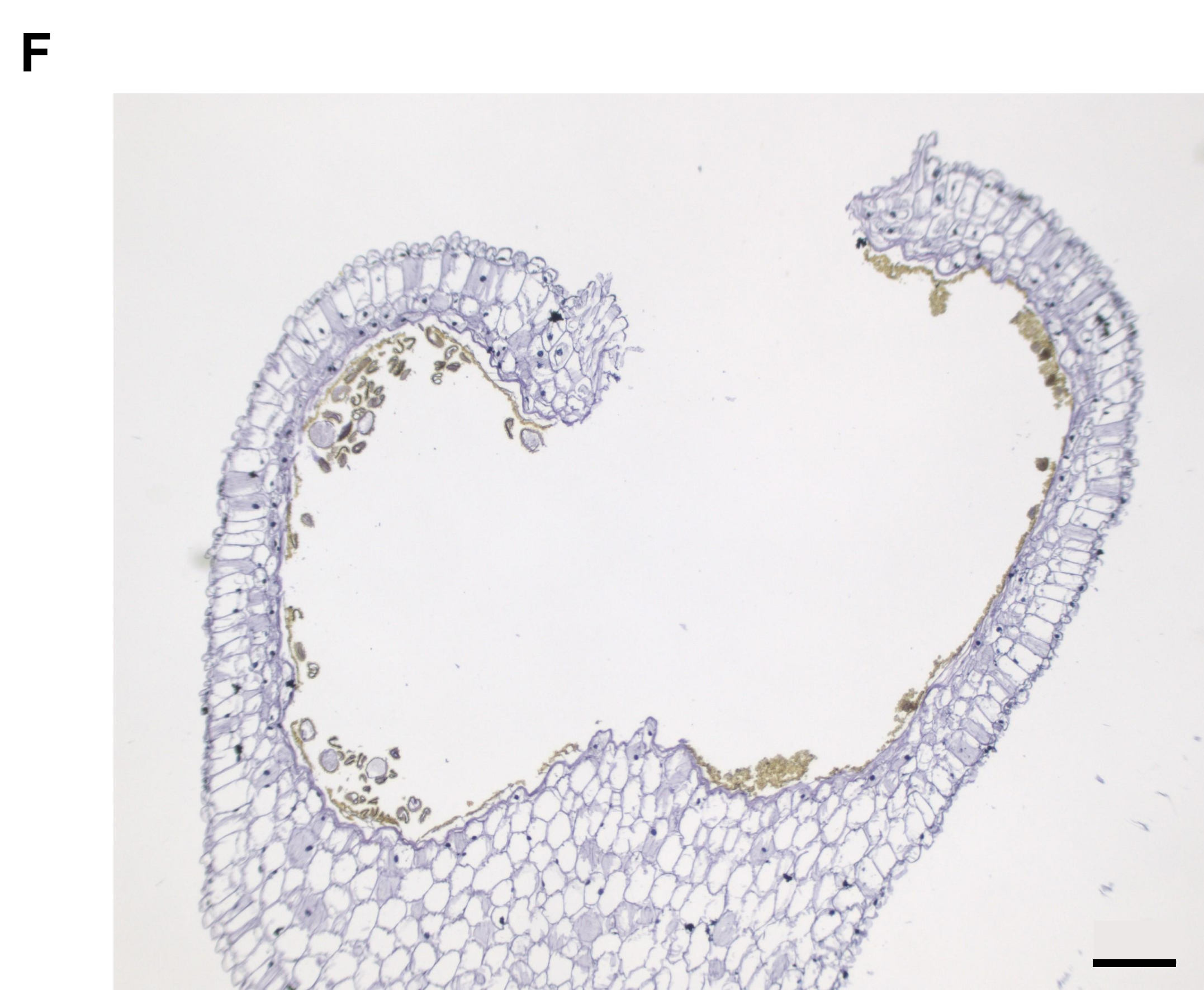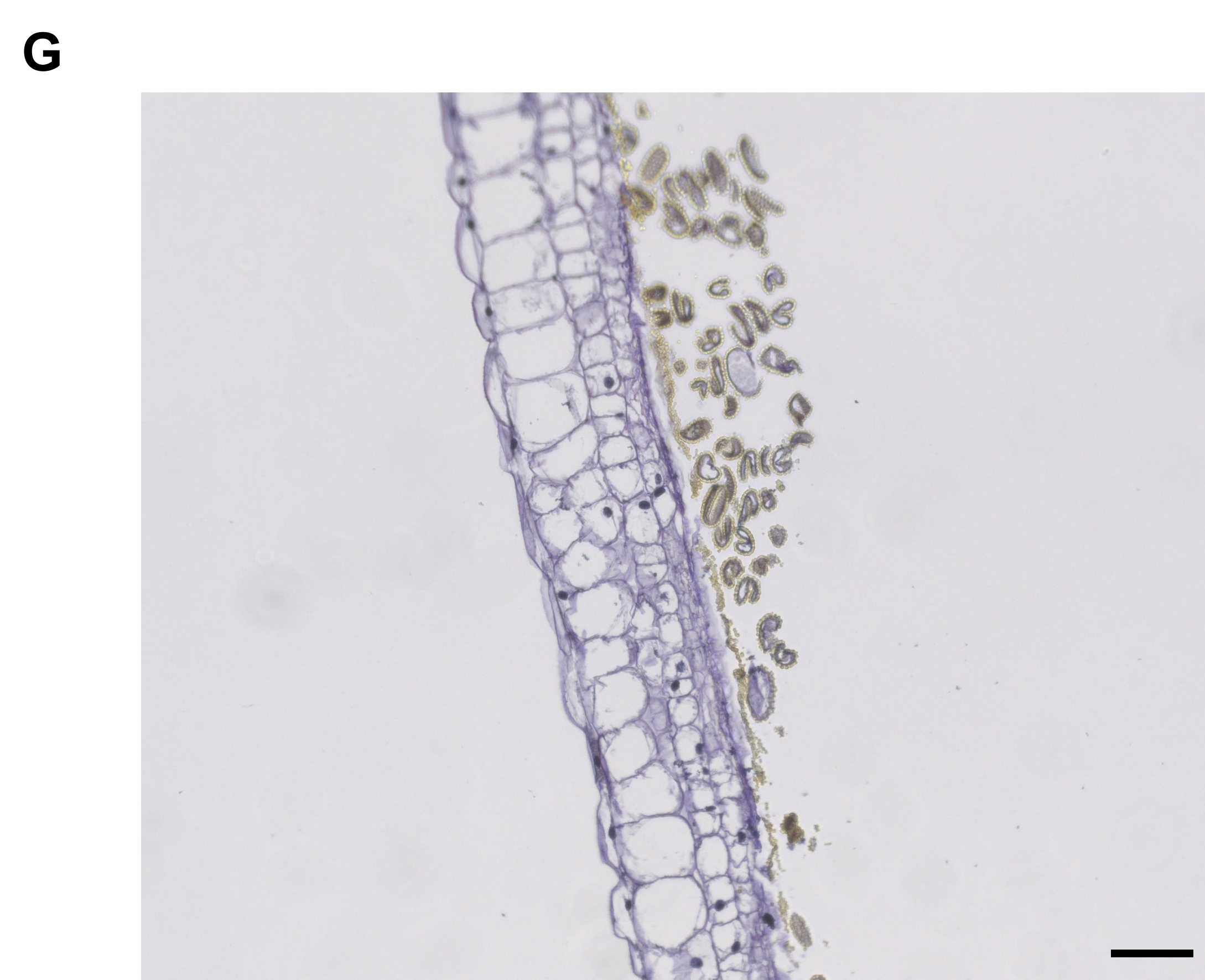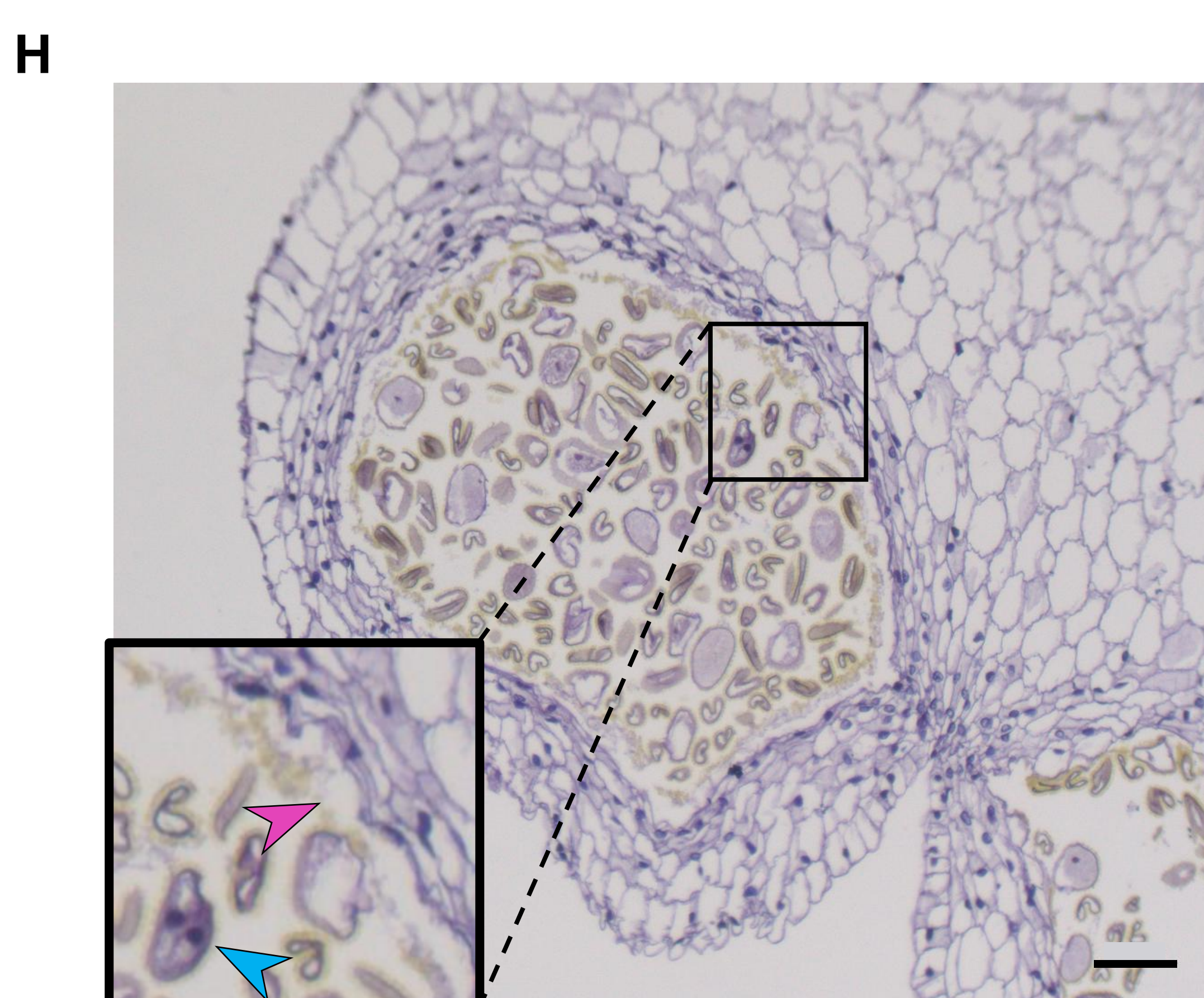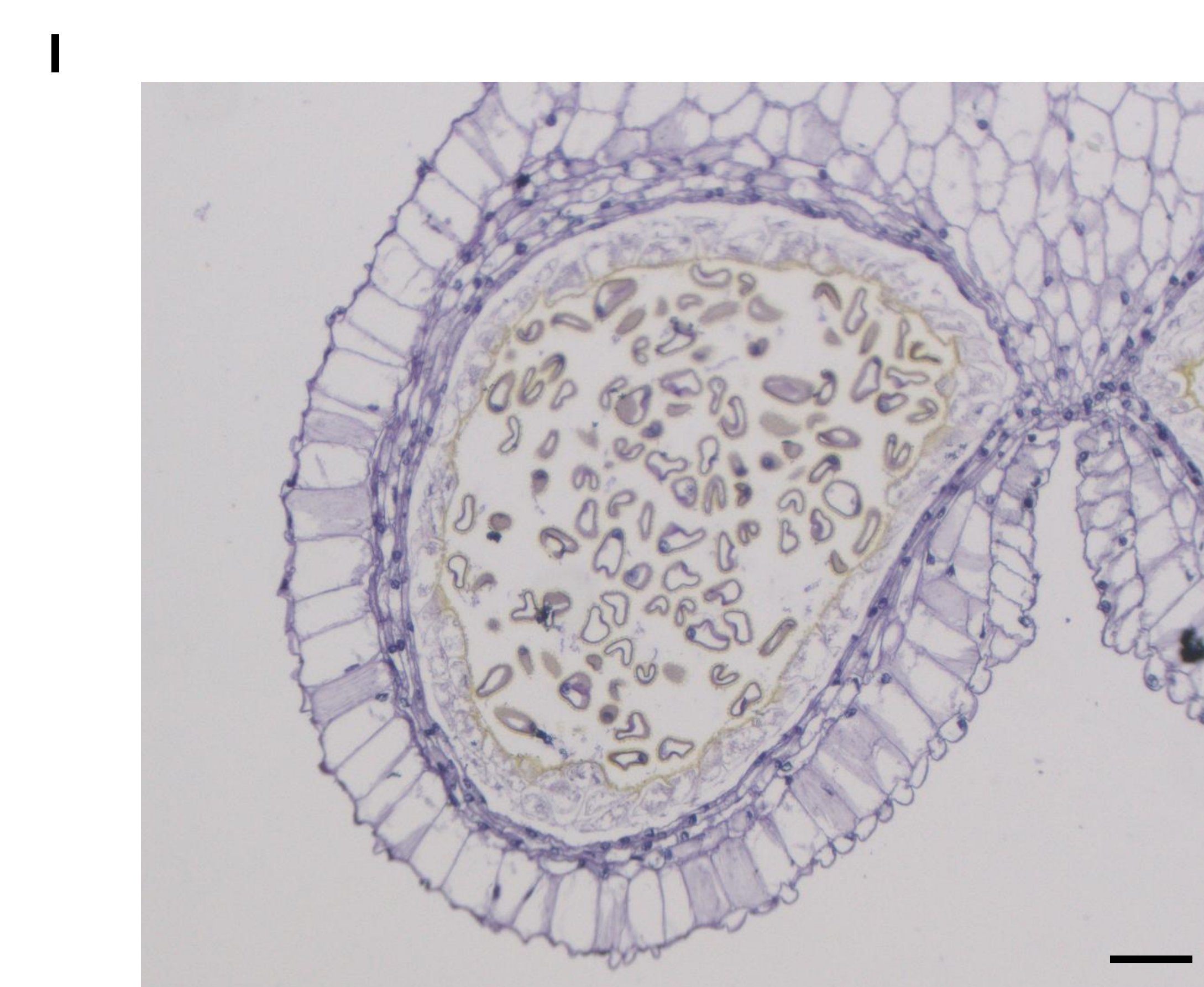

Supplement: Web_Material_uhae339 [file web_material_uhae339.zip › Fig. S5.pdf]

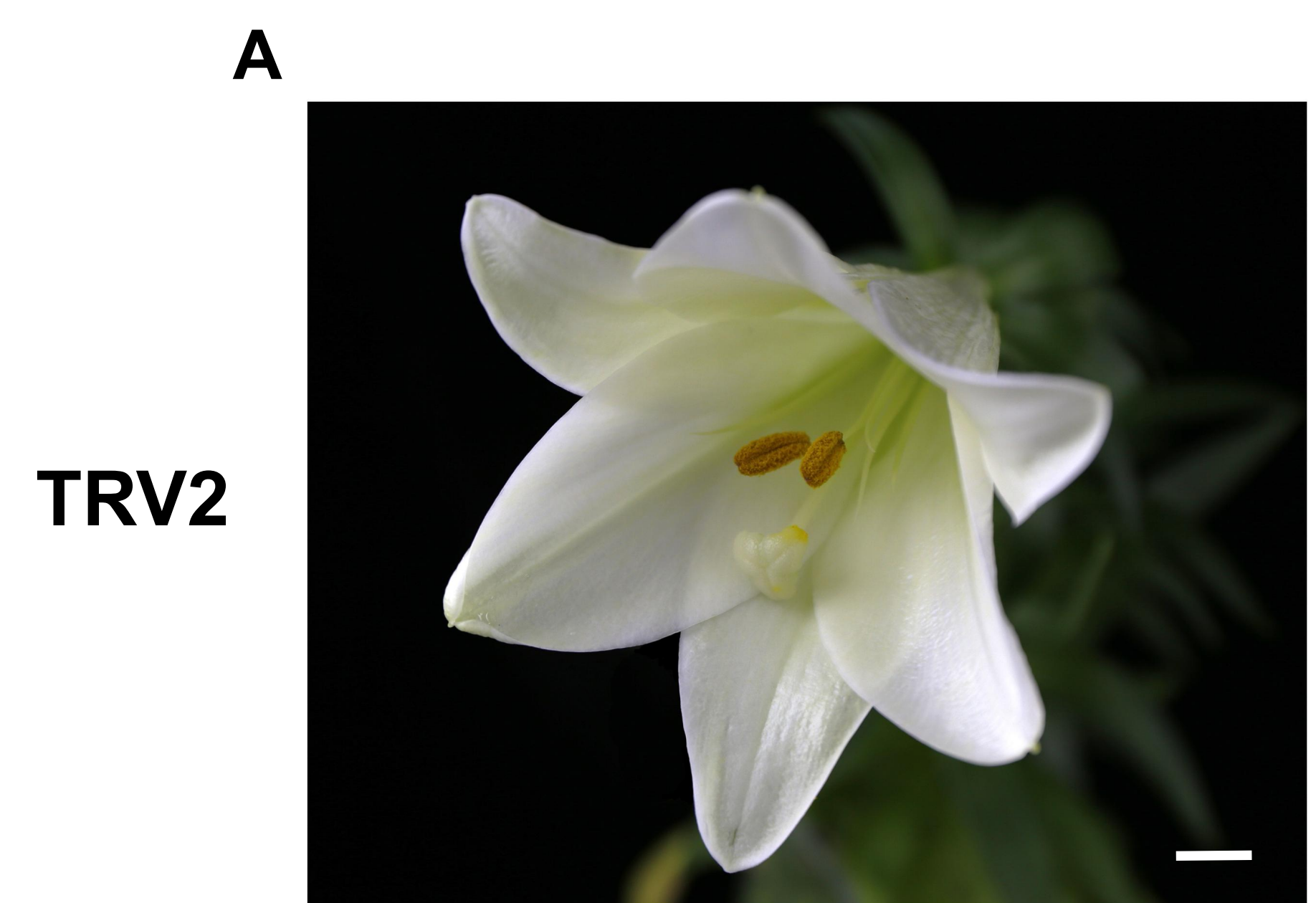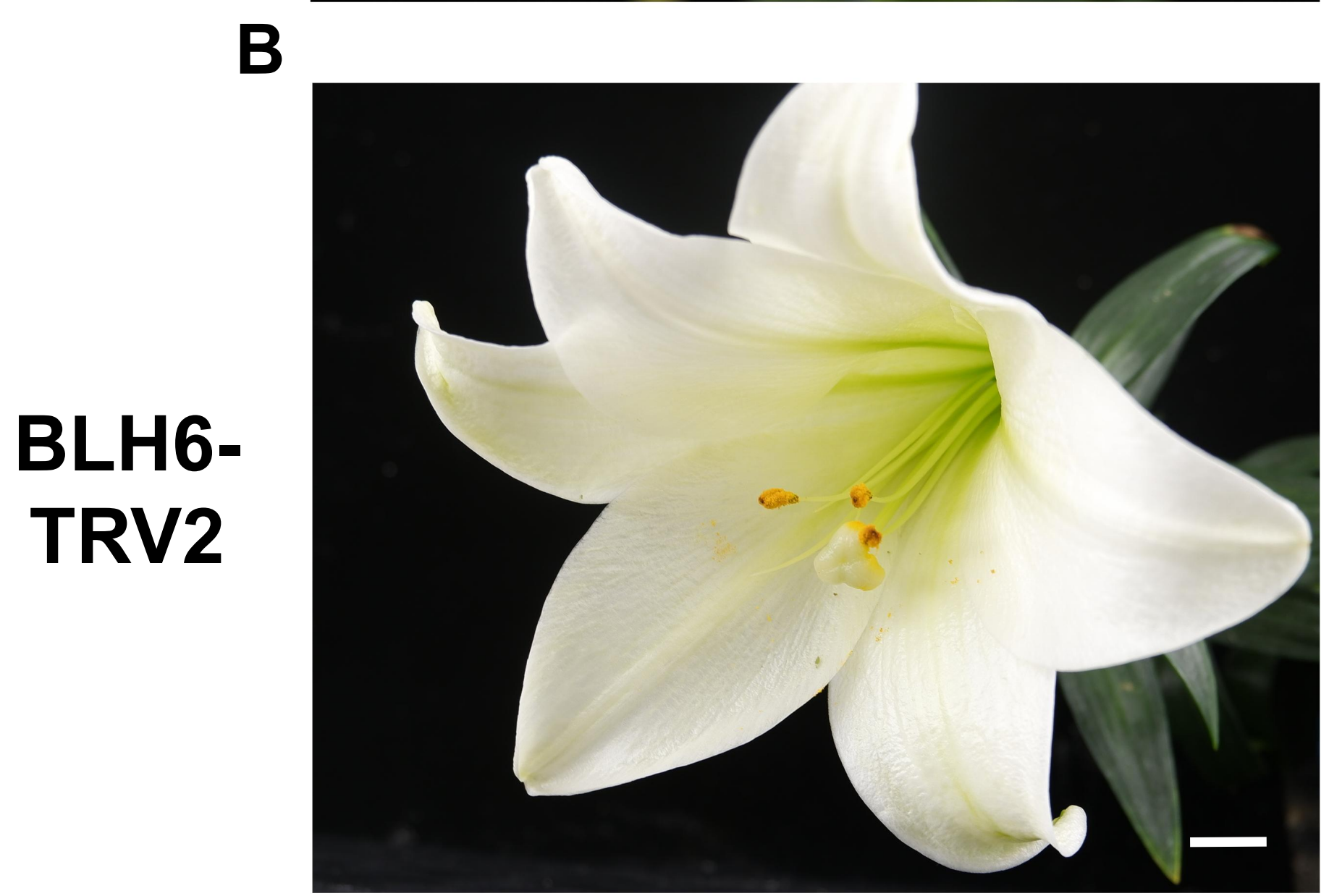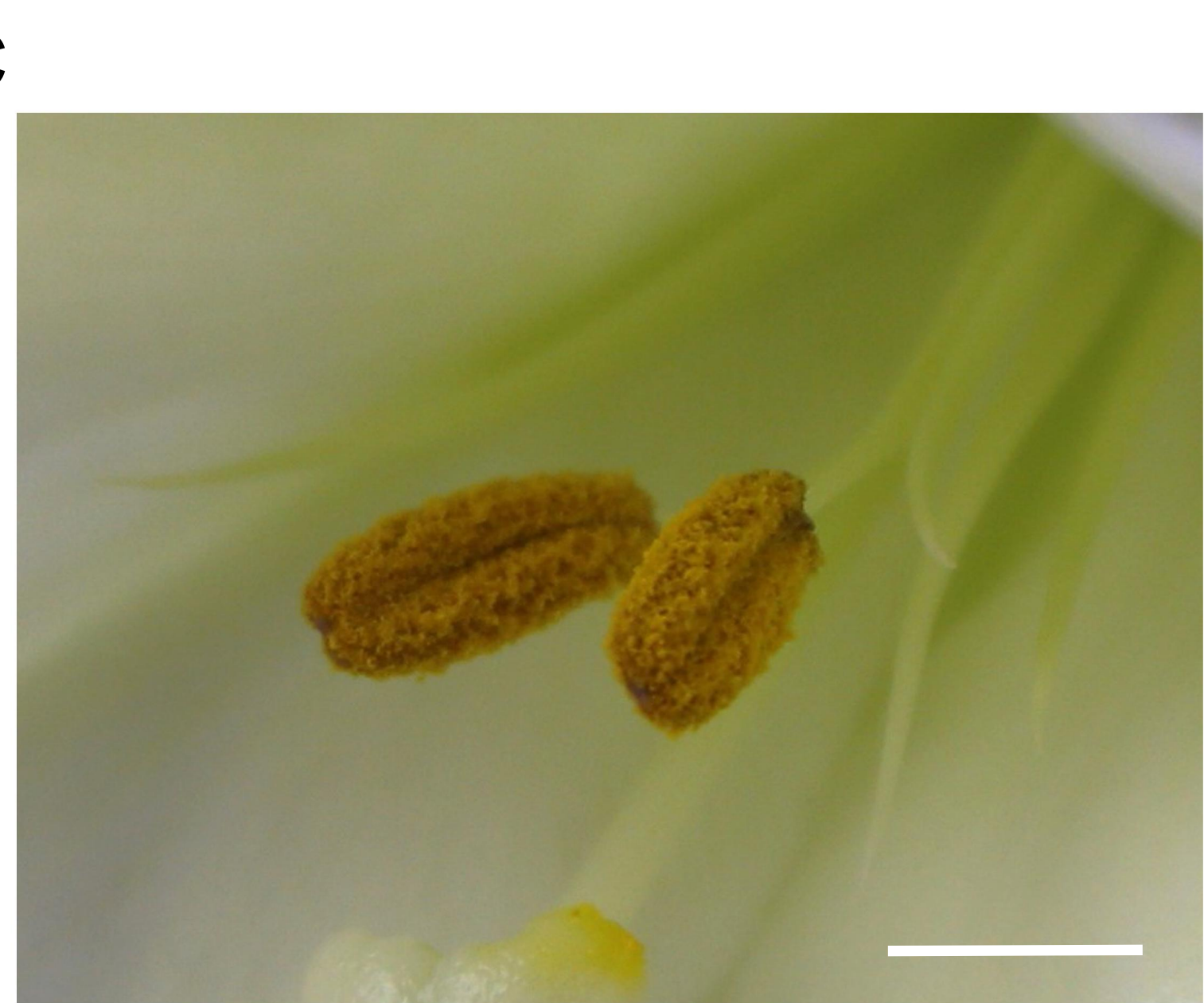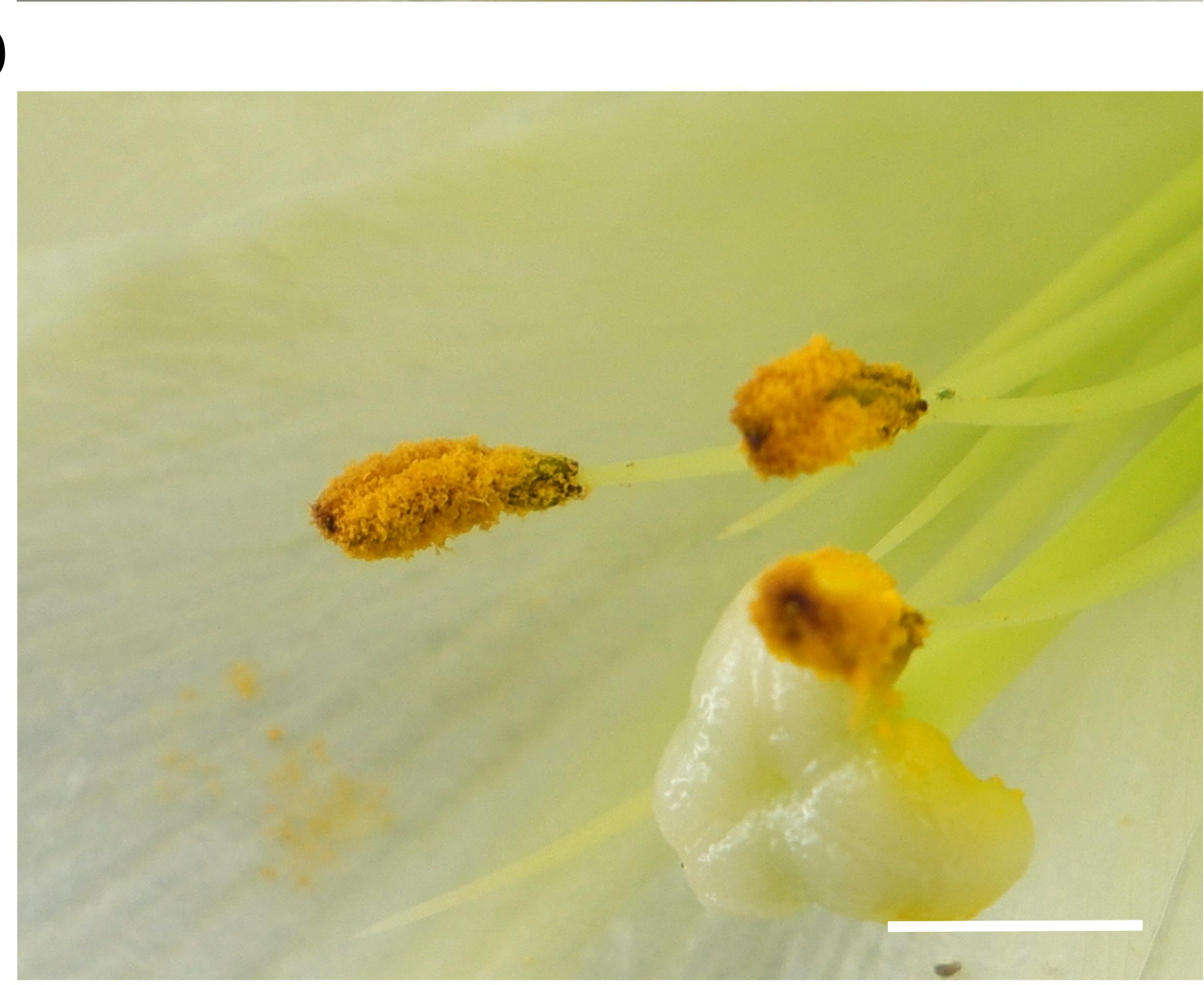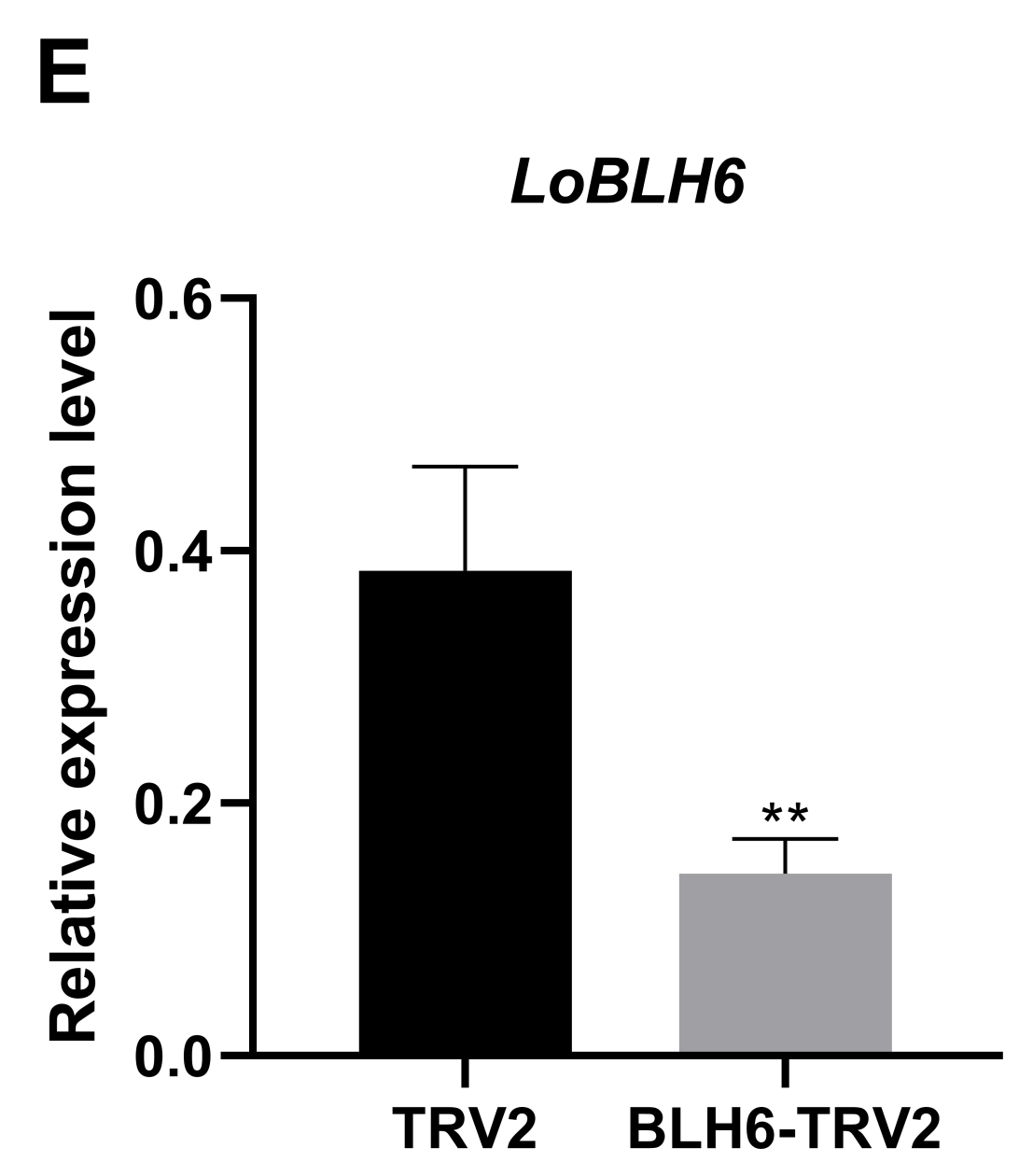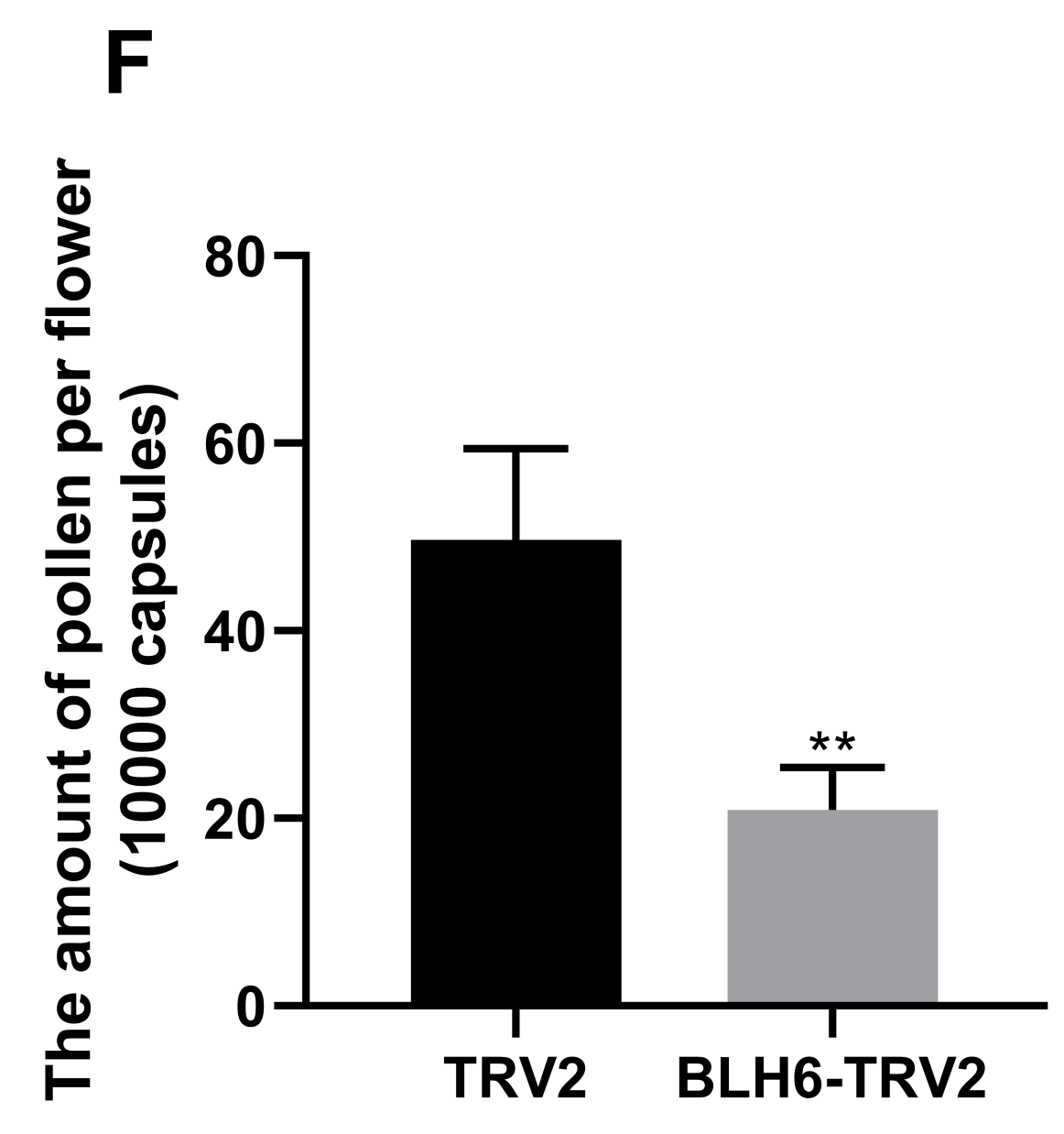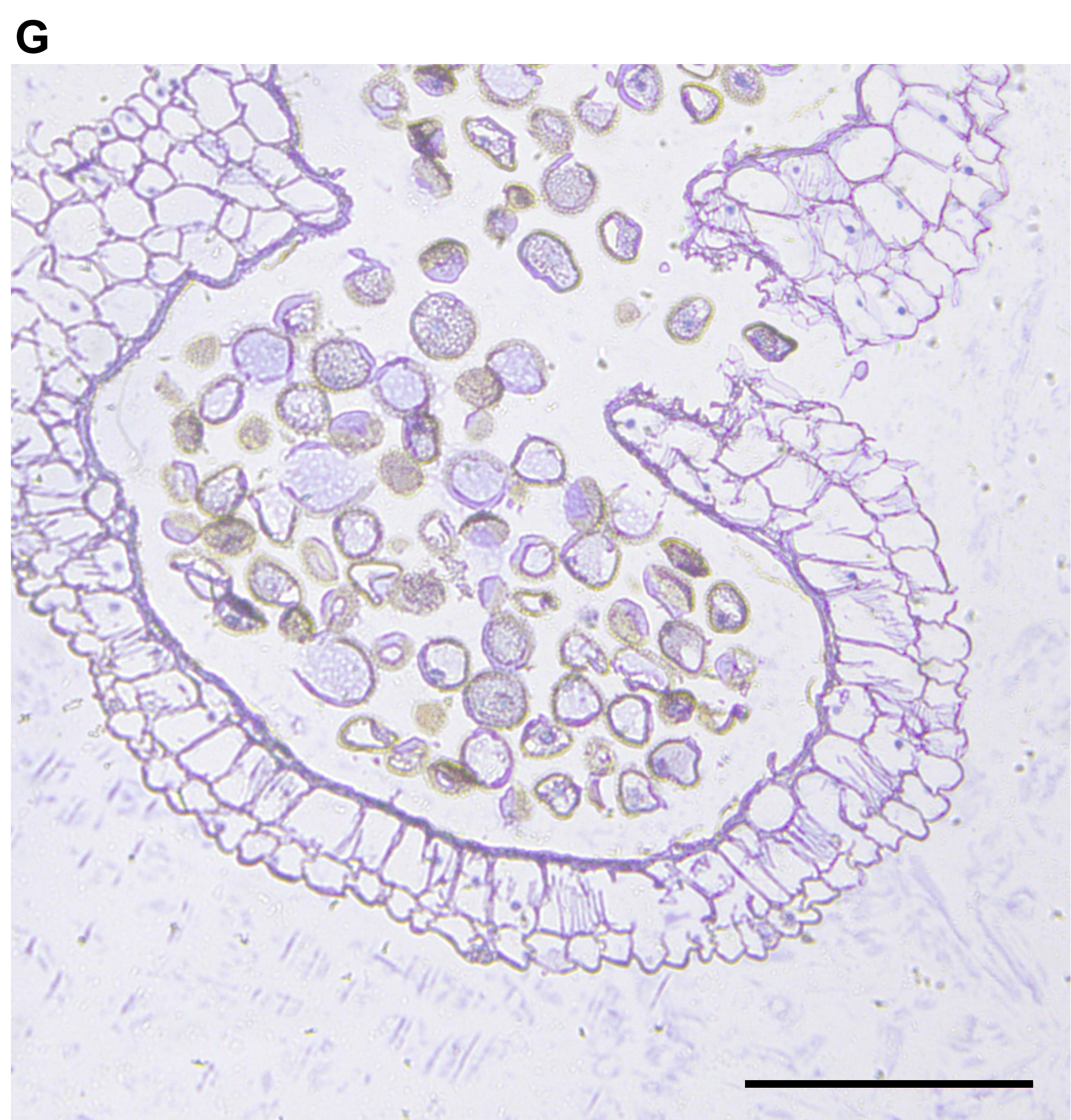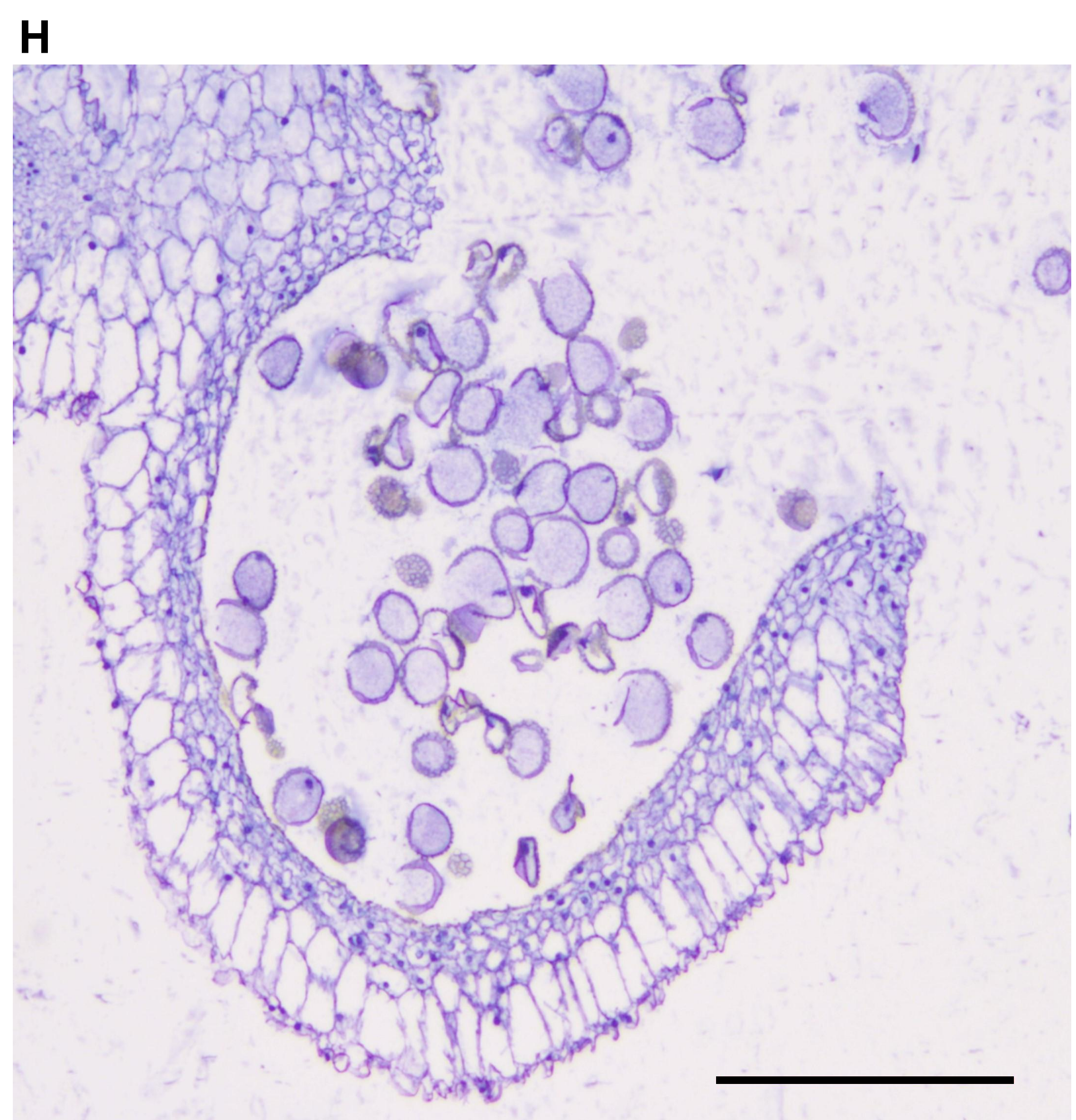

Supplement: Web_Material_uhae339 [file web_material_uhae339.zip › Fig. S6.pdf]

| AD     | BD      | -LW                                                                                  | -LWHA                                                                                 | X-α-gal                                                                               |
|--------|---------|--------------------------------------------------------------------------------------|---------------------------------------------------------------------------------------|---------------------------------------------------------------------------------------|
| T      | 53      | 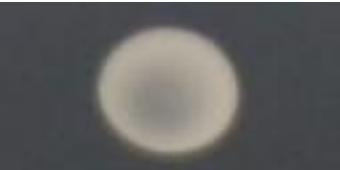   | 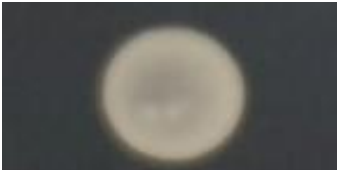   | 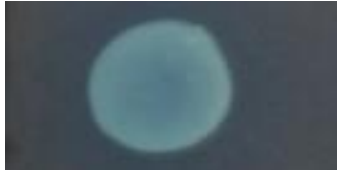   |
| —      | —       | 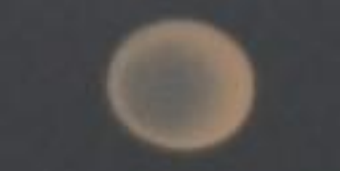 | 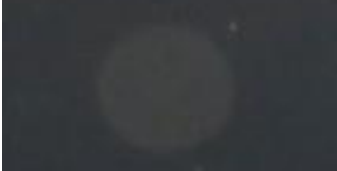 | 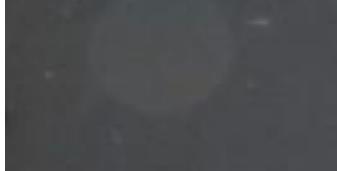 |
| —      | LoMYB33 | 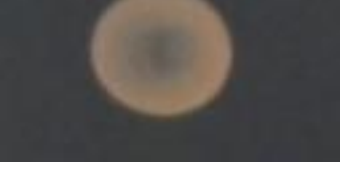 | 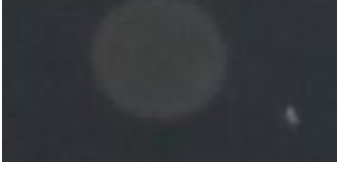 | 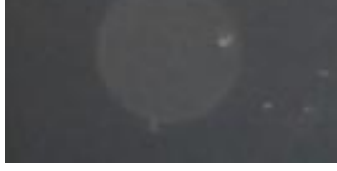 |
| LoBLH6 | —       | 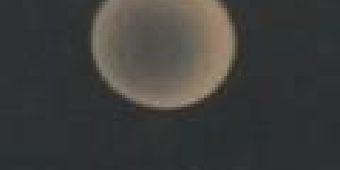 | 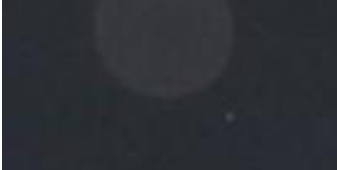 | 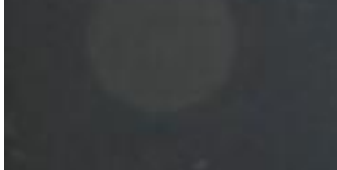 |
| LoBLH6 | LoMYB33 | 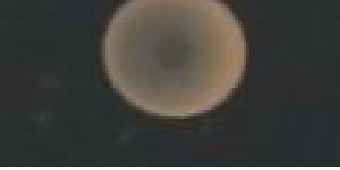 | 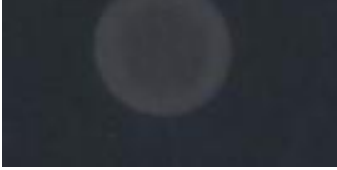 | 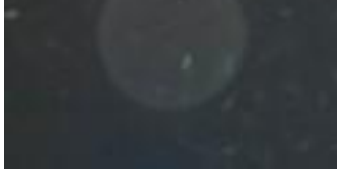 |

Supplement: Web_Material_uhae339 [file web_material_uhae339.zip › Fig. S7.pdf]

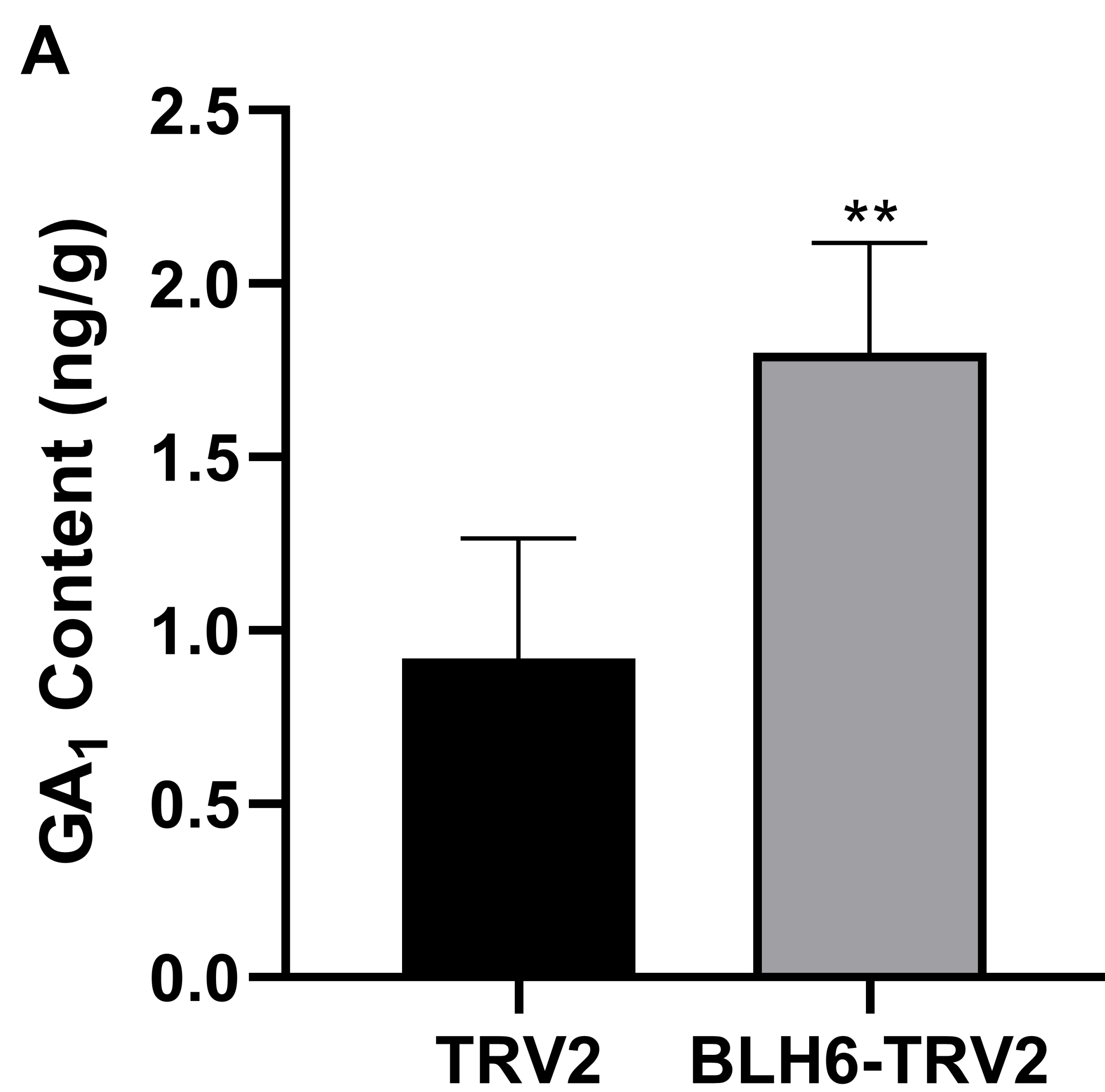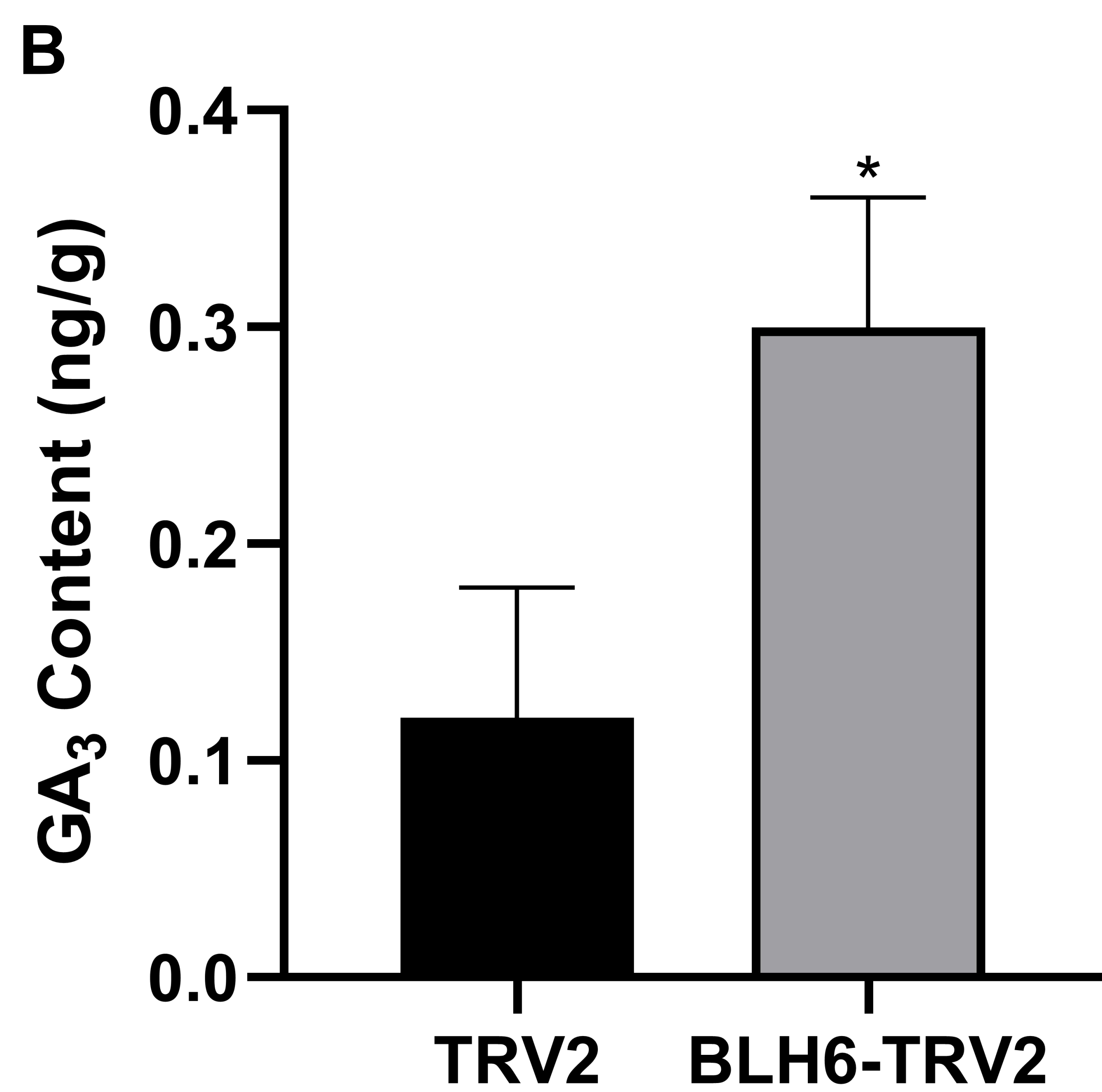

Supplement: Web_Material_uhae339 [file web_material_uhae339.zip › Fig. S8.pdf]
